# Optimizing Positive End-Expiratory Pressure in Asymmetric Acute Lung Injury in a Porcine Model: The Role of Transpulmonary Pressure

**DOI:** 10.3390/ijms26209985

**Published:** 2025-10-14

**Authors:** Claudine H. Mutschler, Benjamin Seybold, Stefan Aschauer, Nils Englert, Cleo-Aron Weis, Tanja Poth, Defne Cetiner, Mark O. Wielpütz, Dorothea Kehr, Markus A. Weigand, Armin Kalenka, Mascha O. Fiedler-Kalenka

**Affiliations:** 1 Medical Faculty, Department of Anesthesiology, University Heidelberg, Heidelberg University Hospital, 69120 Heidelberg, Germany; 2 German Center for Lung Research (DZL), Translational Lung Research Center Heidelberg (TLRC), 69120 Heidelberg, Germany; 3 Medical Faculty, Institute of Pathology, University Heidelberg, Heidelberg University Hospital, 69120 Heidelberg, Germany; 4 Center for Model System and Comparative Pathology (CMCP), Heidelberg University Hospital, 69120 Heidelberg, Germany; 5 Medical Faculty, Department of Diagnostic and Interventional Radiology, University Heidelberg, Heidelberg University Hospital, 69120 Heidelberg, Germany; 6 Institute of Diagnostic Radiology and Neuroradiology, University Medicine Greifswald, University of Greifswald, 17475 Greifswald, Germany; 7 Medical Faculty, Department of Molecular and Translational Cardiology, University Heidelberg, 69117 Heidelberg, Germany; 8 Internal Medicine III, Heidelberg University Hospital, 69120 Heidelberg, Germany; 9 District Hospital Bergstrasse, 69646 Heppenheim, Germany

**Keywords:** asymmetric acute lung injury, acute respiratory distress syndrome, mechanical ventilation, transpulmonary pressure, porcine model

## Abstract

Acute hypoxemic respiratory failure is a critical challenge in intensive care. A substantial proportion of patients present with asymmetric acute lung injury (ALI), complicating management due to heterogeneous lung involvement. While lung-protective mechanical ventilation represents the standard of care, the optimal approach to positive end-expiratory pressure (PEEP) titration remains unclear. This study investigated the effects of transpulmonary pressure (TPP)-guided PEEP titration vs. a fixed PEEP strategy in a porcine model of unilateral ALI. A total of 14 pigs underwent ALI induction via unilateral surfactant depletion and were randomized to receive either a fixed PEEP of 5 cmH_2_O or a PEEP targeting a slightly positive TPP at end-expiration. Over six hours, respiratory mechanics, high-resolution computed tomography (HRCT), histological lung injury scores (LIS), and plasma protein biomarkers were assessed. TPP-guided PEEP titration significantly lowered driving pressure and improved compliance compared to fixed low PEEP, suggesting more homogeneous tidal volume distribution. HRCT revealed less collateral injury in the initially non-injured lung in the TPP-guided group. However, histopathological LIS did not differ between groups. Exploratory cytokine profiling showed systemic inflammatory activation—including pro- and anti-inflammatory responses—only in the TPP-guided group. These findings indicate that TPP-guided PEEP titration may optimize ventilation by balancing alveolar recruitment and overdistension in asymmetric ALI, with clear effects on physiological and imaging parameters, but without parallel effects on cytokine responses. Further research is needed to assess its long-term impact and clinical relevance.

## 1. Introduction

Acute hypoxemic respiratory failure, with its most critical manifestation as acute respiratory distress syndrome (ARDS), is one of the most prevalent and severe pulmonary complications in critically ill patients, with mortality rates up to 46% in severe cases [1]. Approximately 23% of these patients initially present with asymmetric or unilateral acute lung injury (ALI), thus not fulfilling the Berlin definition of ARDS, as these cases lack generalized manifestations [2,3]. This distinct pathophysiological presentation poses a unique challenge compared to bilateral ARDS, as initially unaffected regions can, at best, be protected.

A lung-protective mechanical ventilation (MV) strategy, alongside prone positioning, represents the cornerstone of current ARDS management [4,5,6]. The benefits of limiting tidal volume (V_T_), plateau pressure (P_plat_), and driving pressure (ΔP) in both asymmetric/unilateral and bilateral ALI are well established [7,8,9,10]. However, optimal positive end-expiratory pressure (PEEP) titration, aiming to reduce atelectrauma, stress, and strain, thereby minimizing ventilator-induced lung injury (VILI), remains a subject of ongoing research [11,12,13]. In cases of asymmetric lung injury, PEEP can assist in reopening injured lung regions and improving oxygenation. But, at the same time, higher airway pressures may result in an excessive redistribution of lung volumes toward initially healthy lung areas with better compliance, bearing the risk of VILI through overdistension in previously unaffected regions [10,14].

Experimental research on ALI and ARDS in patients is challenging due to disease severity and heterogeneity [15]. Large animal models, such as pigs, have demonstrated significant translational value in this setting [16,17]. The American Thoracic Society (ATS) recommends assessing experimental ALI beyond clinical parameters, emphasizing tissue-level evaluation with histological lung injury scores (LIS) and plasma-derived biomarkers to better link clinical findings with lung damage and assess ventilation strategies [18]. In this context, a recently published study in pigs with a unilateral ALI model demonstrated that PEEP titration aiming for a slightly positive transpulmonary pressure at end-expiration (TPP_exp_) appears to improve the homogeneity of lung volume distribution between affected and unaffected lung areas without provoking overdistension [19]. However, respiratory mechanics and lung volumes were assessed only briefly after PEEP application, and no histological or biomarker-based analyses were performed. Thus, this study cannot evaluate long-term effects of TPP-guided PEEP titration on respiratory mechanics or its potential tissue-level benefits, both of which are crucial for evaluating its clinical relevance.

Therefore, we investigated the effects of different PEEP strategies in an asymmetric ALI model in pigs on respiratory mechanics, histological LIS, plasma-derived biomarkers, and CT-based morphological lung assessment over a period of six hours of invasive MV [18,20]. We hypothesized that PEEP titration according to a slightly positive TPP_exp_ will be superior to fixed lower PEEP settings in terms of reducing tidal stress and the severity of ALI—reflected in a lower LIS, fewer computed tomography (CT)-morphological correlates, and a reduced systemic inflammatory response at the level of plasma-derived biomarkers—in both the affected and non-affected lung.

## 2. Results

We included 17 pigs in the study. Following ALI induction, three pigs developed a pneumothorax, which was identified by ultrasound or CT. Since this condition could artificially influence respiratory parameters, these pigs were excluded from further analysis. The remaining 14 pigs were randomized into three groups: *ALI_PEEP 5* (unilateral ALI, fixed PEEP of 5 cmH_2_O, *n* = 6), *ALI_PEEP TPP_exp_* (unilateral ALI, PEEP targeting a slightly positive TPP_exp_, *n* = 6), and *sham_PEEP 5* (no ALI, fixed PEEP of 5 cmH_2_O, *n* = 2).

### 2.1. Respiratory and Hemodynamic Parameters

#### 2.1.1. Baseline

At baseline (prior to ALI induction), no significant differences in clinical parameters were detected between the study groups (see Table S1 Supplementary Material).

#### 2.1.2. t6 Results

Following six hours of experimental MV (t6), several significant differences in clinical parameters were observed between Groups ALI_PEEP 5, ALI_PEEP TPP_exp_, and sham_PEEP 5. Among various respiratory parameters, significant differences were found in P_plat_ (17.5 ± 0.6 vs. 19.8 ± 0.8 vs. 13.2 ± 1.7 cmH_2_O; *p* = 0.003), PEEP (5.0 ± 0.0 vs. 10.8 ± 0.9 vs. 5.0 ± 0.0 cmH_2_O; *p* < 0.001), ΔP (12.3 ± 0.7 vs. 9.0 ± 0.6 vs. 8.0 ± 1.6 cmH_2_O; *p* = 0.005), respiratory system compliance (23.2 ± 2.3 vs. 31.8 ± 1.4 vs. 36.5 ± 2.5 mL/cmH_2_O; *p* = 0.006), and TPP_exp_ (−2.6 ± 0.8 vs. 1.2 ± 0.7 vs. 1.8 ± 1.3 cmH_2_O; *p* = 0.008) (see Table 1).

#### 2.1.3. Intergroup Differences in Respiratory Parameters

At t6, Groups ALI_PEEP 5 and ALI_PEEP TPP_exp_ showed significantly higher P_peak_ (*p* = 0.003) and P_plat_ (*p* = 0.003) compared to sham_PEEP 5. Consistently, we observed a significantly higher mechanical power (MP) in Groups ALI_PEEP 5 and ALI_PEEP TPP_exp_ compared to sham_PEEP 5 (*p* = 0.004) (see Table 1).

According to randomization, Group ALI_PEEP TPP_exp_ exhibited a significantly higher PEEP compared to Groups ALI_PEEP 5 and sham_PEEP 5 (*p* < 0.001). Furthermore, Group ALI_PEEP TPP_exp_ displayed a slightly positive TPP_exp_ (1.2 ± 0.7 cmH_2_O), indicating that the targeted PEEP strategy was successfully implemented in this group. In contrast, Group ALI_PEEP 5 showed a significantly lower, negative TPP_exp_ compared to Group ALI_PEEP TPP_exp_ (*p* = 0.008). No significant differences in TPP_exp_ were observed between Groups ALI_PEEP TPP_exp_ and sham_PEEP 5 (see Table 1).

#### 2.1.4. ALI_PEEP 5 vs. ALI_PEEP TPP_exp_

Notably, significant differences between ALI_PEEP 5 and ALI_PEEP TPP_exp_ were detected for ΔP (*p* = 0.005) and compliance (*p* = 0.006) without any significant differences in P_peak_ or P_plat_. In contrast, no significant differences in transpulmonary pressure (ΔP_L_) or MP were found between ALI_PEEP 5 and ALI_PEEP TPP_exp_ (see Table 2). Regarding hemodynamic parameters, a significant difference was observed in global end-diastolic volume index (GEDI) between ALI_PEEP 5 and ALI_PEEP TPP_exp_ (*p* = 0.031) (see Table 1).

**Figure 1:**
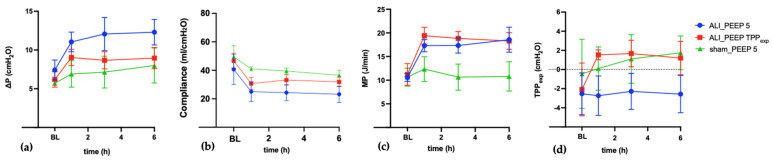
**Alterations of respiratory mechanics over 6 h of mechanical ventilation.** Panels illustrate (**a**) driving pressure (ΔP), (**b**) compliance, (**c**) mechanical power (MP), and (**d**) transpulmonary pressure at end-expiration (TPP_exp_) measured at baseline (BL) and after 2, 4, and 6 h of mechanical ventilation. Data are presented as mean ± SD for the study groups: ALI_PEEP 5 (blue circles), ALI_PEEP TPP_exp_ (red squares), and sham_PEEP 5 (green triangles). Abbreviations: ALI: acute lung injury, BL: baseline, cmH_2_O: centimeter of water, h: hours, J: joule, ml: milliliter, min: minute, MP: mechanical power, PEEP: positive end-expiratory pressure, SEM: standard error of the mean, TPP_exp_: transpulmonary pressure at end-expiration, ΔP: driving pressure.

The temporal evolution of key variables from baseline to t6 is illustrated in Figure 1, highlighting the differences in progression among the groups.

### 2.2. High-Resolution Computed Tomography Lung Injury Score

Due to technical reasons, one pig from Group ALI_PEEP 5 and one from the sham_PEEP 5 group were not transferred to the CT scanner. Consequently, statistical analysis of the high-resolution computed tomography (HRCT) scores was conducted only for Groups ALI_PEEP 5 and ALI_PEEP TPP_exp_, including five pigs in ALI_PEEP 5 and six in ALI_PEEP TPP_exp_. Representative images of the successfully scanned animal from the sham group are provided in the Supplementary Material.

Mean HRCT scores in ALI_PEEP 5 were 262 ± 11 (left) and 142 ± 7 (right) during expiratory hold, and 270 ± 35 (left) and 125 ± 4 (right) during inspiratory hold. In ALI_PEEP TPP_exp_, mean HRCT scores for expiratory hold were 260 ± 12 (left) and 121 ± 6 (right), while for inspiratory hold they were 268 ± 15 (left) and 116 ± 8 (right) (see Table 2).

**Figure 2:**
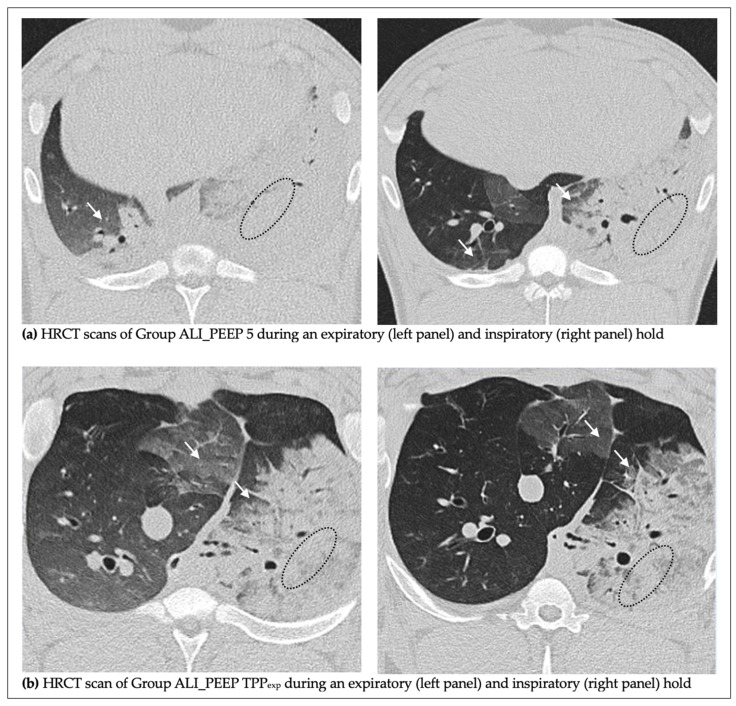
**CT scans of inspiratory and expiratory hold maneuvers after 6 h.** Representative axial high-resolution computed tomography (HRCT) scans during an expiratory and inspiratory hold after six hours of ventilation (t6) of the study groups: (**a**) ALI_PEEP 5, (**b**) ALI_PEEP TPP_exp_. In all images, the left lung represents the injured lung and the right lung represents the non-injured lung. (**a**) Group ALI_PEEP 5. The left panel shows an expiratory hold at PEEP 5 cmH_2_O, and the right panel an inspiratory hold at P_plat_ 30 cmH_2_O. Both lungs demonstrate consolidations (examples marked by dotted lines) and ground-glass opacities (arrows), with markedly greater involvement of the injured left lung where consolidations predominate. Following the inspiratory-hold maneuver, consolidations visually decrease in both lungs, with a more pronounced reduction in the non-injured right lung. (**b**) Group ALI_PEEP TPP_exp_. The left panel shows an expiratory hold at PEEP 5 cmH_2_O, and the right panel an inspiratory hold at P_plat_ 30 cmH_2_O. Again, the injured left lung demonstrates more extensive consolidations (dotted lines) and ground-glass opacities (arrows) compared to the non-injured right lung. Under TPP-guided PEEP, the inspiratory images suggest only a slight increase in aeration compared to the expiratory hold, with overall more homogeneous ventilation and reduced collateral involvement of the non-injured right lung compared to ALI_PEEP 5.

The varying extent of radiologically quantifiable lung injury between injured and non-injured lungs is demonstrated in representative CT scans shown in Figure 2.

#### 2.2.1. Left vs. Right Lung

**Figure 3:**
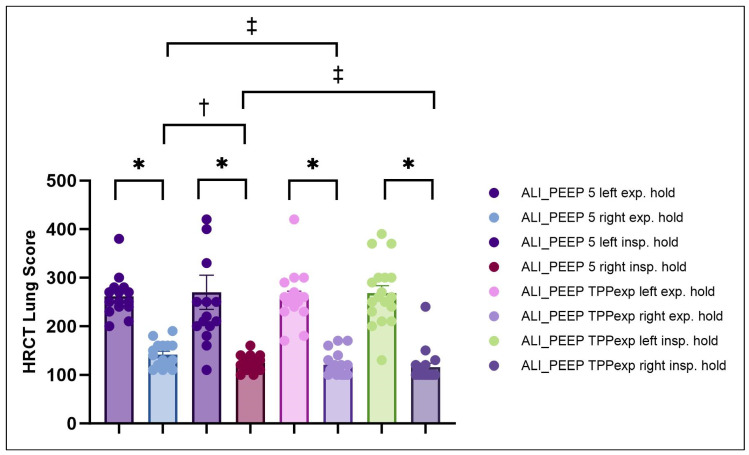
**HRCT lung injury score after six hours of mechanical ventilation.** HRCT lung injury scores of the right and left lungs in the ALI_PEEP 5 and ALI_PEEP TPP_exp_ groups following six hours of mechanical ventilation. Data are presented as mean ± SEM, with all individual data points displayed. * significant difference (*p* < 0.05) between left and right lungs. † significant difference (*p* < 0.05) between insp. hold and exp. hold within the same study group. ‡ significant difference (*p* < 0.05) between right lungs in ALI_PEEP 5 and ALI_PEEP TPP_exp_. Abbreviations: ALI: acute lung injury, exp.: expiratory, insp.: inspiratory, HRCT: high-resolution computed tomography, PEEP: positive end-expiratory pressure, SEM: standard error of the mean, TPP_exp_: transpulmonary pressure at end-expiration.

In both Groups ALI_PEEP 5 and ALI_PEEP TPP_exp_, the HRCT lung score was significantly higher in the left (injured) lung compared to the right (non-injured) lung during both the expiratory hold (ALI_PEEP 5: *p* < 0.001; ALI_PEEP TPP_exp_: *p* < 0.001) and the inspiratory hold (ALI_PEEP 5: *p* < 0.001; ALI_PEEP TPP_exp_: *p* < 0.001) (see Table 2 and Figure 3).

#### 2.2.2. Expiratory vs. Inspiratory Hold

The HRCT lung score of the left lungs did not differ between the expiratory and inspiratory hold maneuvers in either group (see Table 2 and Figure 3). However, within Group ALI_PEEP 5, a significant difference in the HRCT lung score of the right (non-injured) lung was observed between expiratory and inspiratory hold (*p* = 0.006), indicating a higher recruitability of the right lung, in contrast to ALI_PEEP TPP_exp_.

#### 2.2.3. ALI_PEEP 5 vs. ALI_PEEP TPP_exp_

The HRCT score of the left lung did not differ between experimental groups. In contrast, the HRCT score of the right (non-injured) lung was significantly higher in ALI_PEEP 5 compared to ALI_PEEP TPP_exp_ during both the expiratory hold (*p* = 0.015) and the inspiratory hold maneuvers (*p* = 0.016) (see Table 2 and Figure 3).

### 2.3. Histopathological Lung Injury Score

The mean modified LIS values in the left (injured) lung were 0.56 in ALI_PEEP 5, 0.54 in ALI_PEEP TPP_exp_, and 0.33 in sham_PEEP 5, respectively. In the right (non-injured) lung, the mean values were 0.30 in ALI_PEEP 5, 0.27 in ALI_PEEP TPP_exp_, and 0.32 in sham_PEEP 5, respectively. The results of the LIS analysis are summarized in Table 3, categorized by group and lung side.

#### 2.3.1. Left vs. Right Lung

**Figure 4:**
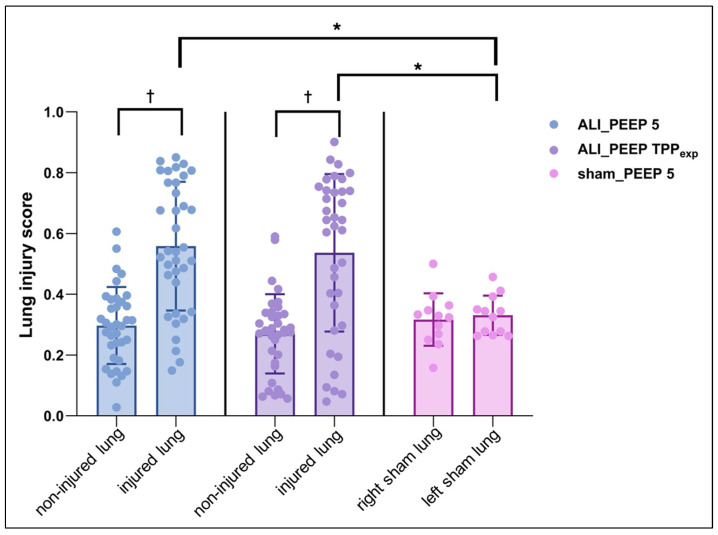
**Histopathological lung injury score after six hours of mechanical ventilation.** Histopathological lung injury scores of the right and left lungs in the study groups following six hours of mechanical ventilation. Data are presented as mean ± SEM, with all individual data points displayed. * significant difference (*p* < 0.05) between left lungs of different study groups. † significant difference (*p* < 0.05) between left and right lungs within the same study group. Abbreviations: ALI: acute lung injury, PEEP: positive end-expiratory pressure, SEM: standard error of the mean, TPP_exp_: transpulmonary pressure at end-expiration.

Groups ALI_PEEP 5 and ALI_PEEP TPP_exp_ showed significantly higher modified LIS values in the left lung compared to the right lung (*p* < 0.001). In contrast, in sham_PEEP 5 no significant difference in the modified LIS between the left and right lung was observed (see Table 3 and Figure 4).

#### 2.3.2. Intergroup Differences

A significant difference in the modified LIS among groups was observed for the left lung. Both ALI_PEEP 5 and ALI_PEEP TPP_exp_ exhibited significantly higher modified LIS values in the left (injured) lung compared to sham_PEEP 5 (*p* = 0.009). No significant differences were found in the modified LIS of the right lung among the groups (see Table 3 and Figure 4).

**Figure 5:**
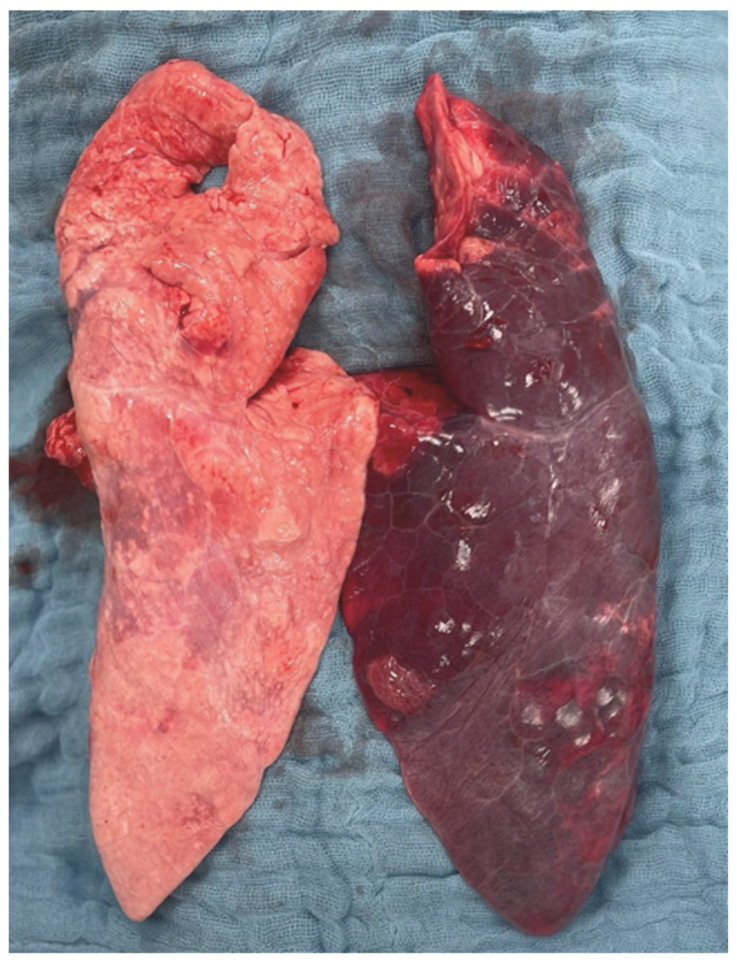
**Post mortem macroscopic image of both lungs.** Figure 5 shows a representative macroscopic image of both lungs from a pig in Group ALI_PEEP TPP_exp_ after unilateral ALI induction and six hours of mechanical ventilation. The right lung is displayed on the left side of the image, while the left lung is shown on the right.

**Figure 6:**
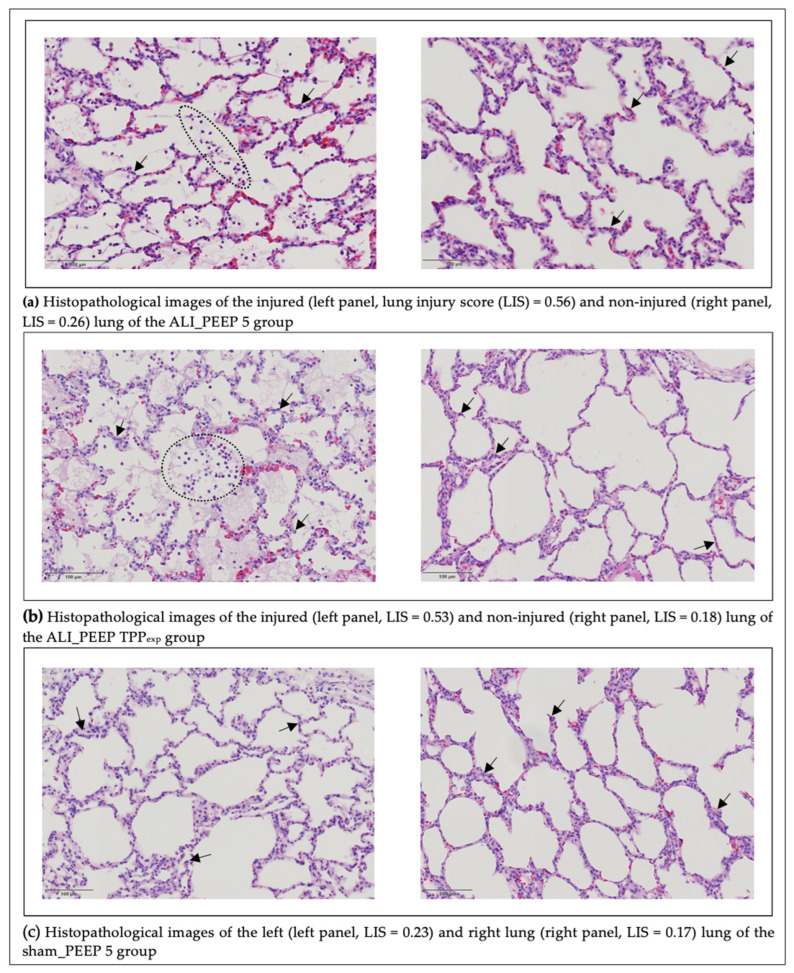
**Histopathological images of injured and non-injured lungs after six hours of ventilation.** Representative hematoxylin and eosin (HE) stained sections of lung tissue at 400× magnification from ALI_PEEP 5 (panel (**a**)), ALI_PEEP TPP_exp_ (panel (**b**)), and sham_PEEP 5 (panel (**c**)) groups. In each panel, the left image shows the left (injured in ALI_PEEP 5 and ALI_PEEP TPP_exp_) lung and the right image the right (non-injured) lung, with the corresponding lung injury scores indicated. Scale bar = 100 μm. In both unilateral ALI groups (panels (**a**,**b**)), the injured lungs exhibit intra-alveolar neutrophil infiltration (examples marked by dotted lines), intra-alveolar edema with eosinophilic deposits, and interstitial neutrophil granulocytes (arrows), with higher lung injury scores compared to the contralateral non-injured lungs. The non-injured lungs show only mild interstitial neutrophil infiltration. In contrast, in the sham_PEEP 5 group (panel (**c**)), both lungs display preserved alveolar architecture, no intra-alveolar neutrophils, and only a few interstitial neutrophils (arrows).

A macroscopic image of the post mortem-explored lungs is shown in Figure 5. Representative histological samples from each of the study groups are shown in Figure 6.

### 2.4. Exploratory Cytokine Profiling

For cytokine profiling, plasma samples were analyzed using the scioCD antibody microarray (Sciomics GmbH, Heidelberg, Germany), targeting 119 cytokines and 140 cell surface markers. Two t6 plasma samples from the ALI_PEEP 5 group could not be analyzed due to technical issues. Further methodology, including protein abbreviations and corresponding nomenclatures are provided in the Supplementary Study Report. The complete raw cytokine dataset has been deposited in the Mendeley Data repository (DOI: 10.17632/sbfpsgp42x.1).

#### 2.4.1. Protein Concentration

At baseline, bulk protein concentrations were similar across all groups (mean ± SEM; ALI_PEEP 5: 59.0 ± 3.8, ALI_PEEP TPP_exp_: 54.8 ± 1.9, and sham_PEEP 5: 54.0 ± 1.2 [mg/mL], respectively). At t6, concentrations remained stable in ALI_PEEP 5 and sham_PEEP 5, while lower values were observed in ALI_TPP_exp_ (mean ± SEM; ALI_PEEP 5: 53.2 ± 2.6, ALI_PEEP TPP_exp_: 43.8 ± 3.4, and sham_PEEP 5: 52.6 ± 2.2 [mg/mL], respectively).

#### 2.4.2. Protein Regulation

**Figure 7:**
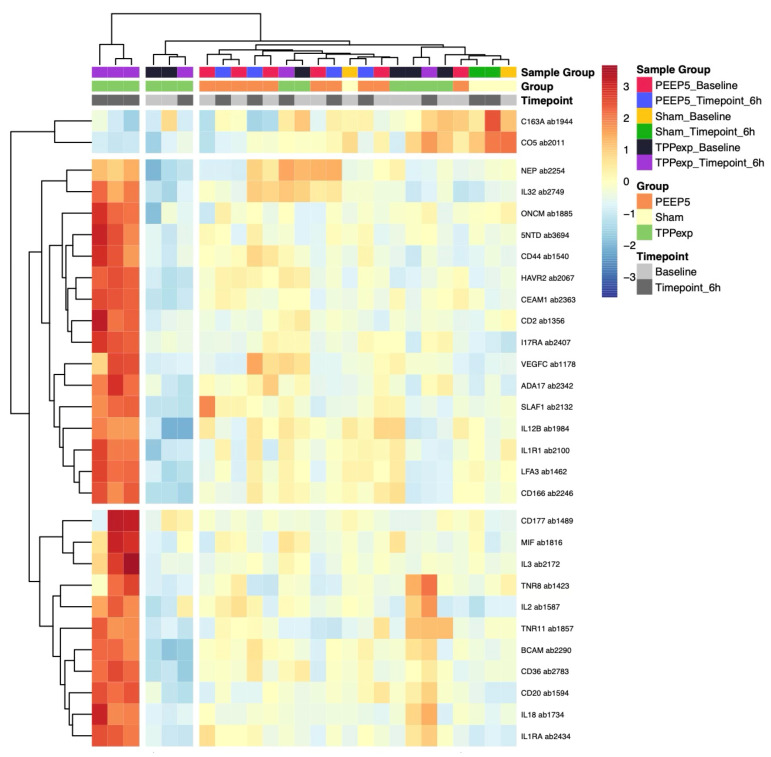
**Hierarchical clustering and functional annotation of differential proteins.** Figure 7 shows the clustering and heatmap visualization of proteins that were differentially expressed in at least one group comparison. Protein intensities were averaged across four technical replicates, centered and scaled per antibody. Functional pathway annotation is included for each protein. Three ALI_PEEP TPP_exp_ samples at timepoint 6 h form a distinct cluster, suggesting a specific inflammatory expression profile in this group. For protein names, see UniProt [21] and Supplementary Material.

In total, 29 proteins were identified as differentially expressed across groups and timepoints (|log2 Fold Change| > 0.5 and an adjusted *p*-value < 0.05) (see Supplementary Material). A hierarchical cluster analysis for differential proteins was performed to explore group-specific expression patterns. The resulting heatmap revealed that clustering was only partially evident, with a more distinct and consistent profile observed in ALI_PEEP TPP_exp_ compared to the other groups. Three ALI_PEEP TPP_exp_ samples tended to cluster together, suggesting a group-specific inflammatory response pattern (see Figure 7).

#### 2.4.3. Timepoint-Dependent Biomarker Regulation

To evaluate systemic biomarker dynamics over the course of the experiment, protein expression at t6 was compared to pooled baseline values across all groups. The sham_PEEP 5 showed no significant changes in any of the measured proteins, indicating stable biomarker profiles in the absence of injury. Interestingly, ALI_PEEP 5 similarly displayed no systemic significant time-dependent regulation, despite the presence of unilateral lung injury.

**Figure 8:**
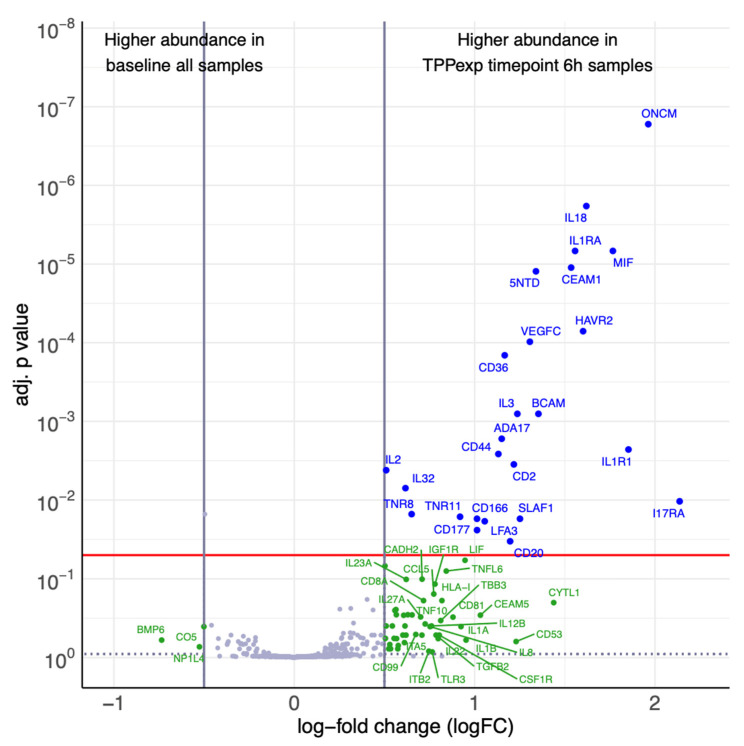
**Differential protein expression at baseline and t6 of ALI_PEEP TPP_exp_.** Figure 8 shows a volcano plot visualizing the differences in abundance between ALI_PEEP TPP_exp_ t6 samples and baseline all samples as log-fold changes (logFC) and their corresponding *p*-values (adjusted for multiple testing). The red line indicates the significance level of adjusted *p*-value = 0.05, vertical lines indicate the logFC cutoffs of ±0.5. A positive logFC indicates higher abundance in ALI_PEEP TPP_exp_ t6 samples, a negative logFC in baseline samples. Differential proteins (|logFC| > 0.5, adj. *p*-value < 0.05) are displayed with blue names. Nonsignificant proteins (adj. *p*-value < 0.9) with a |logFC| > 0.5 are defined as noteworthy and displayed with green names. For protein names, see Supplementary Material.

In contrast, ALI_PEEP TPP_exp_ exhibited a distinct temporal response, with a significant upregulation of multiple proteins at t6 (see Figure 8), including both pro-inflammatory (e.g., IL1R1, IL18, MIF, and IL32) and anti-inflammatory epithelial and endothelial markers (e.g., IL1RA and CEAM1). Additionally, Angiopoietin-2 levels were measured in all groups but did not show significant differences over time.

#### 2.4.4. Ventilation Strategy-Dependent Biomarker Regulation

**Figure 9:**
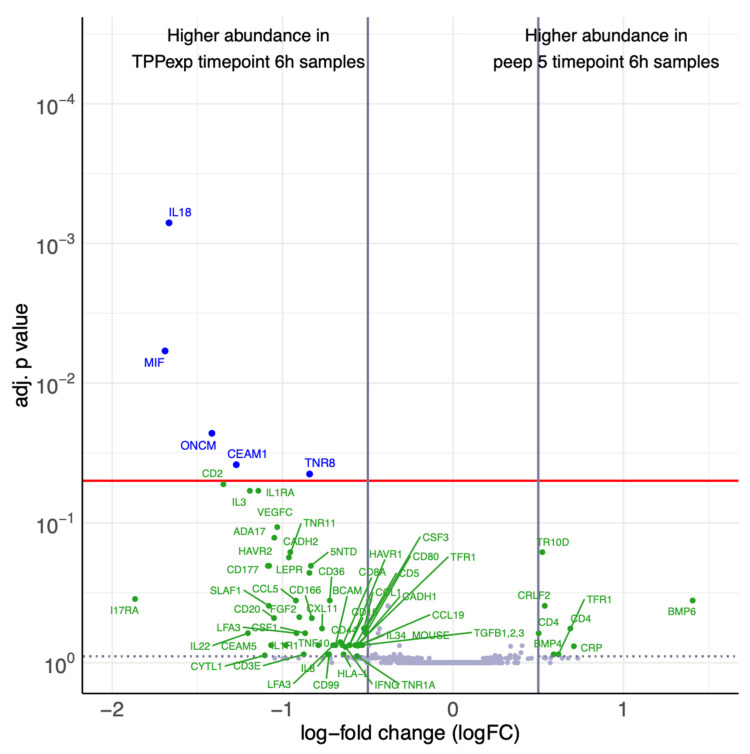
**Differential protein expression at t6 between ALI_PEEP 5 and ALI_PEEP TPP_exp_.** Figure 9 shows a volcano plot visualizing the differences in abundance between ALI_PEEP 5 vs. ALI_PEEP TPP_exp_ at t6 as log-fold changes (logFC) and their corresponding *p*-values (adjusted for multiple testing). The red line indicates the significance level of adjusted *p*-value = 0.05, vertical lines indicate the logFC cutoffs of ±0.5. A positive logFC indicates higher abundance in ALI_PEEP 5 t6 samples, a negative logFC in ALI_PEEP TPP_exp_ t6 samples. Differential proteins (|logFC| > 0.5, adj. *p*-value < 0.05) are displayed with blue names. Nonsignificant proteins (adj. *p*-value < 0.9) with a |logFC| > 0.5 are defined as noteworthy and displayed with green names. For protein names, see Supplementary Material.

To directly compare the two PEEP-strategies at the end of the experiment, protein expression profiles at t6 were analyzed between groups ALI_PEEP 5 and ALI_PEEP TPP_exp_. Five proteins, associated with inflammatory response, immune cell recruitment, and vascular activation showed significant upregulation in ALI_PEEP TPP_exp_ (see Figure 9). No proteins were found to be significantly upregulated in ALI_PEEP 5 compared to ALI_PEEP TPP_exp_. Additionally, Angiopoietin-2 levels did not significantly differ between the groups.

#### 2.4.5. Protein Interaction Network Analysis

**Figure 10:**
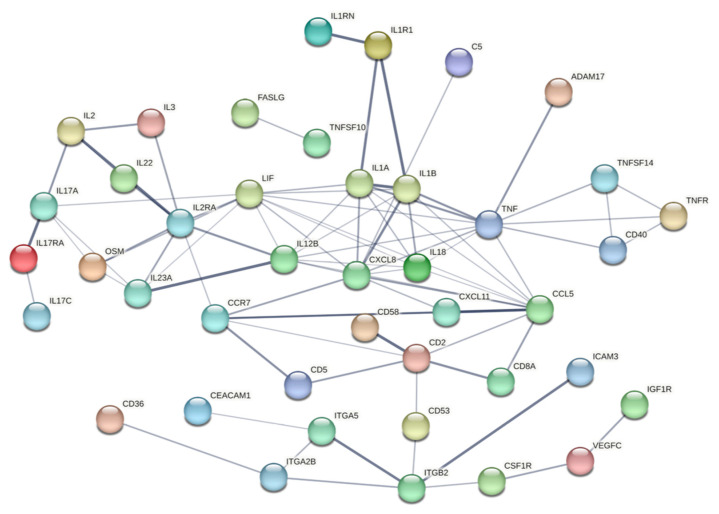
**Protein–protein interaction analysis of upregulated proteins.** Figure 10 shows the interaction network of selected proteins that were differentially or noteworthily expressed in at least one group comparison, generated using the STRING database. Proteins are color-coded based on functional pathway annotation (see Supplementary Material). The network highlights a dense cluster of immune-related mediators, including members of the cytokine—cytokine receptor interaction, JAK-STAT, IL17, TNF, and Toll-like receptor signaling pathways. Protein names are displayed in HGNC nomenclature.

To further explore the functional context of the differentially expressed proteins, a selected protein–protein interaction network analysis was performed using the STRING database including all proteins with abundance differences [22]. The resulting interaction map highlights central nodes and functional clusters within the regulated protein set, including pathways related to cytokine–cytokine receptor interaction, JAK-STAT signaling, IL17 signaling, TNF signaling, and NF-κB activation (see Figure 10).

## 3. Discussion

This study investigated the effects of two different PEEP strategies in a unilateral ALI model in pigs. The key findings can be summarized as follows: Six hours post-ALI induction (t6), a TPP-guided PEEP strategy (targeting a TPP_exp_ of 0–3 cmH_2_O) resulted in significantly lower ΔP and higher compliance compared to a fixed PEEP setting of 5 cmH_2_O, whereas ΔP_L_ and MP remained unchanged. At t6, the HRCT score was significantly higher in the left (injured) lung than in the right lung, regardless of the chosen PEEP strategy. Interestingly, the HRCT score of the right (non-injured) lung was significantly higher in the fixed PEEP 5 group than in the TPP-guided group during both expiratory and inspiratory hold maneuvers. Plasma-derived biomarker analysis showed significant changes at t6 only in the TPP-guided group, with upregulation of pro- and anti-inflammatory markers involving epithelial and endothelial activation pathways. The histopathological lung injury score at t6 showed significant differences between the injured and non-injured lungs but did not differ among groups.

### 3.1. Experimental Model

Many animal models of ALI aim to replicate the clinical conditions associated with ARDS in humans; however, none fully mimics the complexity of the human disease [17,18]. A “classical” model is the saline lavage model, which is highly recruitable and, therefore, may not be suitable for longer study durations [23]. To enhance ALI severity, a “second hit,” such as injurious high tidal volume ventilation, is often applied [23]. However, since high tidal volume ventilation alone can induce lung injury in the non-lavaged lung in unilateral models, we utilized a modified unilateral lavage protocol previously established that combines saline lavage with Triton X-100—a detergent further disrupting surfactant function [24,25]. Importantly, it must be acknowledged that the lavage procedure itself may introduce aspiration-related alterations. In our model, the relatively small saline volume (<500 mL) and the use of a validated protocol strongly suggest that Triton X–mediated surfactant depletion was the predominant injury mechanism. Nevertheless, the precise attribution of injury could be further clarified in future studies through the inclusion of a control group exposed to the same saline volume without Triton X. However, the Triton X-100 lavage model, established by Geilen and colleagues, appears well-suited for inducing a targeted and sustained lung injury without affecting the non-lavaged lung [25]. In line with this, six hours after ALI induction, our model showed persistently reduced compliance and significantly elevated airway pressures compared to both baseline and the sham group, confirming the prolonged impact of our lavage-induced injury at the level of physiological (respiratory) parameters. In brief, we aimed to establish a large animal model with high translational relevance, incorporating targeted lung injury and lung-protective ventilation to closely simulate the clinical conditions of human asymmetric ALI [8,18]. Thereby, the sham group was deliberately limited to two animals in accordance with the 3R principles, serving as a methodological reference rather than for inferential comparisons. While a larger group would have provided greater statistical robustness, the absence of relevant changes in these animals supports the validity of our findings and aligns with previous porcine ALI studies using similarly small reference groups [19,25,26].

On a histopathological level, we found characteristic features of early human ARDS, further confirming the successful implementation of ALI in our experimental setup [27,28]. Both intervention groups with unilateral ALI induction showed significantly higher LIS in the injured lung, reinforcing the asymmetric nature of lung injury in our model. In contrast, the sham-operated group displayed similar LIS values on both sides of the lung, corresponding to the LIS observed in the right (non-injured) lung of the intervention groups. However, these values are not truly “normal” and likely reflect the cumulative effects of multiple bronchoscopies, sedation, supine positioning, and prolonged MV.

Building on these histopathological findings, the hallmark feature of ALI is diffuse alveolar damage (DAD) [27,29]. Ichikado and colleagues demonstrated a strong correlation between histopathologic features of DAD and HRCT findings in pigs, linking radiological imaging to the progression of pathological phases [30]. Consistently, HRCT lung injury scores in our model were significantly higher in the injured lung compared to the non-injured lung in both ALI groups, further validating the successful unilateral ALI induction at the radiological level.

In summary, our experimental model demonstrated characteristic features of ALI that persisted for several hours, with both histological and radiological findings consistently confirming its asymmetric, unilateral nature. As recommended by the ATS, this approach enables a comprehensive evaluation of potential effects of different PEEP strategies across multiple levels, including physiological dysfunction, histological evidence of tissue damage, and inflammatory response [18].

Using this model, we compared two PEEP strategies to assess their effects on lung injury progression. We applied a fixed PEEP of 5 cmH_2_O in the *“standard care group”*, a setting widely used in mechanically ventilated patients [31]. Additionally, this setting prevents subtotal collapse, particularly in the injured lung, thereby mitigating excessive V_T_ redistribution toward initially unaffected lung areas, which otherwise poses a risk of overdistension [19]. In the other group, PEEP was adjusted hourly according to TPP_exp_ (target 0–3 cmH_2_O), following findings by Bastia et al., who demonstrated that shortly after a decremental PEEP trial, this approach resulted in the most homogeneous V_T_ distribution in asymmetric ALI without causing overdistension, supporting its role as an individualized best-PEEP strategy [19].

### 3.2. Respiratory Mechanics

Alveolar collapse is a hallmark of ALI and ARDS, occurring due to various factors and leading to multiple detrimental consequences for lung tissue [4,32]. The extent of atelectasis, represented by the proportion of recruitable lung regions, appears to correlate with mortality [33]. The atelectrauma resulting from alveolar collapse may be classified as non-ventilated lung injury (NVLI) [34]. In the context of MV, sufficient PEEP can partially reopen collapsed alveoli, thereby reducing atelectasis and mitigating NVLI [35,36,37]. At the same time, excessive PEEP settings carry the risk of VILI due to barotrauma or volutrauma [13]. This presents a challenge, as the risks of atelectrauma and overdistension stand in opposition, and both contribute to the overall biotrauma observed under invasive MV [23]. However, well-defined mortality-associated thresholds for ventilator settings have been established to minimize VILI, forming the cornerstone of lung-protective ventilation in ALI and ARDS [38]. Unlike the well-defined thresholds for VILI prevention, the optimal PEEP titration strategy remains unclear and has been described as the ‘search for the Holy Grail’ in MV [13,32,38].

Several approaches to optimal PEEP titration have been explored, including TPP_exp_-guided strategies [39,40], best-compliance-guided methods [41], best oxygenation-based approaches [42], computed tomography-based lung recruitment strategies [43,44], and electrical impedance tomography-guided strategies [19,45], among others. However, none of these methods have demonstrated a clear survival benefit in ARDS patients in large RCTs. Nevertheless, TPP_exp_-guided PEEP titration has shown promise in several smaller clinical studies [40,46,47]. In the context of asymmetric ALI, affected areas exhibit significantly reduced compliance, increasing the risk of excessive V_T_ redistribution toward unaffected regions [25]. To counteract this excessive redistribution, a TPP_exp_ close to zero provided the best balance between homogeneous V_T_ distribution and avoidance of hyperinflation [19]. However, this finding by Bastia et al. was limited to a short-term experiment. Based on this, we also observed improved respiratory mechanics with the TPP_exp_-guided PEEP strategy in our long-term asymmetric ALI model, as indicated by a significantly lower ΔP and better compliance compared to a fixed PEEP setting of 5 cmH_2_O. Both parameters reliably serve as surrogates for lung stress [9,48]. To achieve a slightly positive TPP_exp_, a significantly higher PEEP (approximately 11 cmH_2_O) was required in the ALI_TPP_exp_ group. Consequently, the PEEP 5 cmH_2_O group exhibited a significantly lower, more negative TPP_exp_ (approximately −3 cmH_2_O). Consistent with our findings, Sarge and colleagues demonstrated in a post hoc analysis of the EPVent-2 trial that PEEP titration resulting in a TPP_exp_ close to zero was associated with superior outcomes in human ARDS, even correlating with increased survival [40]. Interestingly, in our study, no significant differences were observed between the groups in MP and ΔP_L_, both of which are key determinants of VILI [48,49]. This suggests that the reduction in ΔP—identified by Amato et al. as the most critical respiratory parameter for reducing mortality—may offer a significant advantage of the TPP_exp_-guided PEEP titration [9]. Overall, in the ‘search for the Holy Grail’ of optimal PEEP, a compromise that mitigates atelectrauma without causing excessive overdistension appears promising. Our findings suggest that titrating PEEP to maintain a TPP_exp_ close to zero may be beneficial for determining the individually optimal PEEP, not only in short- but also in long-term experiments of asymmetric ALI.

### 3.3. HRCT Findings

High-resolution computed tomography remains the gold standard for radiological assessment of ALI [38]. Ichikado and colleagues developed a CT-based scoring system to assess ALI radiologically, showing that an HRCT score > 210 in early ARDS predicts higher 60-day mortality and increased risks of multiple organ failure [50,51]. In our study, the injured lungs in both ALI groups showed HRCT scores exceeding 260, indicating severe ALI. In contrast, HRCT scores in the non-injured lungs were significantly lower, highlighting the asymmetric nature of our model and reflecting findings from non-injured lungs in another unilateral ALI model by Bastia et al. [19]. Interestingly, in the fixed PEEP 5 group, we found significant recruitability in the non-injured lung, as indicated by a significantly lower HRCT score during inspiratory compared to expiratory hold. Gattinoni and colleagues previously established that the extent of potentially recruitable lung strongly correlates with ΔP and even mortality in ARDS patients [33]. In this context, our findings imply that individualized PEEP titration based on TPP_exp_ may offer advantages over a fixed low PEEP strategy, as evidenced by significant differences in lung recruitability and ΔP. Further supporting this assumption, six hours after ALI induction, the HRCT scores of the non-injured lung were significantly lower in the TPP-guided group compared to the fixed PEEP 5 group.

Overall, our findings suggest that within six hours, PEEP titration targeting a TPP_exp_ around zero may substantially attenuate the progression of damage, particularly in initially unaffected lung areas, likely due to an individualized balance between atelectrauma and overdistension [34,49]. However, adjusting ventilation settings solely based on CT morphology in early ARDS did not demonstrate any survival benefits in a previously published study [52]. In contrast, the risk of misclassifying patients to ARDS subtypes led to ventilator strategies that increased mortality. Nevertheless, CT imaging provides valuable insights into the diagnosis, extent, and prognosis of ARDS [38,51,53]. In our study, CT findings support the idea that PEEP management according to TPP_exp_ is a promising tool, particularly for protecting initially unaffected lung areas in asymmetric ALI.

### 3.4. Histopathological Findings

The ATS recommends evaluating experimental ALI models and treatment strategies beyond clinical parameters, emphasizing tissue-level evaluation [18]. Building on this, we refined an existing LIS to focus more on neutrophil infiltration, one of the earliest histological markers during the acute, exudative phase of ALI [20,28]. Simultaneously, we excluded two variables—hyaline membranes and proteinaceous debris—as they typically do not appear in the early exudative phase [54].

In our study, LIS was significantly higher in the injured lungs compared to the non-injured lungs across both ALI groups, confirming the successful induction of unilateral ALI at the tissue level. Correspondingly, the sham group exhibited no LIS differences between the left and right lungs. Our histopathological findings align with those of Geilen et al., who originally established the unilateral ALI model used in our study [25]. In contrast, an alternative unilateral ALI model lasting 48 h showed no LIS differences between injured and non-injured lungs [55]. However, this study utilized a different injury model (unilateral ligation of the pulmonary artery) and a modified LIS that accounted for hemorrhagic components, which were particularly prominent in the ligated lung.

Interestingly, in our model, LIS in the non-injured lungs did not differ among all groups, including the sham group, suggesting that the unilateral injury did not induce significant histopathological damage in the contralateral lung within six hours of MV. In contrast, Spinelli et al., using the pulmonary artery ligation model, observed a higher LIS in the non-ligated lung compared to a control group, indicating additional ALI-induced damage in the initially unaffected lung [34]. However, direct comparison is challenging due to key methodological differences, particularly the use of substantially higher V_T_. Consequently, the extent to which asymmetric ALI exacerbates tissue damage in initially healthy lung regions, as well as the most appropriate histopathological LIS for capturing the early ALI phase across different injury models, remains an area of ongoing research.

Additionally, in our study, no significant differences in the LIS were observed between the ALI groups in either the injured or non-injured lung, which could suggest that the applied PEEP strategies did not exert significantly different effects on tissue-level injury. Similarly, Haase et al. struggled to demonstrate significant LIS differences between PEEP strategies in their porcine ALI model, despite clear effects on clinical outcomes [56]. In contrast, various experimental ALI models have demonstrated a mitigated inflammatory response through appropriately titrated PEEP, including at the tissue level [42,57,58]. Focusing on another key respiratory parameter for VILI, Araos and colleagues found a positive correlation between MP and LIS [59]. This may explain why the different PEEP strategies in our study did not lead to more pronounced histological differences, as MP was comparable in both groups and may have a greater impact on tissue-level injury than PEEP alone. Moreover, even the low PEEP in our ALI_PEEP 5 group may have helped to mitigate additional atelectrauma that might otherwise have resulted from NVLI under zero-PEEP. However, using our LIS, we could not detect significantly different effects of the compared PEEP strategies at the tissue level.

### 3.5. Exploratory Cytokine Profiling

The exploratory cytokine profiling revealed a striking divergence in systemic inflammatory responses between the experimental groups. Surprisingly, both the ALI_PEEP 5 and the sham_PEEP 5 group showed stable cytokine profiles throughout the experiment. In contrast, animals ventilated with the TPP-guided PEEP strategy exhibited a distinct systemic response after six hours, marked by upregulation of both pro-inflammatory and anti-inflammatory mediators. Many of the regulated cytokines are known to originate from pulmonary epithelial and endothelial cells, suggesting that the applied ventilation strategy influences cellular stress responses at the alveolar-capillary barrier [60,61]. This is further supported by hierarchical clustering and protein–protein interaction network analysis, which revealed a consistent cytokine expression profile in the ALI_PEEP TPP_exp_ group with functional clustering within inflammatory signaling pathways.

At first glance, the TPP-guided PEEP strategy appears to provoke a stronger inflammatory response. However, this interpretation seems questionable in light of prior data from Geilen and colleagues, who originally established the lung injury model used in our study and reported increasing levels of pro-inflammatory cytokines in broncho-alveolar lavage fluid (BALF) at PEEP levels of 5 to 7 cmH_2_O [25]. Moreover, atelectrauma—particularly expected when PEEP is set below TPP—leads to enhanced lung inflammation [36,62,63]. Additionally, as demonstrated by Bastia et al., greater atelectasis in asymmetric ALI may lead to tidal volume redistribution toward non-injured regions, increasing the risk of volutrauma and thereby further amplifying the inflammatory response, as shown by Guldner and colleagues [19,64]. However, the phenomenon of absent systemic inflammation during the early phase of an atelectasis-favoring ventilation strategy has been described previously and attributed to a compartmentalization of lung injury [65,66]. Therefore, the more pressing question may be why a TPP-guided strategy leads to such an early and clearly detectable systemic cytokine response? One possible explanation for the observed cytokine signature in the ALI_PEEP TPP_exp_ group is that it reflects a transient early phase of mechanotransduction-mediated systemic immune activation, which—if controlled—might precede a more favorable resolution phase [60,63]. The higher airway and transpulmonary pressures applied in this group could have induced greater cellular stretch, triggering local cytokine release and systemic translocation [67]. In contrast, the lack of systemic inflammation in the ALI_PEEP 5 group may reflect delayed or attenuated responses due to atelectasis-associated hypoperfusion or impaired mediator clearance in the early phase. Thus, the absence of differentially expressed proteins in the ALI_PEEP 5 group should not be interpreted as a methodological limitation, but rather as a biologically plausible finding. This observation is consistent with the concept of a compartmentalized inflammatory response in atelectatic, hypoperfused lung regions, where mediator release and systemic translocation may be attenuated despite relevant local injury. Supporting this, Wu and colleagues observed lower pro-inflammatory cytokine levels within the lungs after four hours of TPP-guided ventilation compared to other PEEP strategies—highlighting a local suppression that contrasts with the systemic upregulation seen in our study [42]. This suggests that, despite elevated systemic levels, pulmonary cytokine concentrations may be lower under a TPP-guided PEEP strategy —indicating a localized, compartmentalized response. Given the limited observation period, the emergence of a later atelectrauma-driven inflammatory phase cannot be assessed and should be addressed in future studies with extended follow-up.

The STRING-based protein interaction analysis supported the biological plausibility of these findings by demonstrating enrichment of classical inflammatory pathways such as IL-1, TNFα, NF-κB, and JAK-STAT signaling [22,68]. This supports the view that the observed cytokine alterations in the ALI_TPP_exp_ group are not random, but rather reflect a coordinated immune response potentially relevant to the evolution of lung injury.

Taken together, the cytokine findings suggest that ventilation with TPP-guided PEEP settings induces a coordinated inflammatory response that becomes detectable systemically. Why this systemic cytokine activation was absent after six hours of ventilation with a fixed low PEEP strategy, and whether a differentiated PEEP approach can modulate systemic inflammation, remain to be clarified. It should also be noted that the cytokine profiling was designed as an exploratory analysis. Accordingly, these findings should be interpreted as hypothesis-generating rather than as definitive evidence, and larger follow-up studies will be required to validate the observed patterns.

### 3.6. Limitations

This study has several limitations. First, while the porcine model offers translational relevance, species-specific differences in lung physiology and anatomy may limit applicability to humans. Second, the six-hour observation period primarily reflects the exudative phase of ALI and may not capture longer-term effects of PEEP titration. While the lavage procedure itself may have contributed to aspiration-related changes, we consider surfactant depletion by Triton X the predominant injury mechanism; future studies could clarify this by including a saline-only control group. Third, although TPP-guided PEEP optimizes alveolar recruitment and limits overdistension, the absence of direct regional strain assessments—such as imaging-based aeration analysis—restricts mechanistic insight. Fourth, reliance on a standardized, semi-quantitative lung injury score may overlook subtle tissue-level differences. Moreover, as systemic inflammatory markers were only assessed exploratorily, findings remain difficult to interpret. Another limitation is the small sham group size (*n* = 2), which precludes statistical inference but was deliberately chosen in line with the *3R principles* to minimize animal numbers. Future studies should address long-term outcomes, integrate advanced imaging, and include molecular analyses of tissue damage to refine individualized PEEP strategies.

## 4. Materials and Methods

### 4.1. Study Settings

This prospective, experimental, randomized, controlled trial included 17 domestic male and female pigs (German Landrace, 4–5 months of age, 50 ± 6 kg) and incorporated blinded proteomics and histo- and radiological assessments. During a six-hour experimental phase following unilateral ALI induction, the animals were randomized to different ventilation strategies to assess the impact of PEEP settings on the progression of ALI.

### 4.2. Ethics and Registry

All procedures adhered to German animal welfare regulations and were approved by the local animal research committee (Regierungspräsidium Karlsruhe, No. G-123/23, approved on 4 September 2023). The animals were housed at the interfaculty biomedical facility of the University of Heidelberg, Germany, and sourced from a local pig breeder.

### 4.3. Animal Preparation

Following overnight fasting with free access to water, pigs were initially anesthetized intramuscularly using a combination of 7 mg/kg azaperone (Stresnil^®^, Lilly, Bad Homburg, Germany), 5 mg/kg ketamine hydrochloride (Ketamine 10%, Bremer Pharma, Warburg, Germany), and 0.3 mg/kg midazolam (Midazolam, Hameln Pharma, Hameln, Germany). After intravenous catheter placement, anesthesia was maintained via continuous infusion of 12 mg/kg/h ketamine, 3.6 mg/kg/h midazolam, and 4–6 mg/kg/h propofol (Propofol 2%, Fresenius Kabi, Bad Homburg, Germany). The depth of anesthesia was routinely monitored by assessing the absence of spontaneous breathing and muscle tone. A tracheotomy was performed, followed by the insertion of a modified 35 French left-sided double-lumen tube (DLtube) (Shiley^®^, Medtronic GmbH, Meerbusch, Germany) as previously described [25]. Correct positioning of the DLtube was confirmed via bronchoscopy (Ambu^®^ aScope^®^ 4 Broncho, Bad Nauheim, Germany). Pigs were mechanically ventilated using an ELISA800^®^ ventilator (Löwenstein Medical, Bad Ems, Germany) with an inspiratory oxygen concentration (FiO_2_) of 0.4 in a volume-controlled mode. Ventilation parameters across all groups included a tidal volume of 6 mL/kg body weight (bw), an inspiration-to-expiration (I:E) ratio of 1:2, a respiratory rate (RR) of 24/minute, and a PEEP of 5 cmH_2_O. Lung recruitment was carried out three times, each time applying a driving pressure of 30 cm H_2_O for 15 s.

A central venous catheter (Logicath^®^, Smiths Medical, Grasbrunn, Germany) was inserted into the jugular vein, and a femoral PiCCO^®^ arterial catheter (PiCCO^®^, Pulsion Medical Systems, Feldkirchen, Germany) was placed for continuous hemodynamic monitoring. Body surface area was calculated following standard recommendations for pigs [69]. To measure esophageal pressures (P_ES_), an esophageal balloon catheter (Löwenstein Medical, Bad Ems, Germany) was inserted and positioned as previously described [70].

P_ES_ served as a surrogate for dorsal pleural pressure, as previously validated in unilateral ALI [19]. A midline suprapubic cystostomy was performed for urinary drainage.

Baseline respiratory and hemodynamic parameters were recorded upon completion of the animal preparation.

### 4.4. Induction of Unilateral Acute Lung Injury

Animals were placed in the left lateral position to facilitate targeted lavage of the left lung. The bronchial cuff was blocked. During ALI induction, the left lung was ventilated with a tidal volume of 3 mL/kg bw, a FiO_2_ of 1.0, and an RR of 10/minute, while the right lung received continuous positive airway pressure of 10 cmH_2_O with a FiO_2_ of 1.0 using a second ventilator (Primus^®^, Dräger Medical, Luebeck, Germany) to prevent non-ventilated lung injury [34]. Positioning of the DLtube was checked by bronchoscopy. Muscle relaxation was achieved by administering 1 mg/kg bw of rocuronium (B. Braun, Melsungen AG, Germany) intravenously.

The left lung was lavaged three times with 160 mL of 0.9% NaCl and 0.3% Triton^®^ X-100 (Merck KGaA, Darmstadt, Germany), with 80 mL administered to the upper lobe and 80 mL to the lower lobe under bronchoscopic guidance to ensure adequate distribution. A 5 min pause was taken between each lavage. During the procedure, peak inspiratory pressure was limited to 30 cmH_2_O. Noradrenaline was administered to maintain hemodynamic stability if mean arterial pressure dropped below 70 mmHg. Subsequently, the right lung was recruited with 30 cmH_2_O.

In two animals, only bronchoscopy without lavage was performed in the left lateral position, representing a sham procedure.

Once repositioned to the supine position, the pigs were ventilated again with 6 mL/kg bw through the DLtube using the previously described ventilation settings until randomization. The bronchial cuff remained blocked to prevent any lavage solution from entering the right lung. Correct DLtube position was checked post-lavage in supine position and after three hours.

### 4.5. Experimental Protocol

**Figure 11:**
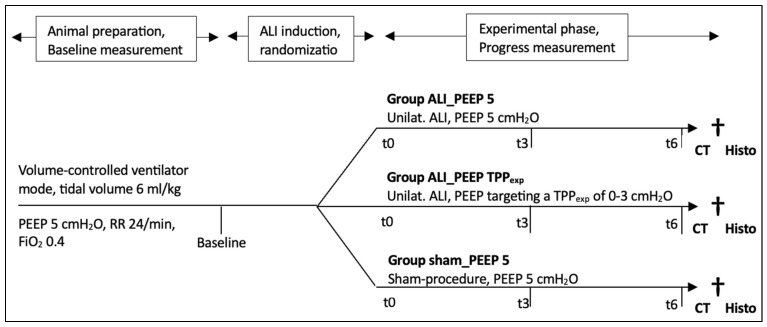
**Timeline of the experimental protocol.** Figure 11 illustrates the timeline of the experimental protocol. Baseline parameters were assessed following animal preparation, prior to ALI induction. Throughout the experimental phase, respiratory and hemodynamic parameters were recorded hourly. After the six-hour experimental phase, broncho-alveolar lavage fluid was collected and a CT scan was performed, followed by the euthanasia of the animals and the collection of histological samples. Abbreviations: ALI: acute lung injury; cmH_2_O: centimeter of water; CT: computed tomography; FiO_2_: inspiratory oxygen fraction; h: hours; Histo: histology; kg: kilogram; min: minutes; ml: milliliter; mmHg: millimeter mercury; PEEP: positive end-expiratory pressure; RR: respiratory rate; t: timepoint (t0 = 0 h, t3 = 3 h, t6 = 6 h after ALI induction); unilat.: unilateral; †: euthanasia.

Following ALI induction, the animals were randomized into three study groups using a simple lottery method with prepared slips of paper: ALI_PEEP 5, ALI_PEEP TPP_exp_, and sham_PEEP 5. Subsequently, ALI_PEEP 5 was ventilated with a fixed PEEP of 5 cmH_2_O, while ALI_PEEP TPP_exp_ received a PEEP adjusted (hourly) to achieve a TPP_exp_ between 0 and 3 cmH_2_O. Sham_PEEP 5, following the sham procedure, was ventilated similarly to ALI_PEEP 5 with a fixed PEEP of 5 cmH_2_O. All groups continued to be ventilated in a volume-controlled mode with a tidal volume of 6 mL/kg bw and an I:E ratio of 1:2. If necessary, adjustments of FiO_2_ and RR were made to maintain physiological values, as monitored by blood gas analysis. During the six-hour experimental phase, respiratory and hemodynamic parameters were recorded hourly (t1 to t6, corresponding to 1 to 6 h after ALI induction). At the end of the experimental phase (see Figure 11), plasma samples were taken and thoracic computed tomography was performed, followed by euthanasia and the collection of histological samples.

### 4.6. Measurements of Respiratory Parameters

Lung mechanics and hemodynamic parameters were recorded at baseline, after randomization (t0), and hourly (t1 to t6) throughout the six-hour experimental phase. Static measurements of airway and esophageal pressures were recorded during an expiratory and inspiratory hold maneuver.

Calculated measures were as follows:

*Peak inspiratory pressure = P_peak_ (cmH_2_O)*

*Positive end-expiratory pressure = PEEP (cmH_2_O)*

*Driving pressure (ΔP) = Plateau pressure (P_plat_) − PEEP (cmH_2_O)*

*Inspiratory esophageal pressure = P_ESinsp_ (cmH_2_O)*

*Expiratory esophageal pressure = P_ESexp_ (cmH_2_O)*

*End-expiratory transpulmonary pressure (TPP_exp_) = PEEP − P_ESexp_ (cmH_2_O)*

*End-inspiratory transpulmonary pressure (TPP_insp_) = P_plat_ − P_ESinsp_ (cmH_2_O)*

*Transpulmonary pressure (ΔP_L_) = TPP_insp_ − TPP_exp_ (cmH_2_O)*

*Static respiratory system compliance (C_RS_) = calculated by the ventilator (cmH_2_O/mL)*

*Mechanical power (MP) = 0.098 × RR × T_V_ × (P_peak_ − 0.5 × (P_plat_ − PEEP)) (J/min).*

*Partial arterial pressure of oxygen (paO_2_) = measured with arterial blood gas analysis*

*Partial arterial pressure of carbon dioxide (paCO_2_) = measured with arterial blood gas analysis*

*Lactate = measured with arterial blood gas analysis*

Heart rate = calculated by the PiCCO^®^ System *

Heart index (HI) = calculated by the PiCCO^®^ System *

Extravascular lung water index (ELWI) = calculated by the PiCCO^®^ System *

Global end-diastolic volume index (GEDI) = calculated by the PiCCO^®^ System *

* settings were configured according to recommendations for the porcine model [69].

### 4.7. High-Resolution Computed Tomography

At the end of the six-hour experimental phase, two consecutive non-enhanced chest high-resolution computed tomography (Somatom Force, Siemens Healthineers, Forchheim, Germany) scans were performed during an expiratory hold with a PEEP of 5 cmH_2_O and during an inspiratory hold with a P_plat_ of 30 cmH_2_O. The reconstructed slice thickness was 1.5 mm.

A modified HRCT scoring system, based on the scoring system by Ichikado et al., was applied by two readers in consensus, who were blinded to the study groups [50,71]. For each animal, multiple regions per lung side were scored according to the modified Ichikado system, and the resulting values were averaged to obtain a representative score per side for statistical comparison. Six hours after ALI induction (corresponding to the exudative phase of ALI), honeycombing patterns were not expected, as they typically occur in the fibrotic late phase. Consequently, the Ichikado score was modified by excluding grade 6 (*honeycombing*) from the analysis and combining grades 4 (*groundglass attenuation with traction bronchiolectasis or bronchiectasis*) and 5 (*consolidation with traction bronchiolectasis or bronchiectasis*) under the criterion of “*bronchodilatation*” [71,72].

### 4.8. Histological Analysis

Histopathological lung samples collected post mortem were immediately fixed in 4% formalin and later stained with hematoxylin and eosin. Three samples per site were obtained from the upper (1×) and lower (2×) lobes for histological evaluation. Two pathologists, blinded to the study variables, initially evaluated each sample using a standardized lung injury scoring system [20]. Twenty random high-power fields (400× total magnification) were evaluated per sample for LIS calculation.

To better reflect early ALI pathology, we applied a modified LIS for our final analysis, which excluded two variables—hyaline membranes and proteinaceous debris—since they do not typically appear in the early exudative phase of ALI [54]. The remaining variables were weighted to reflect their relevance in the histological assessment of ALI: (A) Neutrophils in the alveolar space per field, categorized as none (0 points), 1–5 (1 point), or >5 (2 points); (B) Neutrophils in the interstitial space per field, categorized as none (0 points), 1–5 (1 point), or >5 (2 points); and (C) Alveolar septal thickening, classified as <2× thickening (0 points), 2–4× thickening (1 point), or >4× thickening (2 points). The resulting modified LIS ranges from 0 (minimal damage) to 0.72 (severe damage) and was calculated as follows:

*Modified LIS = [(20 × A) + (14 × B) + (2 × C)]/(number of fields × 72).*

### 4.9. Plasma Biomarker Profiling

To explore systemic inflammatory and injury-related responses, we performed an exploratory protein biomarker analysis from EDTA plasma samples obtained at baseline and at the end of the experiment (t6). The analysis was conducted in collaboration with Sciomics GmbH (Heidelberg, Germany) using the scioCD discovery platform, a high-content antibody-based microarray assay enabling semi-quantitative profiling of ~120 cytokines, chemokines, and surface markers.

Plasma was obtained by centrifugation of whole blood (EDTA tubes, 2000g, 10 min, 4 °C), aliquoted, and stored at −80 °C until analysis. Firstly, the bulk protein concentration was determined. For the assay, 50 µL of plasma per sample were fluorescently labelled with scioDye 2 (Sciomics), while a pooled reference sample was labelled with scioDye 1. After two hours of labelling, the reaction was stopped and buffer exchanged to PBS. All samples were stored at −20 °C until hybridization. Samples were processed using a reference-based dual-color approach on scioCD antibody microarrays (Sciomics), with each antibody printed in quadruplicate. Arrays were blocked using scioBlock (Sciomics) and incubated for three hours with the labelled sample and reference mixture. Slides were subsequently washed, rinsed, and dried with nitrogen [73]. Microarray slides were scanned using a Powerscanner (Tecan Group Ltd., Männedorf, Switzerland) under constant laser and PMT settings. Spot segmentation was performed using GenePix Pro 6.0 (Molecular Devices, Union City, CA, USA). Functional annotation of the differentially expressed proteins was performed using STRING, which provides integrated enrichment analyses for KEGG pathways and Gene Ontology (GO) terms [74,75,76]. Enriched pathways and biological processes were identified based on a false discovery rate (FDR)-adjusted *p*-value < 0.05.

For further methodological and analytical details, please refer to the Supplementary Material.

### 4.10. Data Analysis

Sample size was calculated based on expected alterations in respiratory and histological parameters derived from data in previous studies [19,25,77] and unpublished findings from our lab. Statistical analyses were performed using SPSS (Version 27.0, IBM Corp., Armonk, NY, USA). A significance level of *p* < 0.05 was applied for all tests. Graphs were created using GraphPad (Version 10.3.1, GraphPad Software, San Diego, CA, USA) for data visualization.

For respiratory and hemodynamic parameters, one-way ANOVA with Bonferroni post hoc tests was applied to compare the study groups at baseline and t6, as normality and homogeneity of variances were confirmed.

For HRCT data analysis, the Wilcoxon signed-rank test was used to evaluate differences between expiratory and inspiratory holds within the same lung. Comparisons between the right and left lung within each group were performed using the Mann–Whitney U test, which was also applied to compare study groups under specific conditions (e.g., expiratory or inspiratory hold).

For the histopathological assessment of the modified lung injury score, one-way ANOVA with Bonferroni post hoc tests was used to compare the study groups separately for the right and left lung, as normality and homogeneity of variances were confirmed. Differences between the LIS of the right and left lung within the same animal were analyzed using the Wilcoxon signed-rank test.

For the blinded analysis of plasma protein biomarkers, signal intensities from the microarrays were background-corrected, log_2_-transformed, and normalized using a specialized invariant Lowess method. Differential protein abundance between baseline and t6 samples, as well as between experimental groups, was assessed using linear models for microarray data (LIMMA) implemented in the R/Bioconductor environment, following established criteria for antibody microarray analyses as previously published [78,79,80]. Log-fold changes (logFC) were calculated relative to the pooled reference. Proteins with |logFC| > 0.5 and adjusted *p*-value < 0.005 were considered differentially regulated, while those with 0.25 < |logFC| ≤ 0.5 were considered noteworthy. *p*-values were adjusted for multiple testing using the Benjamini–Hochberg method.

## 5. Conclusions

In this porcine model of unilateral acute lung injury, TPP-guided PEEP titration resulted in lower driving pressure and improved respiratory compliance compared to a fixed low PEEP strategy, indicating more homogeneous tidal volume distribution. Computed tomography revealed increased collateral damage in the initially non-injured lung under fixed low PEEP, suggesting that individualized PEEP settings may help limit regional overdistension in asymmetric injury. However, these physiological improvements did not translate into significant differences in histopathological lung injury scores. Complementing these findings, exploratory cytokine profiling revealed a coordinated systemic inflammatory response exclusively in the TPP-guided group, whereas cytokine levels remained stable under the fixed low PEEP strategy. These molecular results raise important questions about the relationship between ventilation mechanics and inflammatory signaling, and underscore the need for further studies to determine whether individualized PEEP strategies can influence the trajectory of systemic inflammation and lung recovery. Taken together, these findings support the use of TPP-guided PEEP to optimize mechanical conditions in heterogeneous lung injury, while highlighting the importance of integrating molecular readouts to assess potential long-term benefits and clinical applicability.

## Figures and Tables

**Table 1:** **t6 measurements.** Table 1 shows the mean values ± SEM of respiratory and hemodynamic parameters for the different groups at t6, along with the *p*-values from the one-way ANOVA.

Parameter	ALI_PEEP 5 Mean ± SEM	ALI_PEEP TPP_exp_ Mean ± SEM	sham_PEEP 5 Mean ± SEM	*p*-Value ANOVA
SpO_2_ (%)	99.2 ± 0.4	98.3 ± 0.7	99.0 ± 0.0	0.535
paO_2_ (mmHg)	152.4 ± 10.3	183.1 ± 7.6	186.9 ± 3.4	0.048
paCO_2_ (mmHg)	42.6 ± 1.0	50.1 ± 4.3	47.7 ± 5.2	0.267
Resp. rate (bpm)	25.3 ± 0.8	24.7 ± 0.7	22.0 ± 2.0	0.162
V_T_ (mL)	310 ± 10	300 ± 20	300 ± 40	0.915
P_peak_ (cmH_2_O)	30.3 ± 1.3	29.8 ± 1.0	20.5 ± 0.5	0.003
P_plat_ (cmH_2_O)	17.5 ± 0.6	19.8 ± 0.8	13.2 ± 1.7	0.003
PEEP (cmH_2_O)	5.0 ± 0.0	10.8 ± 0.9	5.0 ± 0.0	<0.001 *
ΔP (cmH_2_O)	12.3 ± 0.7	9.0 ± 0.6	8.0 ± 1.6	0.005 ^⨀^
Compliance (ml/cmH_2_O)	23.2 ± 2.3	31.8 ± 1.4	36.5 ± 2.5	0.006 ^#^
Resistance (cmH_2_O/L/s)	16.2 ± 0.9	19.2 ± 3.4	14.0 ± 0.0	0.540
Mech. Power (j/min)	18.6 ± 1.1	18.3 ± 0.7	10.8 ± 2.2	0.004
P_ESinsp_ (cmH_2_O)	13.3 ± 0.7	13.6 ± 1.4	7.7 ± 0.9	0.046
P_ESexp_ (cmH_2_O)	7.6 ± 0.8	9.6 ± 1.3	3.3 ± 1.3	0.037
TPP_exp_ (cmH_2_O)	−2.6 ± 0.8	1.2 ± 0.7	1.8 ± 1.3	0.008 ^✣^
ΔP_L_ (cmH_2_O)	6.7 ± 1.0	5.0 ± 0.8	3.8 ± 2.1	0.245
Heart rate (bpm)	70.5 ± 3.2	81.0 ± 5.7	65.0 ± 1	0.152
MAP (mmHg)	81.7 ± 3.2	75.0 ± 3.2	93.0 ± 0.0	0.037
Lactate (mg/dL)	10.7 ± 1.9	11.2 ± 2.3	7.6 ± 0.1	0.655
Cardiac index (L/min/m^2^)	3.6 ± 0.3	3.2 ± 0.3	3.1 ± 0.5	0.457
GEDI (mL/m^2^)	677.7 ± 36.5	537.5 ±34.8	677 ± 0.5	0.031 ^↡^
ELWI (mL/kg)	13.0 ± 1.4	12.3 ± 1.4	15.0 ± 1.0	0.615
CVP (mmHg)	12.3 ± 1.3	14.7 ± 1.5	12.5 ± 0.5	0.460

* post hoc Bonferroni (PEEP): ALI_PEEP 5 vs. ALI_PEEP TPP_exp_: *p* = <0.001; ^⨀^ post hoc Bonferroni (ΔP): ALI_PEEP 5 vs. ALI_PEEP TPP_exp_: *p* = 0.012; ^#^ post hoc Bonferroni (Compliance): ALI_PEEP 5 vs. ALI_PEEP TPP_exp_: *p* = 0.022; ^✣^ post hoc Bonferroni (TPP_exp_): ALI_PEEP 5 vs. ALI_PEEP TPP_exp_: *p* = 0.014; ^↡^ post hoc Bonferroni (GEDI): ALI_PEEP 5 vs. ALI_PEEP TPP_exp_: *p* = 0.042. Abbreviations: ALI: acute lung injury, ANOVA: analysis of variance, bpm: beats per minute, cmH_2_O: centimeters of water, CVP: central venous pressure, dL: deciliter, ELWI: extravascular lung water index, GEDI: global end-diastolic volume index, j: joule, kg: kilogram, L: liter, m^2^: square meter, MAP: mean arterial pressure, mg: milligram, min: minute, mL: milliliter, mmHg: millimeters of mercury, paCO_2_: partial pressure of carbon dioxide, paO_2_: partial pressure of oxygen, P_peak_: peak inspiratory pressure, P_plat_: plateau pressure, P_ESinsp_: inspiratory esophageal pressure, P_ESexp_: expiratory esophageal pressure, s: second, SEM: standard error of the mean, SpO_2_: peripheral capillary oxygen saturation, TPP_exp_: expiratory transpulmonary pressure, t6: after 6 h of mechanical ventilation, V_T_: tidal volume, ΔPL: driving pressure.

**Table 2:** **HRCT lung score after 6 h of experimental mechanical ventilation (t6).** Table 2 presents the mean values ± SEM of the HRCT lung score after six hours of experimental mechanical ventilation, with annotations indicating significant differences between expiratory and inspiratory hold maneuvers, lung sides, and between study groups.

Group	Lung Side	HRCT Score Expiratory Hold (5 cmH_2_O)	HRCT Score Inspiratory Hold (30 cmH_2_O)
ALI_PEEP 5	Left	262 ± 11	270 ± 35
ALI_PEEP 5	Right	142 ± 7 *	125 ± 4 *^†^
ALI_PEEP TPP_exp_	Left	260 ± 12	268 ± 15
ALI_PEEP TPP_exp_	Right	121 ± 6 *^‡^	116 ± 8 *^‡^

* significant result (*p* < 0.05) for left vs. right lung; ^†^ significant result (*p* < 0.05) for inspiratory vs. expiratory hold within the same group; ^‡^ significant result (*p* < 0.05) for right lung in ALI_PEEP 5 vs. right lung in ALI_PEEP TPP_exp_. Abbreviations: ALI: acute lung injury, cmH_2_O: centimeters of water, HRCT: high-resolution computed tomography, SEM: standard error of the mean, t6: timepoint six hours, vs.: versus.

**Table 3:** **Histopathological lung injury score after 6 h of experimental mechanical ventilation (t6).** Table 3 presents the mean values ± SEM of the modified lung injury score after six hours of mechanical ventilation, with annotations indicating significant differences between the left and right lung, as well as among the study groups.

	ALI_PEEP 5 Mean ± SEM	ALI_PEEP TPP_exp_ Mean ± SEM	sham_PEEP 5 Mean ± SEM	*p*-Value ANOVA
**left lung**	0.56 ± 0.04	0.54 ± 0.04	0.33 ± 0.03	0.009 *
**right lung**	0.30 ± 0.02	0.27 ± 0.02	0.32 ± 0.02	0.450
***p*-value (Wilcoxon)**	<0.001 ^†^	< 0.001 ^†^	0.433	

* significant post hoc test result (*p* < 0.05) for ALI_PEEP 5 and ALI_PEEP TPP_exp_ vs. sham_PEEP 5; ^†^ significant result (*p* < 0.05) for left vs. right lung within the same group. Abbreviations: ANOVA: analysis of the variance, SEM: standard error of the mean, t6: timepoint six hours, vs.: versus.

## Data Availability

The data of the cytokine analysis generated in this study are publicly available in the Mendeley repository at https://data.mendeley.com/preview/sbfpsgp42x?a=bfd0195c-deda-4d76-8ab0-d5f1ef030be2 (accessed on 6 October 2025). The full list of antibodies used in the scioCD array is provided in the Supplementary Study Report. Additional raw data (respiratory mechanics, histology, and CT imaging) are partially available from the corresponding author upon reasonable request, as these datasets are part of ongoing research projects.

## Supplementary Materials

The following supporting information can be downloaded at: https://www.mdpi.com/article/10.3390/ijms26209985/s1.

## Abbreviations

The following abbreviations are used in this manuscript:

ALI	Acute Lung Injury
ANOVA	analysis of the variance
ARDS	Acute Respiratory Distress Syndrome
ATS	American thoracic society
bw	body weight
C_RS_	respiratory system compliance
CT	computed tomography
DAD	diffuse alveolar damage
DLtube	double-lumen tube
ΔP	driving pressure
ΔP_L_	transpulmonary pressure
ELWI	extravascular lung water index
FiO_2_	inspiratory oxygen concentration
GEDI	global end-diastolic volume index
HI	heart index
HRCT	high-resolution computed tomography
I:E ratio	inspiratory-to-expiratory ratio
LIMMA	linear models for microarray data
LIS	lung injury score
logFC	log-fold change
MP	mechanical power
MV	mechanical ventilation
n	number
NVLI	non-ventilated lung injury
paCO_2_	partial pressure of carbon dioxide
paO_2_	partial pressure of oxygen
P_peak_	peak inspiratory pressure
PEEP	positive end-expiratory pressure
P_ES_	esophageal pressure
P_ESexp_	end-expiratory transpulmonary pressure
P_ESinsp_	end-inspiratory transpulmonary pressure
P_plat_	plateau pressure
RCT	randomized controlled trial
RR	respiratory rate
t6	timepoint 6 h of mechanical ventilation
TPP	transpulmonary pressure
TPP_exp_	end-expiratory transpulmonary pressure
TPP_insp_	end-inspiratory transpulmonary pressure
VILI	ventilator induced lung injury
vs.	versus
V_T_	tidal volume
