# Supplementary material for: Optimizing Positive End-Expiratory Pressure in Asymmetric Acute Lung Injury in a Porcine Model: The Role of Transpulmonary Pressure
Source: Int J Mol Sci. 2025 Oct 14;26(20):9985. doi: 10.3390/ijms26209985 (PMC12563547; doi:10.3390/ijms26209985)
Supplement: Supplementary file 1 [file ijms-26-09985-s001.zip › ijms-3813249-supplementary.pdf]

Supplementary material

Contents

A. Tabel S1: Baseline measurements
B. Figure S1: HRCT scans of the sham_PEEP 5 group.
C. Study Report: Exploratory Cytokine Profiling

A. Tabel S1: Baseline measurements

Table S1: Baseline measurements

Table S1 shows the mean values ± SEM of respiratory and hemodynamic parameters for the different groups at baseline, along with the p-values from the one-way ANOVA

**Parameter**	**Group A** mean ± SEM	**Group B** mean ± SEM	**Group C** mean ± SEM	**p-value** ANOVA
Body weight (kg)	51.3 ± 2.0	49.8 ± 2.8	50.5 ± 6.5	0.921
SpO_2 (%)	98.5 ± 0.5	98.0 ± 0.5	97.5 ± 0.5	0.530
paO_2 (mmHg)	170.1 ± 12.6	173.4 ± 9.9	209.8 ± 2.3	0.211
$paCO_2$ (mmHg)	50.2 ± 2.7	48.4 ± 1.4	46.0 ± 1.0	0.580
Respiratory rate (bpm)	24.0 ± 0.0	24.0 ± 0.0	24.0 ± 0.0	0.927
V_T (ml)	310 ± 10	310 ± 20	300 ± 40	0.945
P_{peak} (cmH_2O)	18.5 ± 0.3	18.7 ± 0.7	18.0 ± 0,0	0.826
P_{plat} (cmH_2O)	12.5 ±0.5	11.5 ± 0.4	11.0 ± 0.2	0.169
PEEP (cmH_2O)	5.0 ± 0.0	5.0 ± 0.0	5.0 ± 0.0	-
ΔP (cmH_2O)	7.4 ± 0.5	6.2 ± 0.4	5.7 ± 0.2	0.113
Compliance (ml/cmH_2O)	40.7 ± 4.3	46.7 ± 2.2	49.5 ± 5.5	0.339
Resistance (cmH_2O/l/sec)	12.2 ± 0.8	13.3 ± 0.2	13.5 ± 0.5	0.301
Mechanical Power (j/min)	10.6 ± 0.4	11.2 ± 0.9	10.7 ± 1.4	0.895
P_{ESinsp} (cmH_2O)	11.9 ± 1.0	11.1 ± 1.2	9.3 ± 1.7	0.523
P_{ESexp} (cmH_2O)	7.5 ± 0.9	7.1 ± 1.1	5.5 ± 2.6	0.631
TPP_{exp} (cmH_2O)	-2.5 ± 0.9	-2.1 ± 1.1	-0.5 ± 2.6	0.631
ΔP_L (cmH_2O)	6.5 ± 0.6	5.1 ± 0.9	2.7 ± 2.3	0.108
Heart rate (bpm)	76.0 ± 5.7	74.5 ± 6.2	84.5 ± 5.5	0.688
MAP (mmHg)	81.0 ± 5.3	78.7 ± 5.0	76.5 ± 10.5	0.899
Lactate (mg/dl)	18.6 ± 6.8	7.4 ± 1.0	11.7 ± 5.1	0.286
Cardiac index (l/min/m^2)	5.0 ± 0.6	4.2 ± 0.2	4.2 ± 0.2	0.327
GEDI (ml/m^2)	782 ± 67.1	635 ± 26.9	589 ± 52.5	0.089
ELWI (ml/kg)	13.3 ± 1.4	14.7 ± 1.5	13.5 ± 0.5	0.781
CVP (mmHg)	13.8 ± 0.8	12.5 ± 0.9	13.5 ± 2.5	0.567

Abbreviations: ANOVA: analysis of variance, bpm: beats per minute, cmH_2O: centimeters of water, CVP: central venous pressure, dl: deciliter, ELWI: extravascular lung water index, GEDI: global end-diastolic volume index, j: joule, kg: kilogram, l: liter, m^2: square meter, MAP: mean arterial pressure, mg: milligram, min: minute, ml: milliliter, mmHg: millimeters of mercury, $paCO_2$: partial pressure of carbon dioxide, paO_2: partial pressure of oxygen, P_{peak}: peak inspiratory pressure, P_{plat}: plateau pressure, P_{ESinsp}: inspiratory esophageal pressure, P_{ESexp}: expiratory esophageal pressure, sec: second, SEM: standard error of the mean, SpO_2: peripheral capillary oxygen saturation, TPP_{exp}: expiratory transpulmonary pressure, V_T: tidal volume, ΔPL: driving pressure

B. Figure S1: HRCT scans of the sham_PEEP 5 group

Figure S1: HRCT scans of the sham_PEEP 5 group.

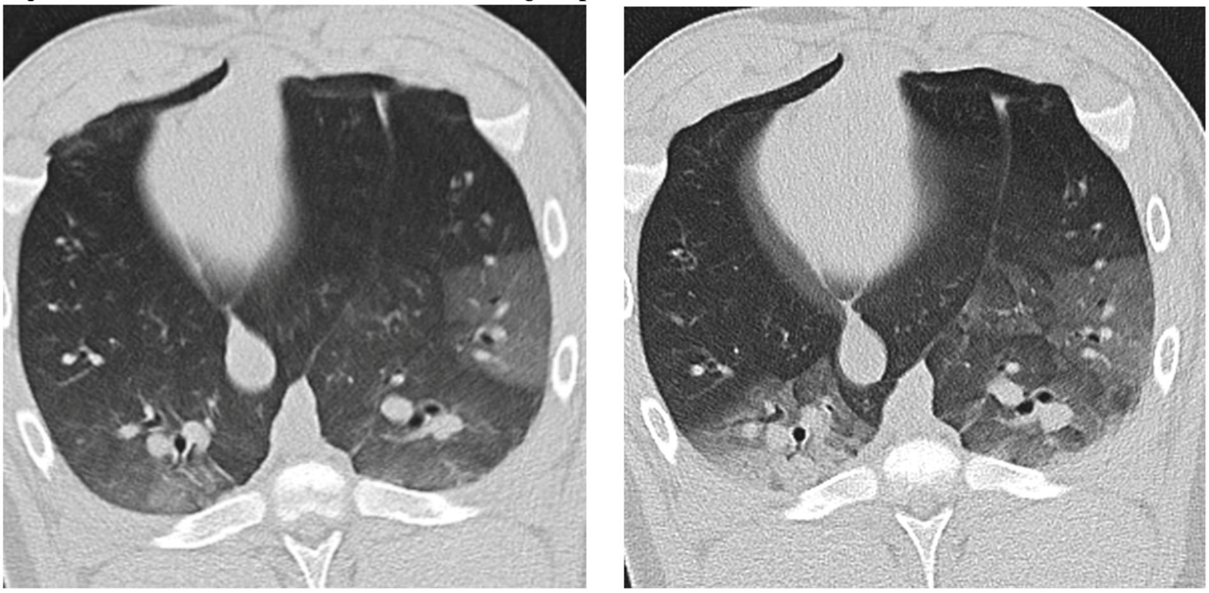

Representative high-resolution computed tomography (HRCT) scans of the successfully scanned pig from the sham_PEEP 5 group after six hours of mechanical ventilation (t6). The left panel shows an expiratory hold maneuver at PEEP 5 cmH_2O, and the right panel depicts an inspiratory hold maneuver at Pplat of 30 cmH_2O.

C. Study Report: Exploratory Cytokine Profiling

Study Report Contents

1. Aim of the Experiment
2. Material & Methods
    2.1 Samples and protein extraction
    2.2 Sample labelling
    2.3 Sample incubation
    2.4 Data acquisation and analysis
3. Results
    3.1 Cluster analysis
        3.1.1 Hierarchical cluster analysis
        3.1.2 Principal component analysis
    3.2 Differentially abundant proteins
        3.2.1 PEEP 5: Timepoint 6h vs Baseline
        3.2.2 TPPexp: Timepoint 6h vs Baseline
        3.2.3 Sham: Timepoint 6h vs Baseline
        3.2.4 Timepoint 6h: PEEP 5 vs TPPexp
        3.2.5 Timepoint 6h: PEEP 5 vs Sham
        3.2.6 Timepoint 6h: TPPexp vs Sham
        3.2.7 PEEP 5 Timepoint 6h vs Baseline all
        3.2.8 TPPexp Timepoint 6h vs Baseline all
        3.2.9 Sham Timepoint 6h vs Baseline all
    3.3 Individual protein levels
    3.4 Heatmap of differential proteins
    3.5 Functional annotation of differential proteins
        3.5.1 Differential and noteworthy proteins in TPPexp Timepoint 6h vs Baseline all
    3.6 Protein interactions
        3.6.1 Protein interactions in TPPexp Timepoint 6h vs Baseline all
4. References

1 Aim of the Experiment

The aim of the experiment was to identify protein differences in pig plasma samples.

2 Material & Methods

2.1 Samples and protein extraction

26 plasma samples were provided by the customer. The bulk protein concentration was determined by BCA assay (Table S2). A reference sample was established by pooling an identical volume of each sample.

Sample	Customer Sample ID	Sample Group	Conc. [mg/ml]
KN001	16/1	PEEP5 Baseline	66.30
KN002	16/2	PEEP5 Timepoint 6h	60.00
KN003	1/1	PEEP5 Baseline	58.10
KN004	2/1	PEEP5 Baseline	72.10
KN005	3/1	PEEP5 Baseline	53.10
KN006	3/2	PEEP5 Timepoint 6h	49.60
KN007	6/1	PEEP5 Baseline	46.20
KN008	6/2	PEEP5 Timepoint 6h	54.60
KN009	11/1	PEEP5 Baseline	58.00
KN010	11/2	PEEP5 Timepoint 6h	48.60
KN011	4/1	TPPexp Baseline	53.40
KN012	4/2	TPPexp Timepoint 6h	49.10
KN013	9/1	TPPexp Baseline	49.20
KN014	9/2	TPPexp Timepoint 6h	38.50
KN015	10/1	TPPexp Baseline	51.30
KN016	10/2	TPPexp Timepoint 6h	35.40
KN017	13/1	TPPexp Baseline	61.90
KN018	13/2	TPPexp Timepoint 6h	34.90
KN019	14/1	TPPexp Baseline	58.90
KN020	14/2	TPPexp Timepoint 6h	52.50
KN021	15/1	TPPexp Baseline	53.80
KN022	15/2	TPPexp Timepoint 6h	52.20
KN023	5/1	Sham Baseline	55.10
KN024	5/2	Sham Timepoint 6h	54.70
KN025	8/1	Sham Baseline	52.80
KN026	8/2	Sham Timepoint 6h	50.40

Table S2: Overview of samples sent for analysis and measured protein concentrations.

2.2 Sample labelling

The samples were labelled at an adjusted protein concentration for two hours with scioDye 2 (Sciomics). The reference sample was labelled with scioDye 1 (Sciomics). After two hours the reaction was stopped and the buffer exchanged to PBS. All labelled protein samples were stored at -20° C until use.

2.3 Sample incubation

The 26 samples were analysed in a dual-colour approach using a reference based design on 26 scioCD antibody microarrays (Sciomics) targeting different CD surface markers and cytokines/chemokines. Each antibody is represented on the array in four replicates. The arrays were blocked with scioBlock (Sciomics) on a Hybstation 4800 (Tecan, Austria) and afterwards the samples were incubated competitively with the reference sample using a dual-colour approach. After incubation for three hours, the slides were thoroughly washed with 1x PBSTT, rinsed with 0.1x PBS as well as with water and subsequently dried with nitrogen [73].

2.4 Data acquisition and analysis

Slide scanning was conducted using a Powerscanner (Tecan, Austria) with constant instrument laser power and PMT settings. Spot segmentation was performed with GenePix Pro 6.0 (Molecular Devices, Union City, CA, USA). Acquired raw data were analysed using the linear models for microarray data (LIMMA) package of R-Bioconductor after uploading the median signal intensities [80]. For normalisation, a specialised invariant Lowess method was applied [79].

For analysis of the samples, a one-factorial linear model was fitted via least squares regression with LIMMA, resulting in a two-sided t-test or F-test based on moderated statistics. All presented p values were adjusted for multiple testing by controlling the false discovery rate according to Benjamini and Hochberg.

Differences in protein abundance between different samples or sample groups are presented as log-fold changes (logFC) calculated for the basis 2. In a study comparing samples versus control a logFC= 1 means that the sample group had on average a 21 = 2 fold higher signal than the control group. logFC=–1 stands for 2−1 = 1/2 of the signal in the sample as compared to the control group.

Proteins with a |logFC|> 0.5 and an adjusted p value < 0.05 were defined as differential and displayed in blue in the following volcano plots. Proteins reaching reduced thresholds as defined individually for each comparison, are defined as noteworthy and displayed in green. Please note that comparisons 3.2.3, 3.2.5, 3.2.6 and 3.2.9 compare sample groups with less than three samples. We advise caution when interpreting the results, especially the adjusted p values, of the differential analysis of those comparisons, due to their low statistical power.

3 Results

3.1 Cluster analysis

3.1.1 Hierarchical cluster analysis

The cluster analysis for the protein signal data of the complete data set (Figure S2) was performed.
A further cluster analysis was performed with only the differential data set (Figure S3).

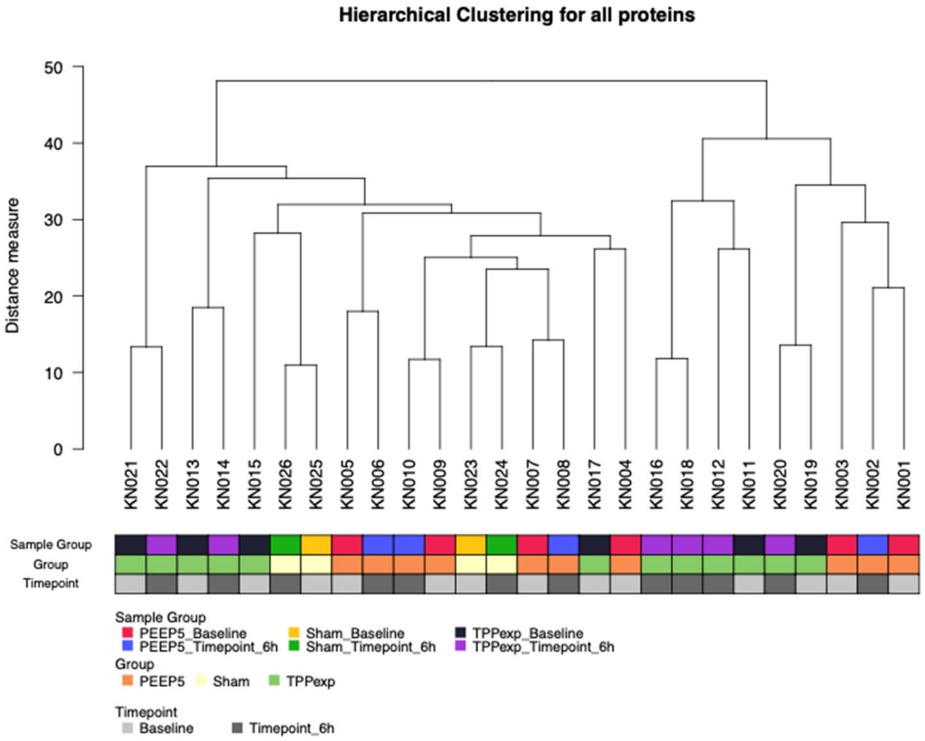

Figure S2: Hierarchical clustering of the protein extracts using complete array data. After averaging the four technical replicates of each antibody feature, the data were centered and scaled per antibody.

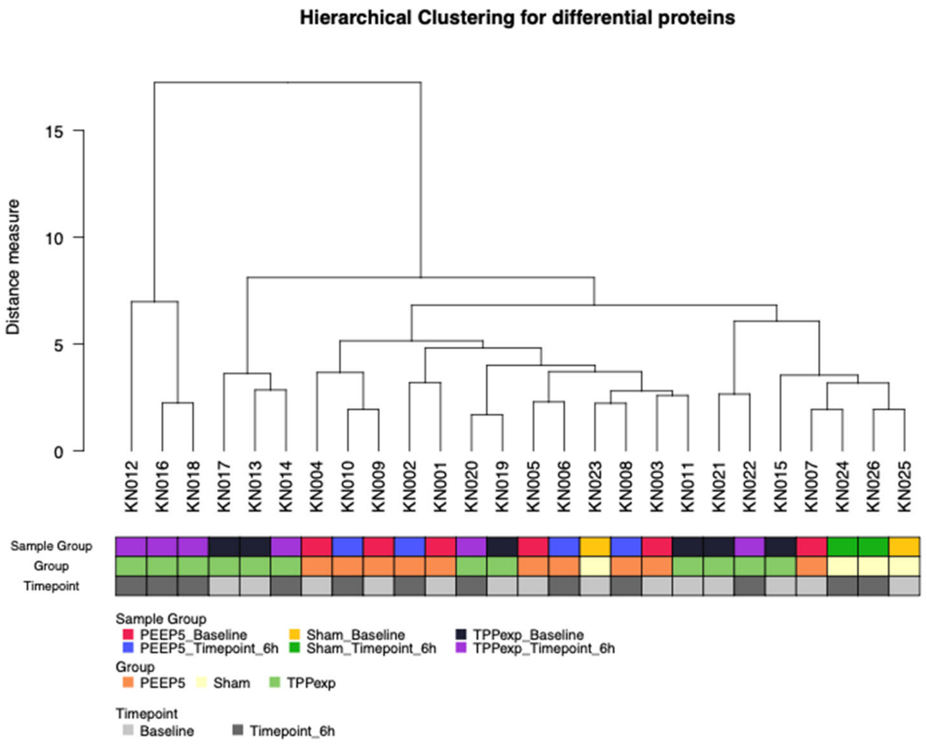

Figure S3: Hierarchical clustering of the protein extracts based on differential proteins. After averaging the four technical replicates of each antibody feature, the data were centered and scaled per antibody.

3.1.2 Principal component analysis

In addition, a principal component analysis (PCA) was performed for the array data (Figure S4) as well as for the array data filtered for differential proteins (Figure S5). In this plot, the location of the samples is defined by their first two principal components, i.e. linear combinations of protein features with the largest variance across the samples. Samples with a similar profile are located in close proximity.

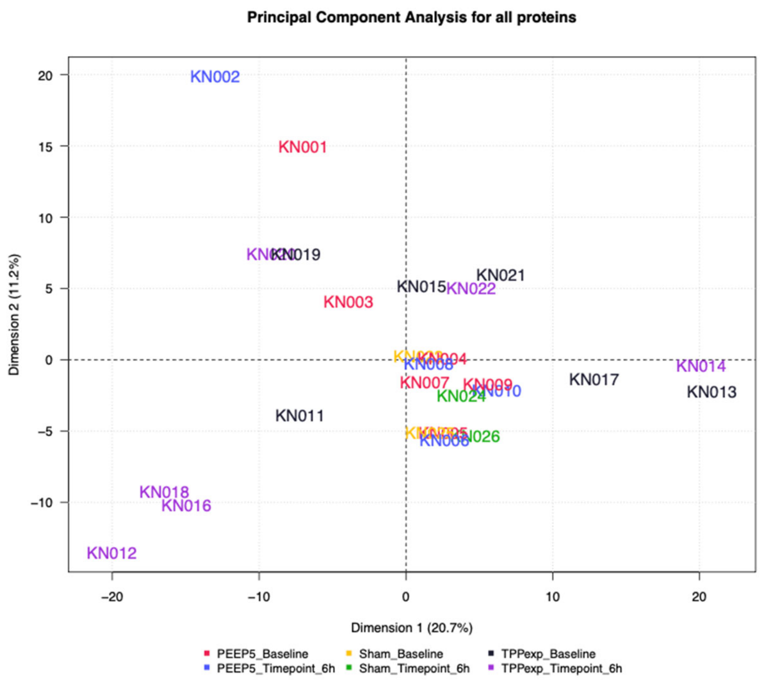

Figure S4: Scatter plot displaying the first two principal components of the samples' protein signal data using complete array data. The percentages given in the axis labels describe the ratio of total variance explained by the respective principal component.

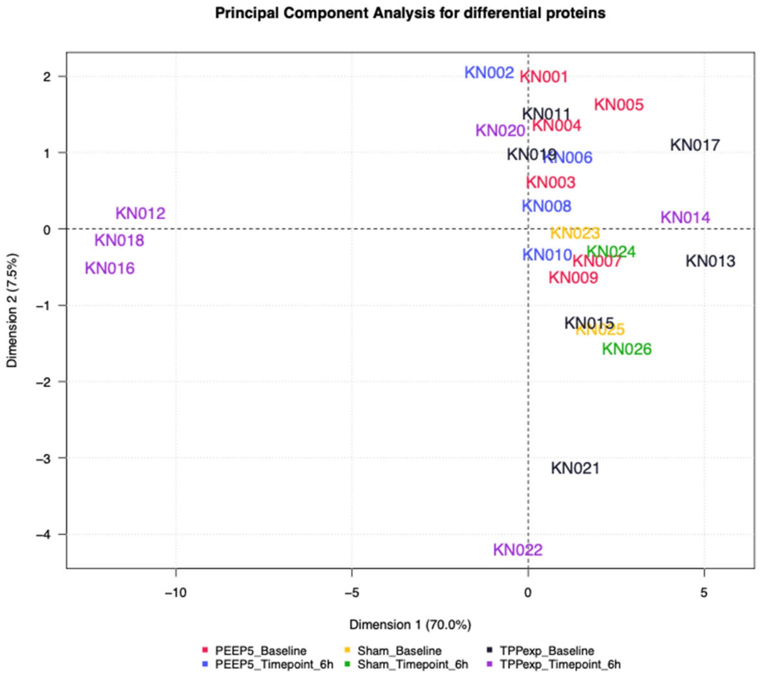

Figure S5: Scatter plot displaying the first two principal components of the samples' protein signal data based on differentially abundant proteins. The percentages given in the axis labels describe the ratio of total variance explained by the respective principal component.

3.2 Differentially abundant proteins

3.2.1 PEEP 5: Timepoint 6h vs Baseline

Between peep 5 timepoint 6h samples and peep 5 baseline samples, no antibodies recorded a differential protein abundance. The results of the statistical analysis are summarized in the volcano plot (Figure S6).

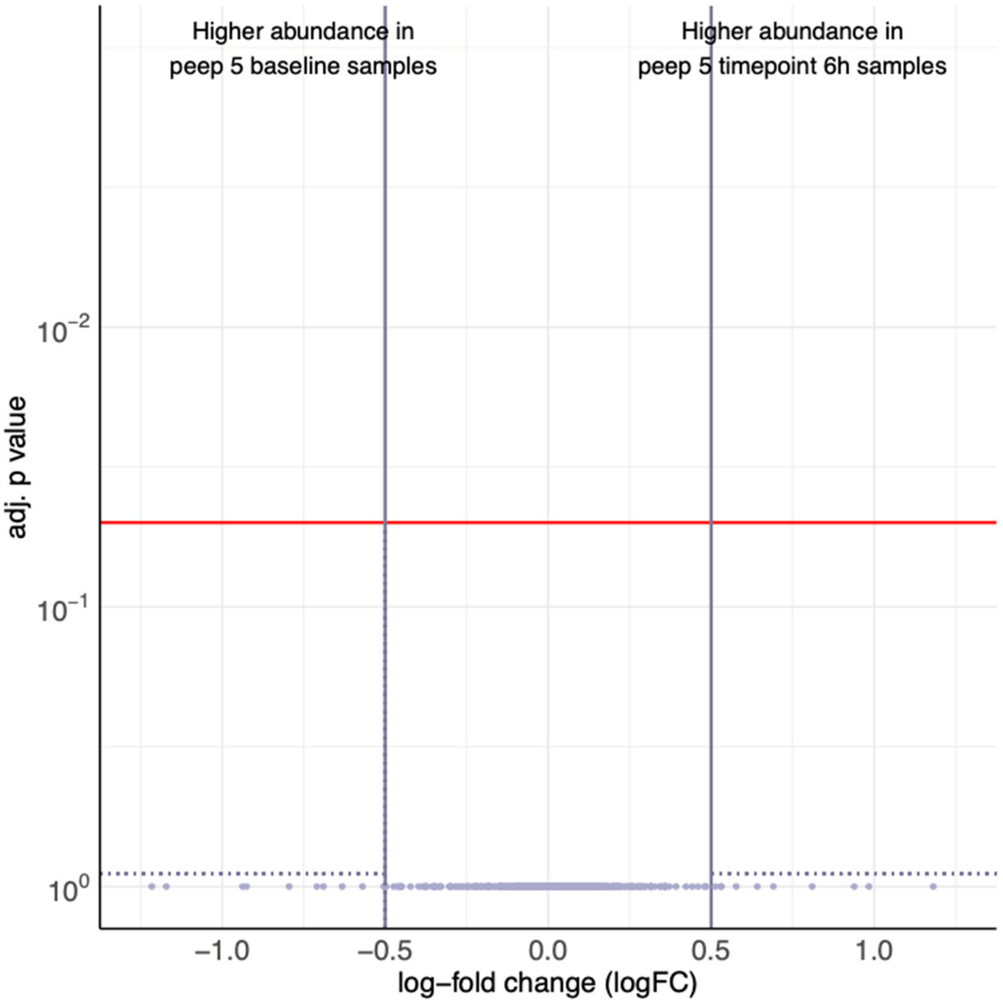

Figure S6: Volcano plot visualizing the differences in abundance between peep 5 timepoint 6h samples and peep 5 baseline samples as log-fold changes (logFC) and their corresponding p values (adj. for multiple testing). The red line indicates a significance level of adj. p value = 0.05, vertical lines indicate the logFC cutoffs of ±0.5. A positive logFC indicates higher abundance in peep 5 timepoint 6h samples, a negative logFC in peep 5 baseline samples. Differential proteins (|logFC| > 0.5, adj. p value < 0.05) are displayed with blue names. Non-significant proteins (adj. p value < 0.9) with a |logFC| > 0.5 are defined as noteworthy and displayed with green names.

3.2.2 TPPexp: Timepoint 6h vs Baseline

Between TPPexp timepoint 6h samples and TPPexp baseline samples, 22 antibodies recorded a differential protein abundance. The results of the statistical analysis are summarized in the volcano plot (Figure S7) and listed in Table S3. Furthermore, Table S4 lists noteworthy proteins reaching reduced logFC and significance thresholds.

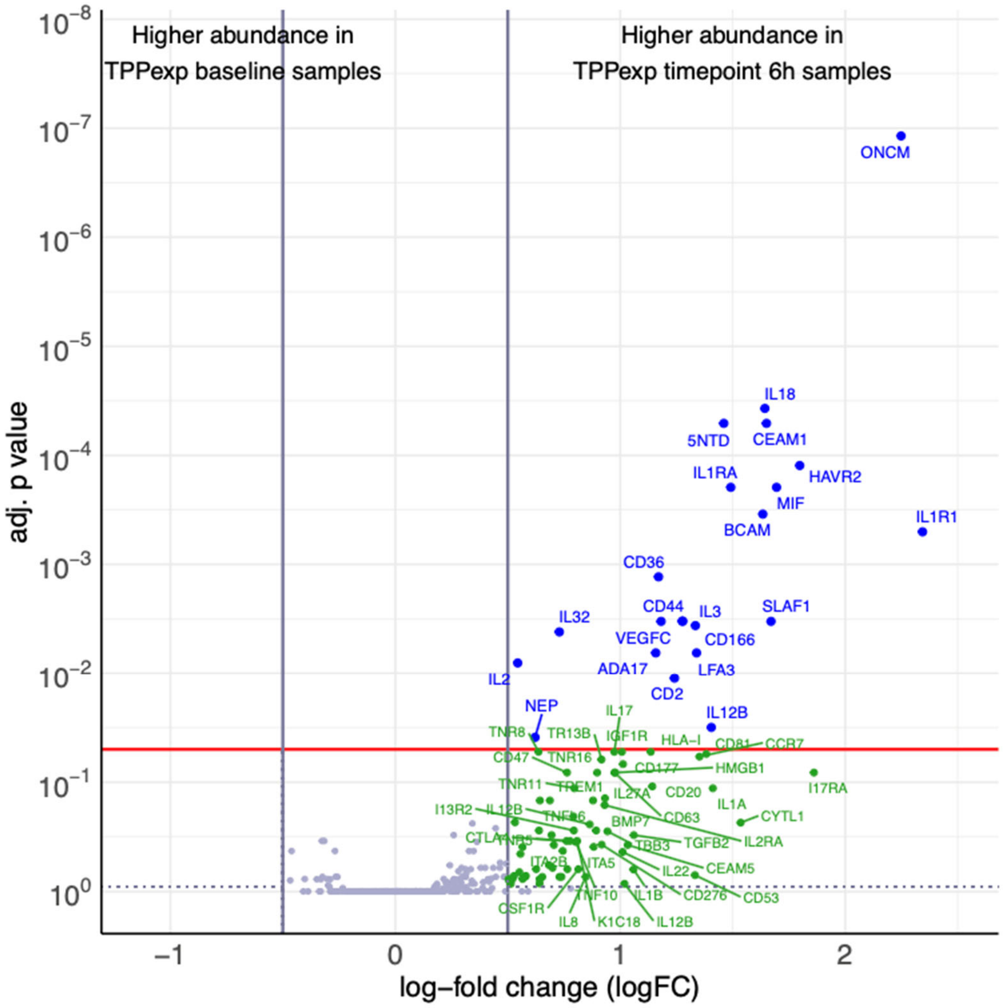

Figure S7: Volcano plot visualizing the differences in abundance between TPPexp timepoint 6h samples and TPPexp baseline samples as log-fold changes (logFC) and their corresponding p values (adj. for multiple testing). The red line indicates a significance level of adj. p value = 0.05, vertical lines indicate the logFC cutoffs of ±0.5. A positive logFC indicates higher abundance in TPPexp timepoint 6h samples, a negative logFC in TPPexp baseline samples. Differential proteins (|logFC| > 0.5, adj. p value < 0.05) are displayed with blue names. Non-significant proteins (adj. p value < 0.9) with a |logFC| > 0.5 are defined as noteworthy and displayed with green names.

Protein	AntibodyID	UniprotName	Uniprot-Entry	HGNC	logFC	AveExp	adj.p val
IL1R1	ab2100	IL1R1_HUMAN	P14778	IL1R1	2.34	10.21	5.0e-04
ONCM	ab1885	ONCM_HUMAN	P13725	OSM	2.25	11.16	1.2e-07
HAVR2	ab2067	HAVR2_HUMAN	Q8TDQ0	HAVCR2	1.80	10.31	1.2e-04
MIF	ab1816	MIF_HUMAN	P14174	MIF	1.69	9.64	2.0e-04
SLAF1	ab2132	SLAF1_HUMAN	Q13291	SLAMF1	1.67	9.40	3.3e-03
CEAM1	ab2363	CEAM1_HUMAN	P13688	CEACAM1	1.65	10.32	5.1e-05
IL18	ab1734	IL18_HUMAN	Q14116	IL18	1.64	8.49	3.7e-05
BCAM	ab2290	BCAM_HUMAN	P50895	BCAM	1.63	12.59	3.5e-04
IL1RA	ab2434	IL1RA_HUMAN	P18510	IL1RN	1.49	12.46	2.0e-04
5NTD	ab3694	5NTD_HUMAN	P21589	NT5E	1.46	14.15	5.1e-05
IL12B	ab1984	IL12B_HUMAN	P29460	IL12B	1.40	11.23	3.2e-02
LFA3	ab1462	LFA3_HUMAN	P19256	CD58	1.34	10.98	6.5e-03
CD166	ab2246	CD166_HUMAN	Q13740	ALCAM	1.33	9.64	3.6e-03
IL3	ab2172	IL3_HUMAN	P08700	IL3	1.28	8.30	3.3e-03
CD44	ab1540	CD44_HUMAN	P16070	CD44	1.28	13.46	3.3e-03
CD2	ab1356	CD2_HUMAN	P06729	CD2	1.24	9.48	1.1e-02
VEGFC	ab1178	VEGFC_HUMAN	P49767	VEGFC	1.18	9.24	3.3e-03
CD36	ab2783	CD36_HUMAN	P16671	CD36	1.17	11.68	1.3e-03
ADA17	ab2342	ADA17_HUMAN	P78536	ADAM17	1.16	11.76	6.5e-03
IL32	ab2749	IL32_HUMAN	P24001	IL32	0.73	9.62	4.2e-03
NEP	ab2254	NEP_HUMAN	P08473	MME	0.62	9.38	3.9e-02
IL2	ab1587	IL2_HUMAN	P60568	IL2	0.54	8.58	8.0e-03

Table S3: Proteins with differential abundance in TPPexp timepoint 6h samples and TPPexp baseline samples. Proteins with a positive logFC value had a higher abundance in TPPexp timepoint 6h samples, proteins with a negative value in TPPexp baseline samples. In addition, p values adjusted for multiple testing are listed. The Uniprot-Identifier links to the Uniprot-Entry [21].

Protein	AntibodyID	UniprotName	Uniprot-Entry	HGNC	logFC	AveExp	adj.p val
I17RA	ab2407	I17RA_HUMAN	Q96F46	IL17RA	1.86	11.12	8.2e-02
CYTL1	ab1670	CYTL1_HUMAN	Q9NRR1	CYTL1	1.54	10.06	2.3e-01
IL1A	ab2263	IL1A_HUMAN	P01583	IL1A	1.41	12.82	1.1e-01
CD81	ab3695	CD81_HUMAN	P60033	CD81	1.38	11.75	5.5e-02
CCR7	ab0987	CCR7_HUMAN	P32248	CCR7	1.35	11.29	5.8e-02
CD53	ab1544	CD53_HUMAN	P19397	CD53	1.33	9.91	7.1e-01
CD20	ab1594	CD20_HUMAN	P11836	MS4A1	1.14	10.47	1.1e-01
HLA-I	ab1553				1.14	13.41	5.3e-02
TGFB2	ab1753	TGFB2_HUMAN	P61812	TGFB2	1.06	10.26	3.0e-01
IL1B	ab1686	IL1B_HUMAN	P01584	IL1B	1.06	10.74	6.3e-01
CEAM5	ab2720	CEAM5_HUMAN	P06731	CEACAM5	1.03	10.56	3.7e-01
IL12B	ab1666	IL12B_HUMAN	P29460	IL12B	1.02	10.47	8.5e-01
CD177	ab1489	CD177_HUMAN	Q8N6Q3	CD177	1.01	8.80	6.8e-02
IL22	ab1933	IL22_HUMAN	Q9GZX6	IL22	1.01	8.98	4.3e-01
IGF1R	ab1995	IGF1R_HUMAN	P08069	IGF1R	1.01	9.24	5.3e-02
HMGB1	ab1215	HMGB1_HUMAN	P09429	HMGB1	0.98	13.03	8.2e-02
CD63	ab3703	CD63_HUMAN	P08962	CD63	0.97	12.95	8.2e-02
IL17	ab2717	IL17_HUMAN	Q16552	IL17A	0.97	11.78	5.3e-02
TBB3	ab1582	TBB3_HUMAN	Q13509	TUBB3	0.94	12.14	2.8e-01
IL27A	ab1741	IL27A_HUMAN	Q8NEV9	IL27	0.93	9.64	1.4e-01
IL2RA	ab1831	IL2RA_HUMAN	P01589	IL2RA	0.93	11.81	1.6e-01
CD276	ab2437	CD276_HUMAN	Q5ZPR3	CD276	0.92	12.49	3.7e-01
TR13B	ab2250	TR13B_HUMAN	O14836	TNFRSF13B	0.92	10.07	6.2e-02
TNR16	ab2445	TNR16_HUMAN	P08138	NGFR	0.90	12.51	8.2e-02
BMP7	ab1772	BMP7_HUMAN	P18075	BMP7	0.89	10.87	2.8e-01
ITA5	ab2299	ITA5_HUMAN	P08648	ITGA5	0.88	12.24	3.9e-01
TREM1	ab2125	TREM1_HUMAN	Q9NP99	TREM1	0.88	9.21	1.5e-01

Continued on next page

Protein	AntibodyID	UniprotName	Uniprot-Entry	HGNC	logFC	AveExp	adj.p val
IL12B	ab1731	IL12B_HUMAN	P29460	IL12B	0.86	13.24	2.4e-01
IL8	ab2312	IL8_HUMAN	P10145	CXCL8	0.85	9.13	7.3e-01
CSF1R	ab1925	CSF1R_HUMAN	P07333	CSF1R	0.81	12.73	6.2e-01
K1C18	ab3671	K1C18_HUMAN	P05783	KRT18	0.81	10.93	3.4e-01
TNF10	ab2073	TNF10_HUMAN	P50591	TNFSF10	0.80	9.85	3.5e-01
TNR11	ab1857	TNR11_HUMAN	Q9Y6Q6	TNFRSF11A	0.80	9.09	1.1e-01
I13R2	ab1947	I13R2_HUMAN	Q14627	IL13RA2	0.79	13.09	2.8e-01
TNFL6	ab1983	TNFL6_HUMAN	P48023	FASLG	0.79	9.43	2.1e-01
TNR5	ab1752	TNR5_HUMAN	P25942	CD40	0.78	9.52	3.4e-01
ITA2B	ab1432	ITA2B_HUMAN	P08514	ITGA2B	0.76	9.97	6.2e-01
CD47	ab1448	CD47_HUMAN	Q08722	CD47	0.76	13.90	8.2e-02
CTLA4	ab1117	CTLA4_HUMAN	P16410	CTLA4	0.76	12.50	3.4e-01
GP1BA	ab1433	GP1BA_HUMAN	P07359	GP1BA	0.74	10.30	4.2e-01
IL17C	ab2403	IL17C_HUMAN	Q9P0M4	IL17C	0.74	11.70	7.3e-01
TNR6	ab1478	TNR6_HUMAN	P25445	FAS	0.73	13.10	7.3e-01
LIF	ab2318	LIF_HUMAN	P15018	LIF	0.70	8.44	3.7e-01
IL8	ab1645	IL8_HUMAN	P10145	CXCL8	0.70	9.57	6.1e-01
CD8A	ab1376	CD8A_HUMAN	P01732	CD8A	0.69	9.47	3.0e-01
TNR9	ab2274	TNR9_HUMAN	Q07011	TNFRSF9	0.69	9.49	1.5e-01
TFR1	ab1471	TFR1_HUMAN	P02786	TFRC	0.68	11.32	5.7e-01
TNF14	ab2301	TNF14_HUMAN	O43557	TNFSF14	0.65	10.00	7.3e-01
IL23A	ab2949	IL23A_HUMAN	Q9NPF7	IL23A	0.64	9.17	1.5e-01
TNFA	ab2019	TNFA_HUMAN	P01375	TNF	0.64	8.37	7.7e-01
HAVR1	ab2265	HAVR1_HUMAN	Q96D42	HAVCR1	0.64	8.44	8.4e-01
CADH2	ab2760	CADH2_HUMAN	P19022	CDH2	0.64	10.49	2.8e-01
TNR8	ab1423	TNR8_HUMAN	P28908	TNFRSF8	0.64	9.16	5.3e-02
ENTP1	ab1111	ENTP1_HUMAN	P49961	ENTPD1	0.63	9.52	6.2e-01
GLPB	ab1492	GLPB_HUMAN	P06028	GYPB	0.58	10.25	7.2e-01
CD99	ab1481	CD99_HUMAN	P14209	CD99	0.58	11.97	7.3e-01
IL15	ab2444	IL15_HUMAN	P40933	IL15	0.57	11.04	7.7e-01
BTLA	ab3663	BTLA_HUMAN	Q7Z6A9	BTLA	0.57	13.65	3.9e-01
TLR2	ab1868	TLR2_HUMAN	O60603	TLR2	0.56	9.09	4.5e-01
LYAM2	ab1999	LYAM2_HUMAN	P16581	SELE	0.55	10.08	6.7e-01
IL34	ab2441	IL34_HUMAN	Q6ZMJ4	IL34	0.53	14.44	2.3e-01
IL20	ab1817	IL20_HUMAN	Q9NYY1	IL20	0.53	11.19	7.2e-01
ITA2B (CD41a)	ab1538		P08514	ITGA2B	0.52	10.03	7.7e-01
I13R1	ab1970	I13R1_HUMAN	P78552	IL13RA1	0.51	11.23	8.5e-01
CD28	ab1420	CD28_HUMAN	P10747	CD28	0.51	10.81	7.9e-01
AMPN	ab1390	AMPN_HUMAN	P15144	ANPEP	0.50	12.16	7.7e-01

Table S4: Proteins with noteworthy abundance in TPPexp timepoint 6h samples and TPPexp baseline samples, which feature notable logFCs, while not reaching the significance and logFC thresholds simultaneously. Proteins with a positive logFC value had a higher abundance in TPPexp timepoint 6h samples, proteins with a negative value in TPPexp baseline samples. In addition, p values adjusted for multiple testing are listed. The Uniprot-Identifier links to the Uniprot-Entry [21].

3.2.3 Sham: Timepoint 6h vs Baseline

Between sham timepoint 6h samples and sham baseline samples, no antibodies recorded a differential protein abundance. The results of the statistical analysis are summarized in the volcano plot (Figure S8).

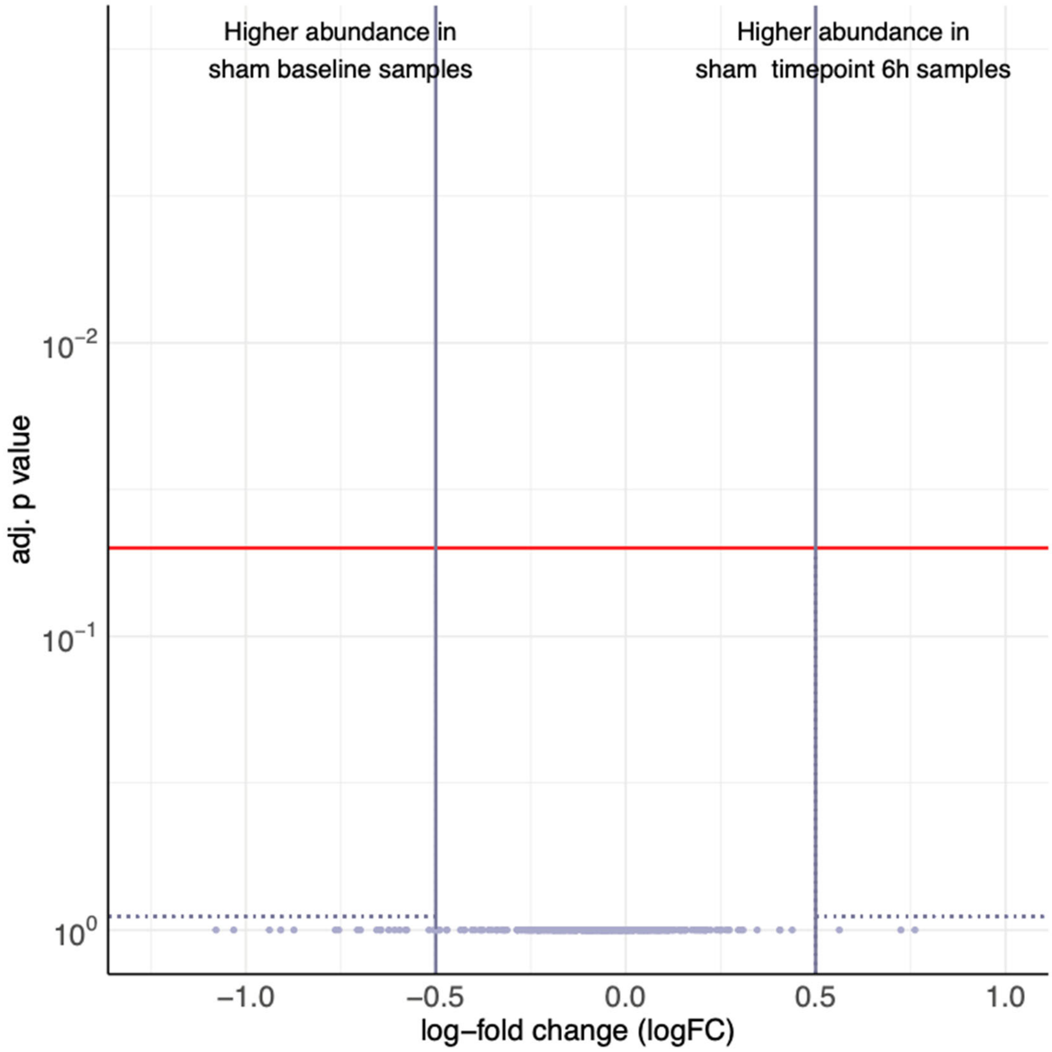

Figure S8: Volcano plot visualizing the differences in abundance between sham timepoint 6h samples and sham baseline samples as log-fold changes (logFC) and their corresponding p values (adj. for multiple testing). The red line indicates a significance level of adj. p value = 0.05, vertical lines indicate the logFC cutoffs of ±0.5. A positive logFC indicates higher abundance in sham timepoint 6h samples, a negative logFC in sham baseline samples. Differential proteins ($|logFC| > 0.5$, adj. p value < 0.05) are displayed with blue names. Non-significant proteins (adj. p value < 0.9) with a $|logFC| > 0.5$ are defined as noteworthy and displayed with green names.

3.2.4 Timepoint 6h: PEEP 5 vs TPPexp

Between peep 5 timepoint 6h samples and TPPexp timepoint 6h samples, 5 antibodies recorded a differential protein abundance. The results of the statistical analysis are summarized in the volcano plot (Figure S9) and listed in Table S5. Furthermore, Table S6 lists noteworthy proteins reaching reduced logFC and significance thresholds.

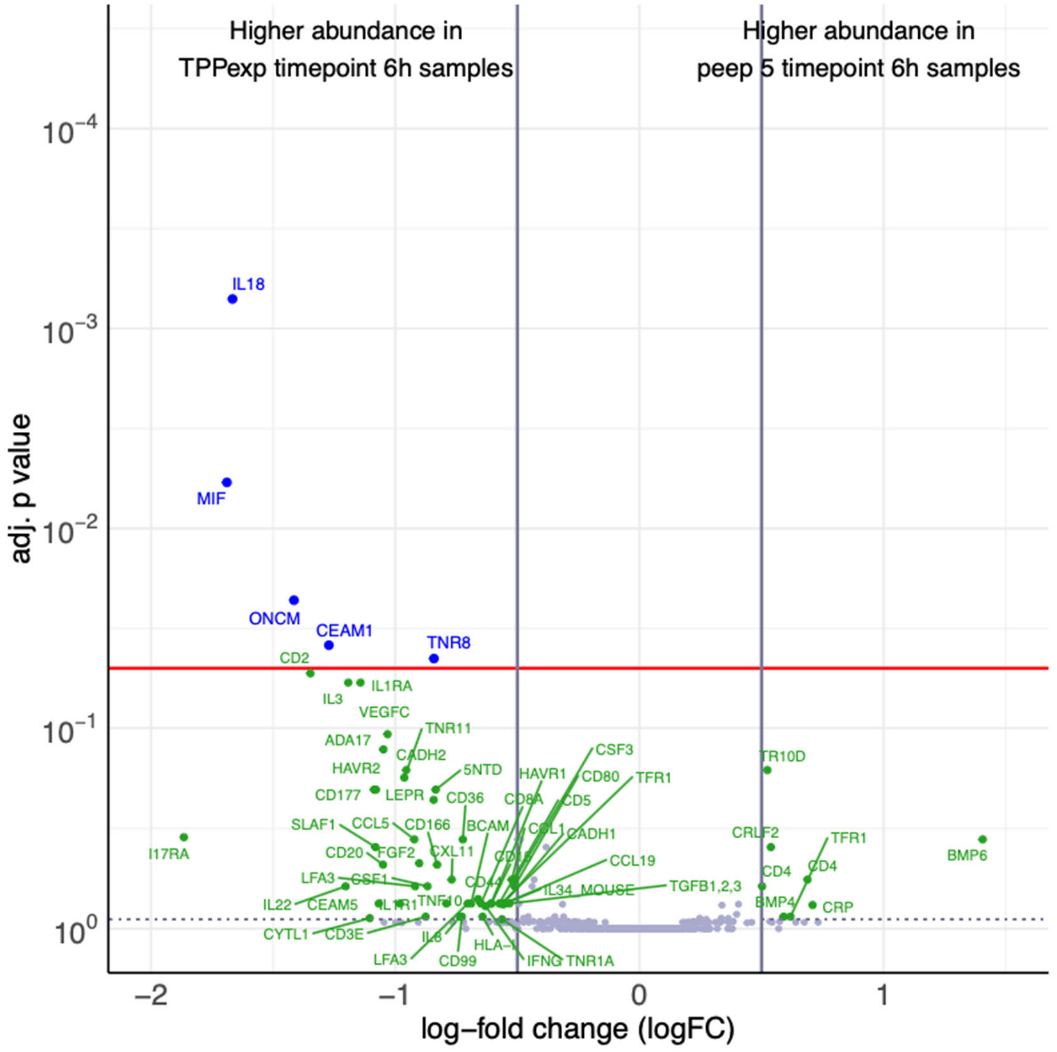

Figure S9: Volcano plot visualizing the differences in abundance between peep 5 timepoint 6h samples and TPPexp timepoint 6h samples as log-fold changes (logFC) and their corresponding p values (adj. for multiple testing). The red line indicates a significance level of adj. p value = 0.05, vertical lines indicate the logFC cutoffs of ±0.5. A positive logFC indicates higher abundance in peep 5 timepoint 6h samples, a negative logFC in TPPexp timepoint 6h samples. Differential proteins (|logFC| > 0.5, adj. p value < 0.05) are displayed with blue names. Non-significant proteins (adj. p value < 0.9) with a |logFC| > 0.5 are defined as noteworthy and displayed with green names.

Protein	AntibodyID	UniprotName	Uniprot-Entry	HGNC	logFC	AveExp	adj.p val
TNR8	ab1423	TNR8_HUMAN	P28908	TNFRSF8	-0.84	9.16	4.5e-02
CEAM1	ab2363	CEAM1_HUMAN	P13688	CEACAM1	-1.27	10.32	3.8e-02
ONCM	ab1885	ONCM_HUMAN	P13725	OSM	-1.41	11.16	2.3e-02
IL18	ab1734	IL18_HUMAN	Q14116	IL18	-1.67	8.49	7.1e-04
MIF	ab1816	MIF_HUMAN	P14174	MIF	-1.69	9.64	5.9e-03

Table S5: Proteins with differential abundance in peep 5 timepoint 6h samples and TPPexp timepoint 6h samples. Proteins with a positive logFC value had a higher abundance in peep 5 timepoint 6h samples, proteins with a negative value in TPPexp timepoint 6h samples. In addition, p values adjusted for multiple testing are listed. The Uniprot-Identifier links to the Uniprot-Entry [21].

Protein	AntibodyID	UniprotName	Uniprot-Entry	HGNC	logFC	AveExp	adj.p val
BMP6	ab2317	BMP6_HUMAN	P22004	BMP6	1.41	11.64	3.6e-01
CRP	ab1802	CRP_HUMAN	P02741	CRP	0.71	9.42	7.6e-01
CD4	ab1626	CD4_HUMAN	P01730	CD4	0.69	9.12	5.7e-01
TFR1	ab2730	TFR1_HUMAN	P02786	TFRC	0.62	10.93	8.7e-01
BMP4	ab1960	BMP4_HUMAN	P12644	BMP4	0.59	9.08	8.7e-01
CRLF2	ab1498	CRLF2_HUMAN	Q9HC73	CRLF2	0.54	8.04	3.9e-01
TR10D	ab1794	TR10D_HUMAN	Q9UBN6	TNFRSF10D	0.52	10.35	1.6e-01
CD4	ab1362	CD4_HUMAN	P01730	CD4	0.50	9.07	6.1e-01
CSF3	ab2014	CSF3_HUMAN	P09919	CSF3	-0.51	11.36	5.7e-01
CD80	ab2460	CD80_HUMAN	P33681	CD80	-0.51	9.38	6.1e-01
CCL1	ab1869	CCL1_HUMAN	P22362	CCL1	-0.52	8.01	5.7e-01
TGFB1,2,3	ab2351		P01137	TGFB1	-0.53	8.22	7.5e-01
IL34_MOUSE	ab1224	IL34_MOUSE	Q8R1R4	Il34	-0.55	9.50	7.5e-01
CCL19	ab1585	CCL19_HUMAN	Q99731	CCL19	-0.55	10.17	7.5e-01
CADH1	ab2412	CADH1_HUMAN	P12830	CDH1	-0.56	8.87	7.6e-01
TNR1A	ab2332	TNR1A_HUMAN	P19438	TNFRSF1A	-0.56	8.61	9.0e-01
TFR1	ab1547	TFR1_HUMAN	P02786	TFRC	-0.57	8.59	7.5e-01
CD5	ab1366	CD5_HUMAN	P06127	CD5	-0.57	9.26	7.5e-01
CD15	ab1394				-0.61	8.50	7.5e-01
HAVR1	ab2781	HAVR1_HUMAN	Q96D42	HAVCR1	-0.61	8.30	7.5e-01
IFNG	ab1836	IFNG_HUMAN	P01579	IFNG	-0.63	8.97	7.7e-01
HLA-I	ab1553				-0.64	13.41	8.7e-01
CD8A	ab1376	CD8A_HUMAN	P01732	CD8A	-0.65	9.47	7.5e-01
CD44	ab1540	CD44_HUMAN	P16070	CD44	-0.66	13.46	7.1e-01
BCAM	ab2290	BCAM_HUMAN	P50895	BCAM	-0.69	12.59	7.5e-01
LFA3	ab1462	LFA3_HUMAN	P19256	CD58	-0.70	10.98	7.5e-01
CD36	ab2783	CD36_HUMAN	P16671	CD36	-0.72	11.68	3.6e-01
CD99	ab1481	CD99_HUMAN	P14209	CD99	-0.73	11.97	8.7e-01
IL8	ab1645	IL8_HUMAN	P10145	CXCL8	-0.74	9.57	8.7e-01
CXL11	ab2115	CXL11_HUMAN	O14625	CXCL11	-0.77	9.46	5.7e-01
TNF10	ab2073	TNF10_HUMAN	P50591	TNFSF10	-0.79	9.85	7.5e-01
CD166	ab2246	CD166_HUMAN	Q13740	ALCAM	-0.83	9.64	4.8e-01
5NTD	ab3694	5NTD_HUMAN	P21589	NT5E	-0.83	14.15	2.0e-01
LEPR	ab2812	LEPR_HUMAN	P48357	LEPR	-0.84	10.38	2.3e-01
CSF1	ab1957	CSF1_HUMAN	P09603	CSF1	-0.87	9.36	6.1e-01
CD3E	ab1358	CD3E_HUMAN	P07766	CD3E	-0.88	9.87	8.7e-01
FGF2	ab2374	FGF2_HUMAN	P09038	FGF2	-0.90	9.48	4.7e-01
LFA3	ab1461	LFA3_HUMAN	P19256	CD58	-0.92	9.85	6.1e-01
CCL5	ab2438	CCL5_HUMAN	P13501	CCL5	-0.92	7.61	3.6e-01
CADH2	ab2760	CADH2_HUMAN	P19022	CDH2	-0.95	10.49	1.6e-01
TNR11	ab1857	TNR11_HUMAN	Q9Y6Q6	TNFRSF11A	-0.96	9.09	1.8e-01
IL1R1	ab2100	IL1R1_HUMAN	P14778	IL1R1	-0.98	10.21	7.5e-01
VEGFC	ab1178	VEGFC_HUMAN	P49767	VEGFC	-1.03	9.24	1.1e-01
ADA17	ab2342	ADA17_HUMAN	P78536	ADAM17	-1.05	11.76	1.3e-01

Continued on next page

Protein	AntibodyID	UniprotName	Uniprot-Entry	HGNC	logFC	AveExp	adj.p val
CD20	ab1594	CD20_HUMAN	P11836	MS4A1	-1.05	10.47	4.8e-01
CEAM5	ab2720	CEAM5_HUMAN	P06731	CEACAM5	-1.07	10.56	7.5e-01
CD177	ab1489	CD177_HUMAN	Q8N6Q3	CD177	-1.08	8.80	2.0e-01
SLAF1	ab2132	SLAF1_HUMAN	Q13291	SLAMF1	-1.08	9.40	3.9e-01
HAVR2	ab2067	HAVR2_HUMAN	Q8TDQ0	HAVCR2	-1.09	10.31	2.0e-01
CYTL1	ab1670	CYTL1_HUMAN	Q9NRR1	CYTL1	-1.10	10.06	8.9e-01
IL1RA	ab2434	IL1RA_HUMAN	P18510	IL1RN	-1.14	12.46	5.9e-02
IL3	ab2172	IL3_HUMAN	P08700	IL3	-1.19	8.30	5.9e-02
IL22	ab1933	IL22_HUMAN	Q9GZX6	IL22	-1.20	8.98	6.1e-01
CD2	ab1356	CD2_HUMAN	P06729	CD2	-1.35	9.48	5.3e-02
I17RA	ab2407	I17RA_HUMAN	Q96F46	IL17RA	-1.87	11.12	3.5e-01

Table S6: Proteins with noteworthy abundance in peep 5 timepoint 6h samples and TPPexp timepoint 6h samples, which feature notable logFCs, while not reaching the significance and logFC thresholds simultaneously. Proteins with a positive logFC value had a higher abundance in peep 5 timepoint 6h samples, proteins with a negative value in TPPexp timepoint 6h samples. In addition, p values adjusted for multiple testing are listed. The Uniprot-Identifier links to the Uniprot-Entry [21].

3.2.5 Timepoint 6h: PEEP 5 vs Sham

Between peep 5 timepoint 6h samples and sham timepoint 6h samples, no antibodies recorded a differential protein abundance. The results of the statistical analysis are summarized in the volcano plot (Figure S10). Furthermore, Table S7 lists noteworthy proteins reaching reduced logFC and significance thresholds.

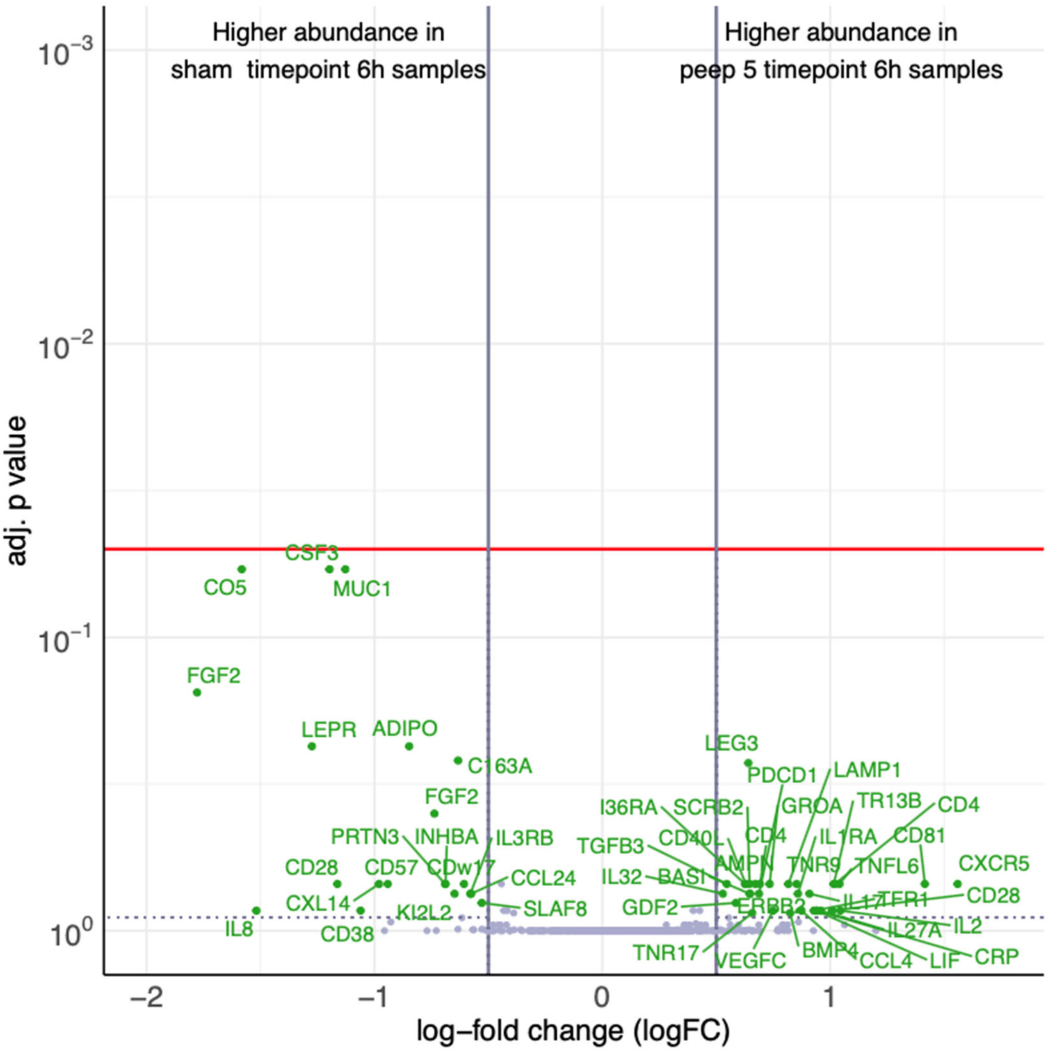

Figure S10: Volcano plot visualizing the differences in abundance between peep 5 timepoint 6h samples and sham timepoint 6h samples as log-fold changes (logFC) and their corresponding p values (adj. for multiple testing). The red line indicates a significance level of adj. p value = 0.05, vertical lines indicate the logFC cutoffs of ±0.5. A positive logFC indicates higher abundance in peep 5 timepoint 6h samples, a negative logFC in sham timepoint 6h samples. Differential proteins (|logFC| > 0.5, adj. p value < 0.05) are displayed with blue names. Non-significant proteins (adj. p value < 0.9) with a |logFC| > 0.5 are defined as noteworthy and displayed with green names.

Protein	AntibodyID	UniprotName	Uniprot-Entry	HGNC	logFC	AveExp	adj.p val
CXCR5	ab3667	CXCR5_HUMAN	P32302	CXCR5	1.56	10.55	6.9e-01
CD81	ab3695	CD81_HUMAN	P60033	CD81	1.42	11.75	6.9e-01
IL2	ab1901	IL2_HUMAN	P60568	IL2	1.04	14.14	8.5e-01
TNFL6	ab1983	TNFL6_HUMAN	P48023	FASLG	1.04	9.43	6.9e-01
CD4	ab1626	CD4_HUMAN	P01730	CD4	1.02	9.12	6.9e-01
TR13B	ab2250	TR13B_HUMAN	O14836	TNFRSF13B	1.02	10.07	6.9e-01
CD28	ab1420	CD28_HUMAN	P10747	CD28	1.01	10.81	8.5e-01
IL27A	ab1741	IL27A_HUMAN	Q8NEV9	IL27	0.96	9.64	8.5e-01
CRP	ab1802	CRP_HUMAN	P02741	CRP	0.94	9.42	8.5e-01
LIF	ab2318	LIF_HUMAN	P15018	LIF	0.94	8.44	8.5e-01
TFR1	ab2730	TFR1_HUMAN	P02786	TFRC	0.93	10.93	8.5e-01
IL17	ab2717	IL17_HUMAN	Q16552	IL17A	0.91	11.78	7.5e-01
CCL4	ab2391	CCL4_HUMAN	P13236	CCL4	0.87	8.68	8.5e-01
IL1RA	ab2434	IL1RA_HUMAN	P18510	IL1RN	0.86	12.46	7.5e-01
TNR9	ab2274	TNR9_HUMAN	Q07011	TNFRSF9	0.85	9.49	6.9e-01
BMP4	ab1960	BMP4_HUMAN	P12644	BMP4	0.82	9.08	8.7e-01
LAMP1	ab3672	LAMP1_HUMAN	P11279	LAMP1	0.82	8.73	6.9e-01
VEGFC	ab1178	VEGFC_HUMAN	P49767	VEGFC	0.75	9.24	8.5e-01
GROA	ab2004	GROA_HUMAN	P09341	CXCL1	0.73	8.46	6.9e-01
CD4	ab1362	CD4_HUMAN	P01730	CD4	0.69	9.07	6.9e-01
PDCD1	ab1352	PDCD1_HUMAN	Q15116	PDCD1	0.69	8.79	7.5e-01
AMPN	ab2383	AMPN_HUMAN	P15144	ANPEP	0.67	8.35	6.9e-01
TNR17	ab2227	TNR17_HUMAN	Q02223	TNFRSF17	0.66	8.38	8.7e-01
ERBB2	ab3697	ERBB2_HUMAN	P04626	ERBB2	0.65	8.66	7.5e-01
BASI	ab1910	BASI_HUMAN	P35613	BSG	0.65	8.51	7.5e-01
SCRB2	ab2146	SCRB2_HUMAN	Q14108	SCARB2	0.65	8.38	6.9e-01
LEG3	ab1738	LEG3_HUMAN	P17931	LGALS3	0.64	8.48	2.7e-01
CD40L	ab2122	CD40L_HUMAN	P29965	CD40LG	0.63	8.34	6.9e-01
I36RA	ab2687	I36RA_HUMAN	Q9UBH0	IL36RN	0.63	9.44	6.9e-01
GDF2	ab2201	GDF2_HUMAN	Q9UK05	GDF2	0.59	8.72	8.0e-01
TGFB3	ab2947	TGFB3_HUMAN	P10600	TGFB3	0.55	6.88	6.9e-01
IL32	ab2749	IL32_HUMAN	P24001	IL32	0.53	9.62	7.5e-01
SLAF8	ab2129	SLAF8_HUMAN	Q9P0V8	SLAMF8	-0.53	12.19	8.0e-01
CCL24	ab1774	CCL24_HUMAN	O00175	CCL24	-0.58	8.62	7.5e-01
IL3RB	ab1551	IL3RB_HUMAN	P32927	CSF2RB	-0.58	8.87	7.5e-01
CDw17	ab1401				-0.61	8.40	6.9e-01
C163A	ab1944	C163A_HUMAN	Q86VB7	CD163	-0.63	11.95	2.6e-01
KI2L2	ab1134	KI2L2_HUMAN	P43627	KIR2DL2	-0.65	9.47	7.5e-01
INHBA	ab2176	INHBA_HUMAN	P08476	INHBA	-0.69	8.52	6.9e-01
PRTN3	ab1501	PRTN3_HUMAN	P24158	PRTN3	-0.69	8.04	6.9e-01
FGF2	ab1590	FGF2_HUMAN	P09038	FGF2	-0.74	8.88	4.0e-01
ADIPO	ab2214	ADIPO_HUMAN	Q15848	ADIPOQ	-0.85	14.61	2.4e-01
CD57	ab1460				-0.94	8.30	6.9e-01
CXL14	ab2300	CXL14_HUMAN	O95715	CXCL14	-0.98	9.39	6.9e-01
CD38	ab1537	CD38_HUMAN	P28907	CD38	-1.06	9.30	8.5e-01
MUC1	ab1087	MUC1_HUMAN	P15941	MUC1	-1.13	11.93	5.9e-02
CD28	ab1559	CD28_HUMAN	P10747	CD28	-1.16	9.84	6.9e-01
CSF3	ab2014	CSF3_HUMAN	P09919	CSF3	-1.20	11.36	5.9e-02
LEPR	ab2812	LEPR_HUMAN	P48357	LEPR	-1.27	10.38	2.4e-01
IL8	ab2312	IL8_HUMAN	P10145	CXCL8	-1.52	9.13	8.5e-01
CO5	ab2011	CO5_HUMAN	P01031	C5	-1.58	8.94	5.9e-02
FGF2	ab2374	FGF2_HUMAN	P09038	FGF2	-1.78	9.48	1.5e-01

Table S7: Proteins with noteworthy abundance in peep 5 timepoint 6h samples and sham timepoint 6h samples, which feature notable logFCs, while not reaching the significance and logFC thresholds simultaneously. Proteins with a positive logFC value had a higher abundance in peep 5 timepoint 6h samples, proteins with a negative value in sham timepoint 6h samples. In addition, p values adjusted for multiple testing are listed. The Uniprot-Identifier links to the Uniprot-Entry [21].

3.2.6 Timepoint 6h: TPPexp vs Sham

Between TPPexp timepoint 6h samples and sham timepoint 6h samples, 8 antibodies recorded a differential protein abundance. The results of the statistical analysis are summarized in the volcano plot (Figure S11) and listed in Table S8. Furthermore, Table S9 lists noteworthy proteins reaching reduced logFC and significance thresholds.

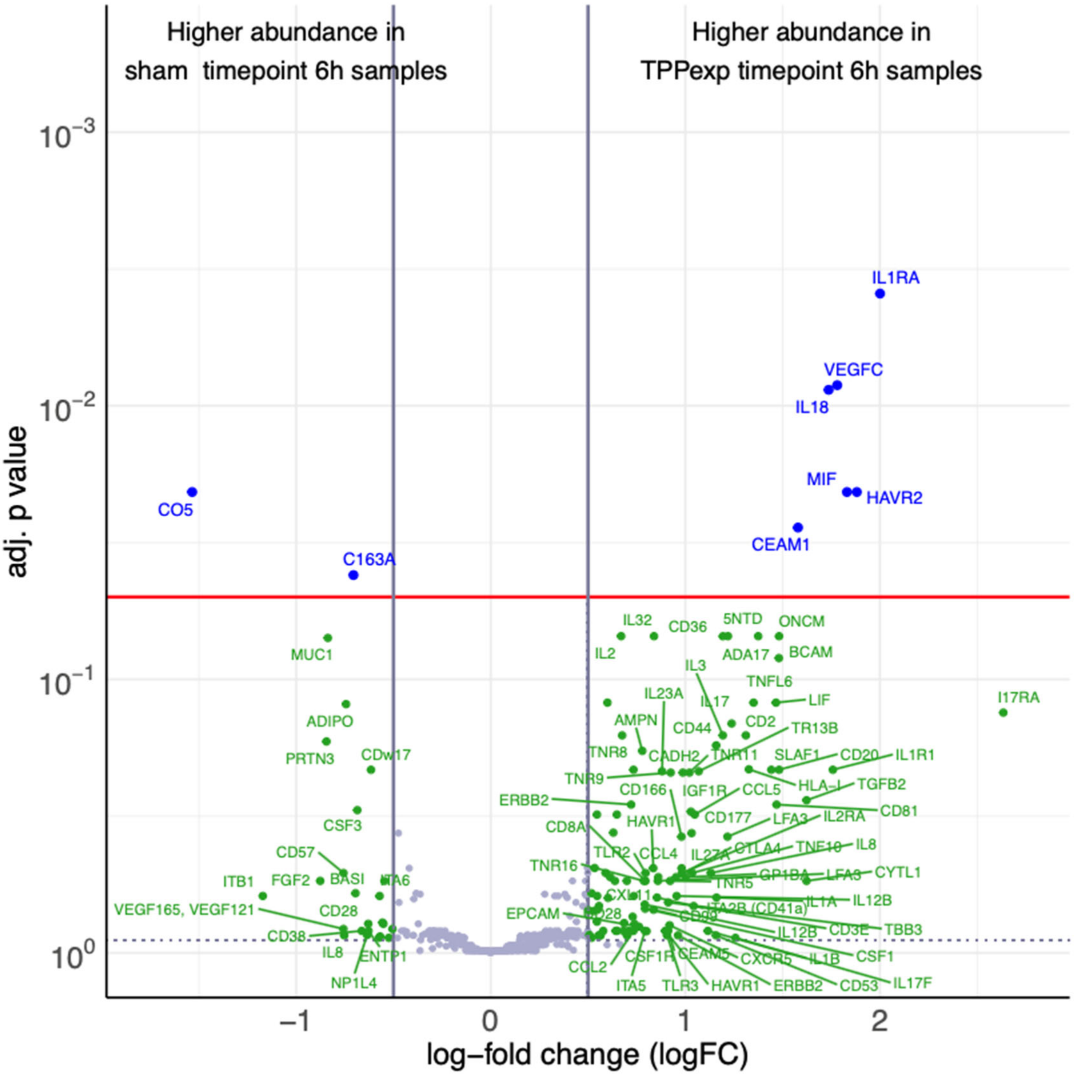

Figure S11: Volcano plot visualizing the differences in abundance between TPPexp timepoint 6h samples and sham timepoint 6h samples as log-fold changes (logFC) and their corresponding p values (adj. for multiple testing). The red line indicates a significance level of adj. p value = 0.05, vertical lines indicate the logFC cutoffs of ±0.5. A positive logFC indicates higher abundance in TPPexp timepoint 6h samples, a negative logFC in sham timepoint 6h samples. Differential proteins (|logFC| > 0.5, adj. p value < 0.05) are displayed with blue names. Non-significant proteins (adj. p value < 0.9) with a |logFC| > 0.5 are defined as noteworthy and displayed with green names.

Protein	AntibodyID	UniprotName	Uniprot-Entry	HGNC	logFC	AveExp	adj.p val
IL1RA	ab2434	IL1RA_HUMAN	P18510	IL1RN	2.00	12.46	3.9e-03
HAVR2	ab2067	HAVR2_HUMAN	Q8TDQ0	HAVCR2	1.88	10.31	2.1e-02
MIF	ab1816	MIF_HUMAN	P14174	MIF	1.83	9.64	2.1e-02
VEGFC	ab1178	VEGFC_HUMAN	P49767	VEGFC	1.78	9.24	8.4e-03
IL18	ab1734	IL18_HUMAN	Q14116	IL18	1.74	8.49	8.7e-03
CEAM1	ab2363	CEAM1_HUMAN	P13688	CEACAM1	1.58	10.32	2.8e-02
C163A	ab1944	C163A_HUMAN	Q86VB7	CD163	-0.71	11.95	4.2e-02
CO5	ab2011	CO5_HUMAN	P01031	C5	-1.53	8.94	2.1e-02

Table S8: Proteins with differential abundance in TPPexp timepoint 6h samples and sham timepoint 6h samples. Proteins with a positive logFC value had a higher abundance in TPPexp timepoint 6h samples, proteins with a negative value in sham timepoint 6h samples. In addition, p values adjusted for multiple testing are listed. The Uniprot-Identifier links to the Uniprot-Entry [21].

Protein	AntibodyID	UniprotName	Uniprot-Entry	HGNC	logFC	AveExp	adj.p val
I17RA	ab2407	I17RA_HUMAN	Q96F46	IL17RA	2.63	11.12	1.3e-01
IL1R1	ab2100	IL1R1_HUMAN	P14778	IL1R1	1.76	10.21	2.1e-01
TGFB2	ab1753	TGFB2_HUMAN	P61812	TGFB2	1.62	10.26	2.8e-01
CYTL1	ab1670	CYTL1_HUMAN	Q9NRR1	CYTL1	1.62	10.06	5.5e-01
ONCM	ab1885	ONCM_HUMAN	P13725	OSM	1.48	11.16	7.0e-02
CD20	ab1594	CD20_HUMAN	P11836	MS4A1	1.48	10.47	2.1e-01
BCAM	ab2290	BCAM_HUMAN	P50895	BCAM	1.48	12.59	8.4e-02
CD81	ab3695	CD81_HUMAN	P60033	CD81	1.47	11.75	2.9e-01
LIF	ab2318	LIF_HUMAN	P15018	LIF	1.47	8.44	1.2e-01
SLAF1	ab2132	SLAF1_HUMAN	Q13291	SLAMF1	1.44	9.40	2.1e-01
ADA17	ab2342	ADA17_HUMAN	P78536	ADAM17	1.37	11.76	7.0e-02
TNFL6	ab1983	TNFL6_HUMAN	P48023	FASLG	1.35	9.43	1.2e-01
HLA-I	ab1553				1.33	13.41	2.1e-01
CD2	ab1356	CD2_HUMAN	P06729	CD2	1.31	9.48	1.6e-01
IL17F	ab2492	IL17F_HUMAN	Q96PD4	IL17F	1.26	8.35	8.8e-01
IL17	ab2717	IL17_HUMAN	Q16552	IL17A	1.24	11.78	1.5e-01
5NTD	ab3694	5NTD_HUMAN	P21589	NT5E	1.22	14.15	7.0e-02
LFA3	ab1461	LFA3_HUMAN	P19256	CD58	1.22	9.85	3.8e-01
CD36	ab2783	CD36_HUMAN	P16671	CD36	1.19	11.68	7.0e-02
IL3	ab2172	IL3_HUMAN	P08700	IL3	1.19	8.30	1.6e-01
IL1A	ab2263	IL1A_HUMAN	P01583	IL1A	1.16	12.82	6.3e-01
CD44	ab1540	CD44_HUMAN	P16070	CD44	1.16	13.46	1.7e-01
CD53	ab1544	CD53_HUMAN	P19397	CD53	1.16	9.91	8.6e-01
IL8	ab1645	IL8_HUMAN	P10145	CXCL8	1.13	9.57	5.1e-01
IL1B	ab1686	IL1B_HUMAN	P01584	IL1B	1.12	10.74	8.3e-01
TR13B	ab2250	TR13B_HUMAN	O14836	TNFRSF13B	1.07	10.07	2.2e-01
CCL5	ab2438	CCL5_HUMAN	P13501	CCL5	1.05	7.61	3.1e-01
CD3E	ab1358	CD3E_HUMAN	P07766	CD3E	1.04	9.87	6.7e-01
TNF10	ab2073	TNF10_HUMAN	P50591	TNFSF10	1.03	9.85	5.1e-01
CD177	ab1489	CD177_HUMAN	Q8N6Q3	CD177	1.03	8.80	3.6e-01
IGF1R	ab1995	IGF1R_HUMAN	P08069	IGF1R	1.03	9.24	3.0e-01
TNR11	ab1857	TNR11_HUMAN	Q9Y6Q6	TNFRSF11A	1.02	9.09	2.2e-01
GP1BA	ab1433	GP1BA_HUMAN	P07359	GP1BA	1.00	10.30	5.3e-01
CADH2	ab2760	CADH2_HUMAN	P19022	CDH2	0.99	10.49	2.2e-01
IL2RA	ab1831	IL2RA_HUMAN	P01589	IL2RA	0.98	11.81	5.1e-01
IL27A	ab1741	IL27A_HUMAN	Q8NEV9	IL27	0.98	9.64	4.9e-01
CD166	ab2246	CD166_HUMAN	Q13740	ALCAM	0.98	9.64	3.8e-01
ERBB2	ab2784	ERBB2_HUMAN	P04626	ERBB2	0.96	9.88	8.6e-01
IL12B	ab1984	IL12B_HUMAN	P29460	IL12B	0.96	11.23	6.2e-01
CTLA4	ab1117	CTLA4_HUMAN	P16410	CTLA4	0.95	12.50	5.3e-01
TNR9	ab2274	TNR9_HUMAN	Q07011	TNFRSF9	0.92	9.49	2.2e-01

Continued on next page

Protein	AntibodyID	UniprotName	Uniprot-Entry	HGNC	logFC	AveExp	adj.p val
TNR5	ab1752	TNR5_HUMAN	P25942	CD40	0.92	9.52	5.5e-01
CXCR5	ab3667	CXCR5_HUMAN	P32302	CXCR5	0.92	10.55	7.9e-01
TBB3	ab1582	TBB3_HUMAN	Q13509	TUBB3	0.91	12.14	6.5e-01
CEAM5	ab2720	CEAM5_HUMAN	P06731	CEACAM5	0.91	10.56	8.3e-01
TLR3	ab2700	TLR3_HUMAN	O15455	TLR3	0.91	13.50	8.6e-01
HAVR1	ab2265	HAVR1_HUMAN	Q96D42	HAVCR1	0.90	8.44	8.3e-01
IL23A	ab2949	IL23A_HUMAN	Q9NPF7	IL23A	0.88	9.17	2.2e-01
CCL4	ab2391	CCL4_HUMAN	P13236	CCL4	0.86	8.68	5.3e-01
LFA3	ab1462	LFA3_HUMAN	P19256	CD58	0.86	10.98	5.5e-01
ITA2B (CD41a)	ab1538		P08514	ITGA2B	0.85	10.03	6.3e-01
IL32	ab2749	IL32_HUMAN	P24001	IL32	0.84	9.62	7.0e-02
CD99	ab1481	CD99_HUMAN	P14209	CD99	0.84	11.97	7.0e-01
HAVR1	ab2781	HAVR1_HUMAN	Q96D42	HAVCR1	0.84	8.30	4.9e-01
CSF1R	ab1925	CSF1R_HUMAN	P07333	CSF1R	0.80	12.73	8.3e-01
TLR2	ab1868	TLR2_HUMAN	O60603	TLR2	0.80	9.09	5.1e-01
CD8A	ab1376	CD8A_HUMAN	P01732	CD8A	0.79	9.47	5.5e-01
CSF1	ab1957	CSF1_HUMAN	P09603	CSF1	0.79	9.36	6.9e-01
ITA5	ab2299	ITA5_HUMAN	P08648	ITGA5	0.79	12.24	8.3e-01
IL12B	ab1731	IL12B_HUMAN	P29460	IL12B	0.79	13.24	6.7e-01
TNR16	ab2445	TNR16_HUMAN	P08138	NGFR	0.79	12.51	5.5e-01
AMPN	ab2383	AMPN_HUMAN	P15144	ANPEP	0.78	8.35	1.8e-01
EPCAM	ab1987	EPCAM_HUMAN	P16422	EPCAM	0.76	9.20	8.0e-01
CD28	ab1420	CD28_HUMAN	P10747	CD28	0.74	10.81	7.8e-01
CXL11	ab2115	CXL11_HUMAN	O14625	CXCL11	0.74	9.46	6.2e-01
TNR8	ab1423	TNR8_HUMAN	P28908	TNFRSF8	0.73	9.16	2.1e-01
CCL2	ab2435	CCL2_HUMAN	P13500	CCL2	0.73	10.33	8.3e-01
AMPN	ab1390	AMPN_HUMAN	P15144	ANPEP	0.73	12.16	7.4e-01
ERBB2	ab3697	ERBB2_HUMAN	P04626	ERBB2	0.72	8.66	2.9e-01
CCR7	ab0987	CCR7_HUMAN	P32248	CCR7	0.71	11.29	8.3e-01
IL22	ab1933	IL22_HUMAN	Q9GZX6	IL22	0.70	8.98	8.6e-01
IL34_MOUSE	ab1224	IL34_MOUSE	Q8R1R4	Il34	0.70	9.50	5.5e-01
GDF15	ab2078	GDF15_HUMAN	Q99988	GDF15	0.69	9.19	7.8e-01
IL2	ab1901	IL2_HUMAN	P60568	IL2	0.68	14.14	8.3e-01
TGFB3	ab2947	TGFB3_HUMAN	P10600	TGFB3	0.68	6.88	1.6e-01
IL2	ab1587	IL2_HUMAN	P60568	IL2	0.67	8.58	7.0e-02
CD3E	ab1359	CD3E_HUMAN	P07766	CD3E	0.65	10.13	8.3e-01
SCRB2	ab2146	SCRB2_HUMAN	Q14108	SCARB2	0.65	8.38	3.1e-01
CCL20	ab2156	CCL20_HUMAN	P78556	CCL20	0.64	9.35	8.3e-01
TSLP	ab2275	TSLP_HUMAN	Q969D9	TSLP	0.64	8.12	5.5e-01
GROA	ab2004	GROA_HUMAN	P09341	CXCL1	0.63	8.46	3.6e-01
PDCD1	ab1352	PDCD1_HUMAN	Q15116	PDCD1	0.61	8.79	5.3e-01
IFNL1	ab2335	IFNL1_HUMAN	Q8IU54	IFNL1	0.60	8.99	6.3e-01
LEG3	ab1738	LEG3_HUMAN	P17931	LGALS3	0.60	8.48	1.2e-01
BMP5	ab2373	BMP5_HUMAN	P22003	BMP5	0.59	9.75	5.1e-01
TREM1	ab2125	TREM1_HUMAN	Q9NP99	TREM1	0.58	9.21	8.3e-01
CD276	ab2437	CD276_HUMAN	Q5ZPR3	CD276	0.56	12.49	8.6e-01
TNF13	ab1086	TNF13_HUMAN	O75888	TNFSF13	0.56	8.87	8.6e-01
TNF14	ab2301	TNF14_HUMAN	O43557	TNFSF14	0.56	10.00	8.6e-01
TNR17	ab2227	TNR17_HUMAN	Q02223	TNFRSF17	0.56	8.38	6.7e-01
TNFA	ab2019	TNFA_HUMAN	P01375	TNF	0.55	8.37	8.7e-01
ICAM3	ab2009	ICAM3_HUMAN	P32942	ICAM3	0.55	7.25	7.0e-01
OX2G	ab1754	OX2G_HUMAN	P41217	CD200	0.55	8.60	6.2e-01
CD5	ab1366	CD5_HUMAN	P06127	CD5	0.55	9.26	7.7e-01
CCL26	ab1599	CCL26_HUMAN	Q9Y258	CCL26	0.55	9.49	3.1e-01
NEP	ab2254	NEP_HUMAN	P08473	MME	0.53	9.38	4.9e-01
TIE2	ab1882	TIE2_HUMAN	Q02763	TEK	0.53	8.26	7.0e-01
IL17C	ab2403	IL17C_HUMAN	Q9P0M4	IL17C	0.52	11.70	8.8e-01
CXL16	ab2515	CXL16_HUMAN	Q9H2A7	CXCL16	0.52	8.42	6.0e-01
HLA-DR	ab1494				0.51	8.62	8.6e-01
CD47	ab2015	CD47_HUMAN	Q08722	CD47	0.50	9.74	7.0e-01
CXL14	ab2300	CXL14_HUMAN	O95715	CXCL14	-0.51	9.39	8.2e-01

Continued on next page

Protein	AntibodyID	UniprotName	Uniprot-Entry	HGNC	logFC	AveExp	adj.p val
DPP4	ab1778	DPP4_HUMAN	P27487	DPP4	-0.52	11.17	8.8e-01
TIMP1	ab1057	TIMP1_HUMAN	P01033	TIMP1	-0.55	8.89	5.5e-01
CXL13	ab2276	CXL13_HUMAN	O43927	CXCL13	-0.55	7.42	7.8e-01
TIMP1	ab1842	TIMP1_HUMAN	P01033	TIMP1	-0.56	8.85	7.7e-01
CD7	ab1371	CD7_HUMAN	P09564	CD7	-0.57	9.66	8.7e-01
ITA6	ab1566	ITA6_HUMAN	P23229	ITGA6	-0.57	9.81	6.2e-01
IGLC1	ab1584	IGLC1_HUMAN	P0CG04	IGLC1	-0.57	11.66	8.8e-01
CDw17	ab1401				-0.62	8.40	2.1e-01
ENTP1	ab1111	ENTP1_HUMAN	P49961	ENTPD1	-0.63	9.52	8.3e-01
CD28	ab1559	CD28_HUMAN	P10747	CD28	-0.63	9.84	7.8e-01
NP1L4	ab3647	NP1L4_HUMAN	Q99733	NAP1L4	-0.64	9.34	8.6e-01
CD38	ab1537	CD38_HUMAN	P28907	CD38	-0.66	9.30	8.3e-01
CSF3	ab2014	CSF3_HUMAN	P09919	CSF3	-0.69	11.36	3.0e-01
BASI	ab1487	BASI_HUMAN	P35613	BSG	-0.70	10.05	6.0e-01
ADIPO	ab2214	ADIPO_HUMAN	Q15848	ADIPOQ	-0.74	14.61	1.2e-01
IL8	ab2312	IL8_HUMAN	P10145	CXCL8	-0.75	9.13	8.6e-01
CD57	ab1460				-0.76	8.30	5.1e-01
VEGF165, VEGF121	ab3680		P15692	VEGFA	-0.76	8.83	8.2e-01
MUC1	ab1087	MUC1_HUMAN	P15941	MUC1	-0.84	11.93	7.1e-02
PRTN3	ab1501	PRTN3_HUMAN	P24158	PRTN3	-0.84	8.04	1.7e-01
FGF2	ab2374	FGF2_HUMAN	P09038	FGF2	-0.88	9.48	5.5e-01
ITB1	ab1534	ITB1_HUMAN	P05556	ITGB1	-1.17	8.48	6.2e-01

Table S9: Proteins with noteworthy abundance in TPPexp timepoint 6h samples and sham timepoint 6h samples, which feature notable logFCs, while not reaching the significance and logFC thresholds simultaneously. Proteins with a positive logFC value had a higher abundance in TPPexp timepoint 6h samples, proteins with a negative value in sham timepoint 6h samples. In addition, p values adjusted for multiple testing are listed. The Uniprot-Identifier links to the Uniprot-Entry [21].

3.2.7 PEEP 5 Timepoint 6h vs Baseline all

Between peep 5 timepoint 6h samples and baseline all samples, no antibodies recorded a differential protein abundance. The results of the statistical analysis are summarized in the volcano plot (Figure S12).

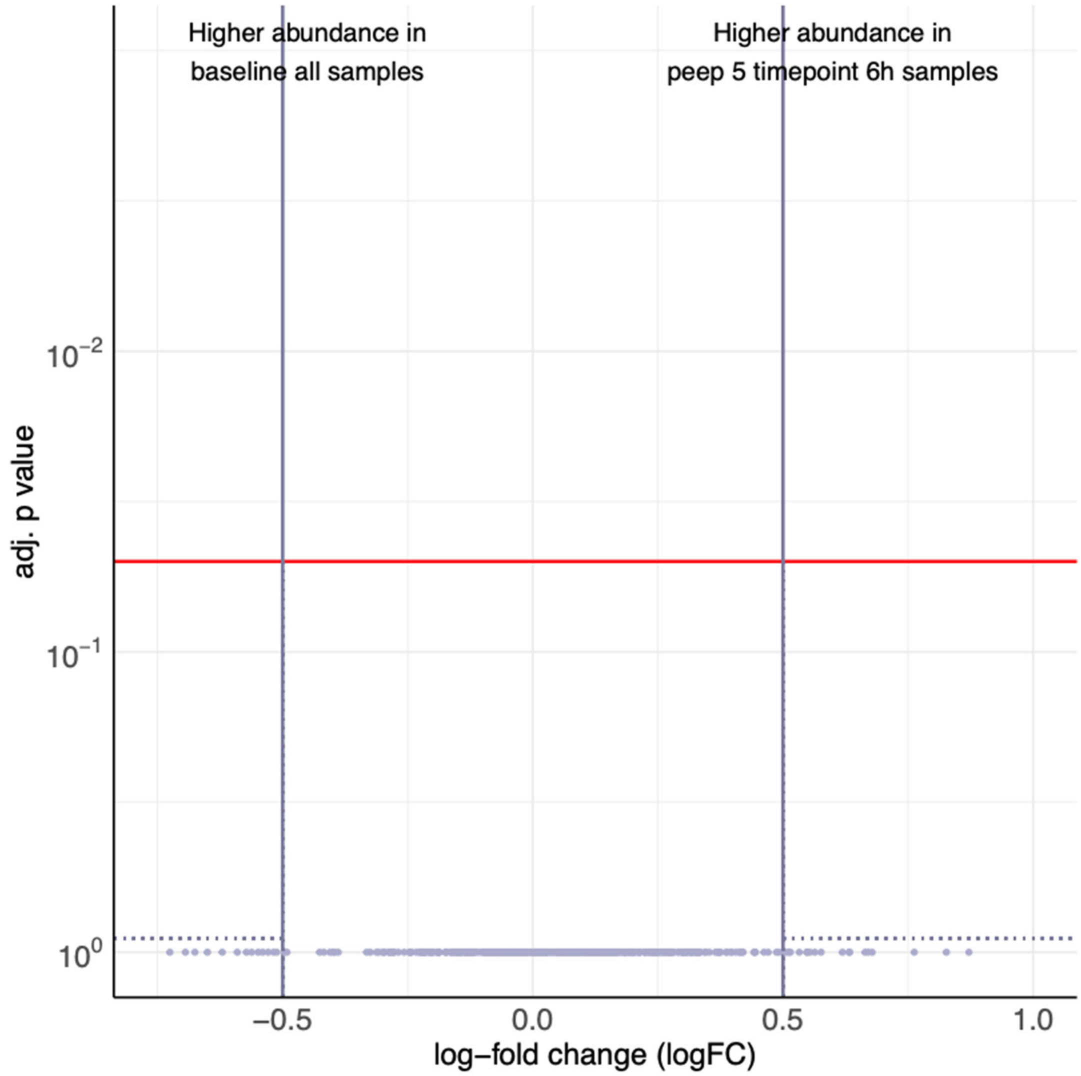

Figure S12: Volcano plot visualizing the differences in abundance between peep 5 timepoint 6h samples and baseline all samples as log-fold changes (logFC) and their corresponding p values (adj. for multiple testing). The red line indicates a significance level of adj. p value = 0.05, vertical lines indicate the logFC cutoffs of ±0.5. A positive logFC indicates higher abundance in peep 5 timepoint 6h samples, a negative logFC in baseline all samples. Differential proteins (|logFC| > 0.5, adj. p value < 0.05) are displayed with blue names. Non-significant proteins (adj. p value < 0.9) with a |logFC| > 0.5 are defined as noteworthy and displayed with green names.

3.2.8 TPPexp Timepoint 6h vs Baseline all

Between TPPexp timepoint 6h samples and baseline all samples, 25 antibodies recorded a differential protein abundance. The results of the statistical analysis are summarized in the volcano plot (Figure S13) and listed in Table S10. Furthermore, Table S11 lists noteworthy proteins reaching reduced logFC and significance thresholds.

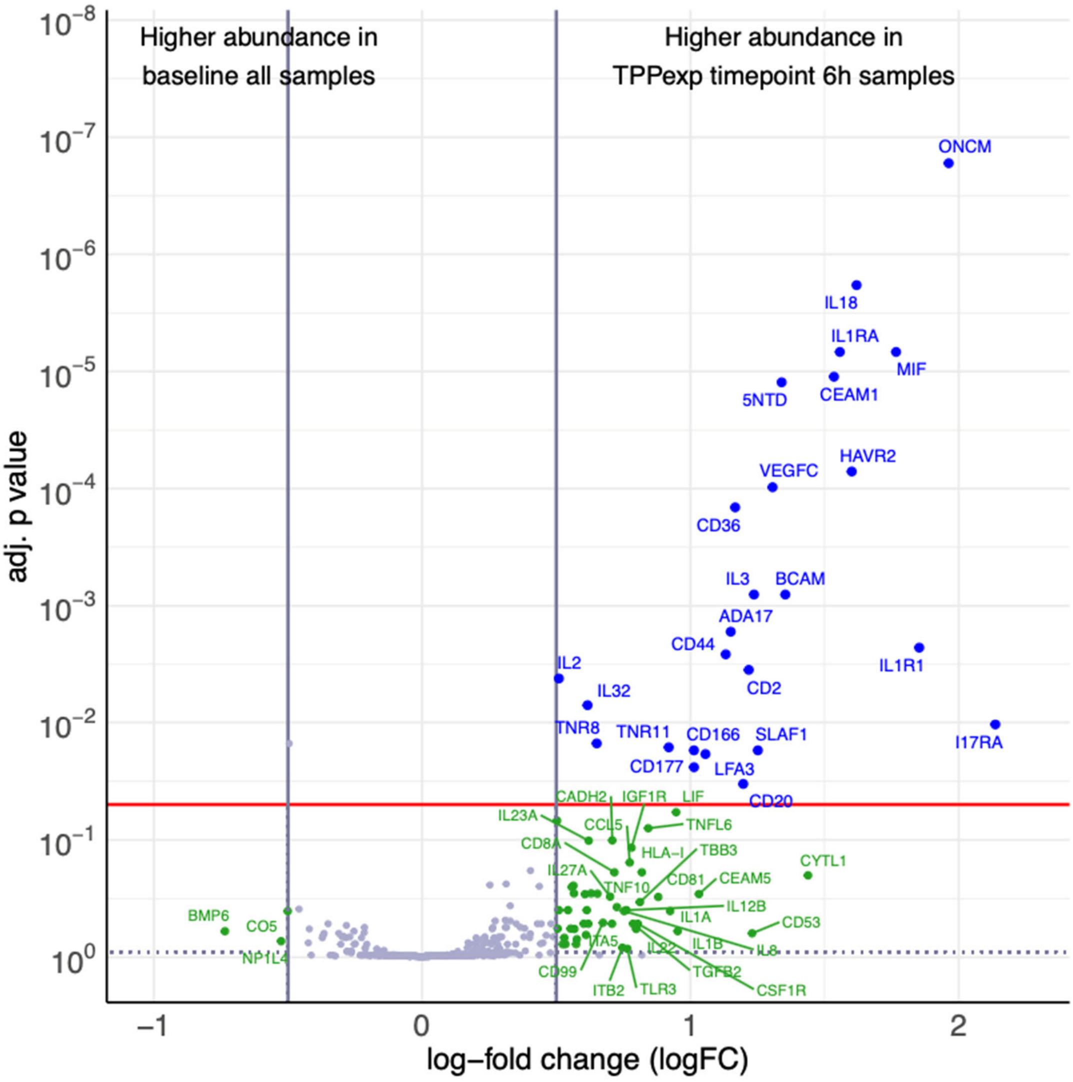

Figure S13: Volcano plot visualizing the differences in abundance between TPPexp timepoint 6h samples and baseline all samples as log-fold changes (logFC) and their corresponding p values (adj. for multiple testing). The red line indicates a significance level of adj. p value = 0.05, vertical lines indicate the logFC cutoffs of ±0.5. A positive logFC indicates higher abundance in TPPexp timepoint 6h samples, a negative logFC in baseline all samples. Differential proteins (|logFC| > 0.5, adj. p value < 0.05) are displayed with blue names. Non-significant proteins (adj. p value < 0.9) with a |logFC| > 0.5 are defined as noteworthy and displayed with green names.

Protein	AntibodyID	UniprotName	Uniprot-Entry	HGNC	logFC	AveExp	adj.p val
I17RA	ab2407	I17RA_HUMAN	Q96F46	IL17RA	2.14	11.12	1.0e-02
ONCM	ab1885	ONCM_HUMAN	P13725	OSM	1.96	11.16	1.7e-07
IL1R1	ab2100	IL1R1_HUMAN	P14778	IL1R1	1.85	10.21	2.3e-03
MIF	ab1816	MIF_HUMAN	P14174	MIF	1.77	9.64	6.8e-06
IL18	ab1734	IL18_HUMAN	Q14116	IL18	1.62	8.49	1.8e-06
HAVR2	ab2067	HAVR2_HUMAN	Q8TDQ0	HAVCR2	1.60	10.31	7.1e-05
IL1RA	ab2434	IL1RA_HUMAN	P18510	IL1RN	1.56	12.46	6.8e-06
CEAM1	ab2363	CEAM1_HUMAN	P13688	CEACAM1	1.54	10.32	1.1e-05
BCAM	ab2290	BCAM_HUMAN	P50895	BCAM	1.35	12.59	8.0e-04
5NTD	ab3694	5NTD_HUMAN	P21589	NT5E	1.34	14.15	1.2e-05
VEGFC	ab1178	VEGFC_HUMAN	P49767	VEGFC	1.31	9.24	9.7e-05
SLAF1	ab2132	SLAF1_HUMAN	Q13291	SLAMF1	1.25	9.40	1.7e-02
IL3	ab2172	IL3_HUMAN	P08700	IL3	1.24	8.30	8.0e-04
CD2	ab1356	CD2_HUMAN	P06729	CD2	1.22	9.48	3.5e-03
CD20	ab1594	CD20_HUMAN	P11836	MS4A1	1.20	10.47	3.3e-02
CD36	ab2783	CD36_HUMAN	P16671	CD36	1.17	11.68	1.4e-04
ADA17	ab2342	ADA17_HUMAN	P78536	ADAM17	1.15	11.76	1.7e-03
CD44	ab1540	CD44_HUMAN	P16070	CD44	1.13	13.46	2.6e-03
LFA3	ab1462	LFA3_HUMAN	P19256	CD58	1.06	10.98	1.9e-02
CD177	ab1489	CD177_HUMAN	Q8N6Q3	CD177	1.01	8.80	2.4e-02
CD166	ab2246	CD166_HUMAN	Q13740	ALCAM	1.01	9.64	1.7e-02
TNR11	ab1857	TNR11_HUMAN	Q9Y6Q6	TNFRSF11A	0.92	9.09	1.6e-02
TNR8	ab1423	TNR8_HUMAN	P28908	TNFRSF8	0.65	9.16	1.5e-02
IL32	ab2749	IL32_HUMAN	P24001	IL32	0.62	9.62	7.1e-03
IL2	ab1587	IL2_HUMAN	P60568	IL2	0.51	8.58	4.2e-03

Table S10: Proteins with differential abundance in TPPexp timepoint 6h samples and baseline all samples. Proteins with a positive logFC value had a higher abundance in TPPexp timepoint 6h samples, proteins with a negative value in baseline all samples. In addition, p values adjusted for multiple testing are listed. The Uniprot-Identifier links to the Uniprot-Entry [21].

Protein	AntibodyID	UniprotName	Uniprot-Entry	HGNC	logFC	AveExp	adj.p val
CYTL1	ab1670	CYTL1_HUMAN	Q9NRR1	CYTL1	1.44	10.06	2.0e-01
CD53	ab1544	CD53_HUMAN	P19397	CD53	1.23	9.91	6.2e-01
CEAM5	ab2720	CEAM5_HUMAN	P06731	CEACAM5	1.03	10.56	2.9e-01
IL1B	ab1686	IL1B_HUMAN	P01584	IL1B	0.95	10.74	6.0e-01
LIF	ab2318	LIF_HUMAN	P15018	LIF	0.95	8.44	5.8e-02
IL1A	ab2263	IL1A_HUMAN	P01583	IL1A	0.92	12.82	4.0e-01
CD81	ab3695	CD81_HUMAN	P60033	CD81	0.88	11.75	3.1e-01
TNFL6	ab1983	TNFL6_HUMAN	P48023	FASLG	0.84	9.43	8.0e-02
HLA-I	ab1553				0.82	13.41	1.9e-01
TBB3	ab1582	TBB3_HUMAN	Q13509	TUBB3	0.81	12.14	3.4e-01
CSF1R	ab1925	CSF1R_HUMAN	P07333	CSF1R	0.80	12.73	5.2e-01
IL22	ab1933	IL22_HUMAN	Q9GZX6	IL22	0.80	8.98	5.7e-01
TGFB2	ab1753	TGFB2_HUMAN	P61812	TGFB2	0.79	10.26	5.2e-01
IGF1R	ab1995	IGF1R_HUMAN	P08069	IGF1R	0.78	9.24	1.2e-01
CCL5	ab2438	CCL5_HUMAN	P13501	CCL5	0.77	7.61	1.6e-01
TLR3	ab2700	TLR3_HUMAN	O15455	TLR3	0.76	13.50	8.5e-01
IL12B	ab1984	IL12B_HUMAN	P29460	IL12B	0.76	11.23	4.0e-01
IL8	ab1645	IL8_HUMAN	P10145	CXCL8	0.75	9.57	4.0e-01
ITB2	ab1402	ITB2_HUMAN	P05107	ITGB2	0.75	11.56	8.3e-01
TNF10	ab2073	TNF10_HUMAN	P50591	TNFSF10	0.73	9.85	3.7e-01
CD8A	ab1376	CD8A_HUMAN	P01732	CD8A	0.72	9.47	1.9e-01
CADH2	ab2760	CADH2_HUMAN	P19022	CDH2	0.71	10.49	1.0e-01
ITA5	ab2299	ITA5_HUMAN	P08648	ITGA5	0.71	12.24	5.2e-01
IL27A	ab1741	IL27A_HUMAN	Q8NEV9	IL27	0.70	9.64	3.0e-01

Continued on next page

Protein	AntibodyID	UniprotName	Uniprot-Entry	HGNC	logFC	AveExp	adj.p val
CD99	ab1481	CD99_HUMAN	P14209	CD99	0.67	11.97	5.1e-01
CXL11	ab2115	CXL11_HUMAN	O14625	CXCL11	0.65	9.46	2.9e-01
IL17	ab2717	IL17_HUMAN	Q16552	IL17A	0.63	11.78	2.9e-01
IL23A	ab2949	IL23A_HUMAN	Q9NPF7	IL23A	0.62	9.17	1.0e-01
GP1BA	ab1433	GP1BA_HUMAN	P07359	GP1BA	0.62	10.30	5.2e-01
TREM1	ab2125	TREM1_HUMAN	Q9NP99	TREM1	0.61	9.21	4.0e-01
CCR7	ab0987	CCR7_HUMAN	P32248	CCR7	0.61	11.29	6.4e-01
TR13B	ab2250	TR13B_HUMAN	O14836	TNFRSF13B	0.61	10.07	2.9e-01
IL2RA	ab1831	IL2RA_HUMAN	P01589	IL2RA	0.60	11.81	5.2e-01
TNF14	ab2301	TNF14_HUMAN	O43557	TNFSF14	0.58	10.00	7.0e-01
IL17C	ab2403	IL17C_HUMAN	Q9P0M4	IL17C	0.57	11.70	7.7e-01
ITA2B (CD41a)	ab1538		P08514	ITGA2B	0.57	10.03	5.7e-01
IL34_MOUSE	ab1224	IL34_MOUSE	Q8R1R4	Il34	0.57	9.50	2.9e-01
TNR9	ab2274	TNR9_HUMAN	Q07011	TNFRSF9	0.56	9.49	2.5e-01
ICAM3	ab2009	ICAM3_HUMAN	P32942	ICAM3	0.56	7.25	2.5e-01
TNR5	ab1752	TNR5_HUMAN	P25942	CD40	0.55	9.52	5.7e-01
HAVR1	ab2781	HAVR1_HUMAN	Q96D42	HAVCR1	0.54	8.30	4.0e-01
TNFA	ab2019	TNFA_HUMAN	P01375	TNF	0.53	8.37	7.7e-01
LFA3	ab1461	LFA3_HUMAN	P19256	CD58	0.53	9.85	6.8e-01
ITA2B	ab1432	ITA2B_HUMAN	P08514	ITGA2B	0.52	9.97	7.7e-01
CD5	ab1366	CD5_HUMAN	P06127	CD5	0.51	9.26	4.0e-01
CD63	ab3703	CD63_HUMAN	P08962	CD63	0.51	12.95	5.7e-01
NEP	ab2254	NEP_HUMAN	P08473	MME	0.50	9.38	6.9e-02
CO5	ab2011	CO5_HUMAN	P01031	C5	-0.50	8.94	4.0e-01
NP1L4	ab3647	NP1L4_HUMAN	Q99733	NAP1L4	-0.53	9.34	7.3e-01
BMP6	ab2317	BMP6_HUMAN	P22004	BMP6	-0.74	11.64	6.0e-01

Table S11: Proteins with noteworthy abundance in TPPexp timepoint 6h samples and baseline all samples, which feature notable logFCs, while not reaching the significance and logFC thresholds simultaneously. Proteins with a positive logFC value had a higher abundance in TPPexp timepoint 6h samples, proteins with a negative value in baseline all samples. In addition, p values adjusted for multiple testing are listed. The Uniprot-Identifier links to the Uniprot-Entry [21].

3.2.9 Sham Timepoint 6h vs Baseline all

Between sham timepoint 6h samples and baseline all samples, no antibodies recorded a differential protein abundance. The results of the statistical analysis are summarized in the volcano plot (Figure S14).

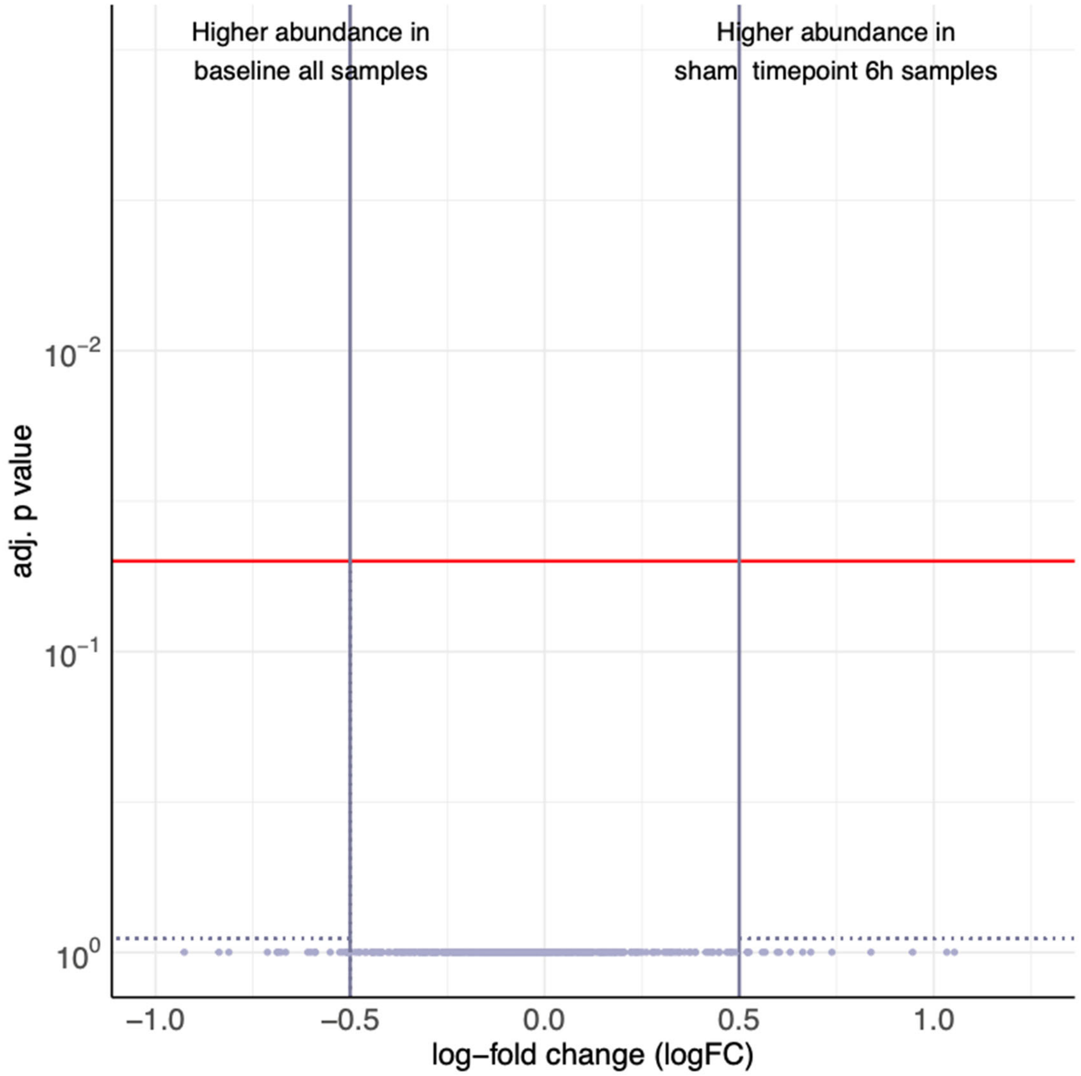

Figure S14: Volcano plot visualizing the differences in abundance between sham timepoint 6h samples and baseline all samples as log-fold changes (logFC) and their corresponding p values (adj. for multiple testing). The red line indicates a significance level of adj. p value = 0.05, vertical lines indicate the logFC cutoffs of ±0.5. A positive logFC indicates higher abundance in sham timepoint 6h samples, a negative logFC in baseline all samples. Differential proteins (|logFC| > 0.5, adj. p value < 0.05) are displayed with blue names. Non-significant proteins (adj. p value < 0.9) with a |logFC| > 0.5 are defined as noteworthy and displayed with green names.

3.3 Individual protein levels

For some differential proteins, relative protein levels are presented in figures S15 and S16.

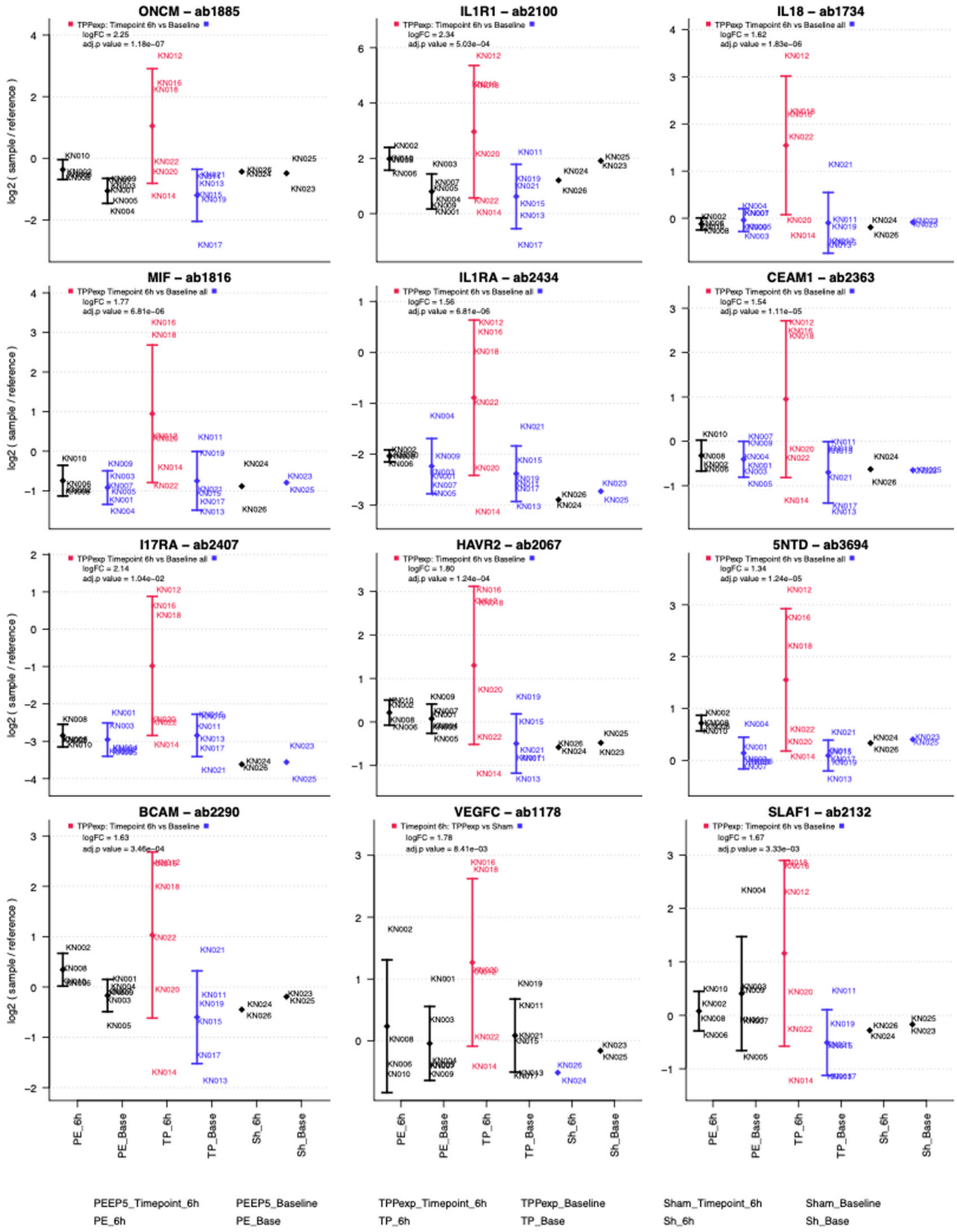

Figure S15: Individual array values for a set of differential proteins. Each sample is measured by four replicate spots per array. Diamonds indicate sample group means. Whiskers indicate one standard deviation.

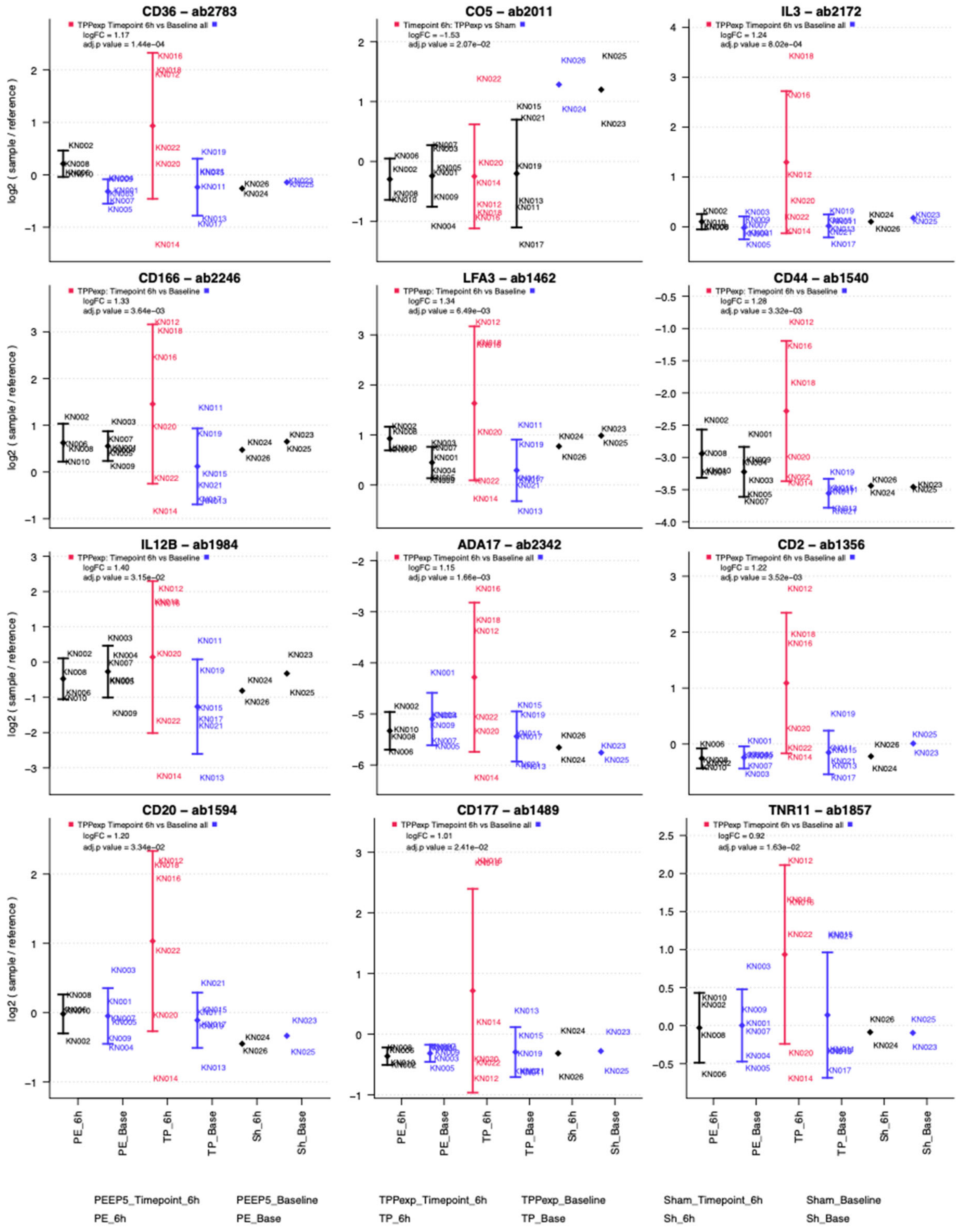

Figure S16: Individual array values for a set of differential proteins. Each sample is measured by four replicate spots per array. Diamonds indicate sample group means. Whiskers indicate one standard deviation.

3.4 Heatmap of differential proteins

Relative expression levels for all identified differential proteins are summarized in Figure S17.

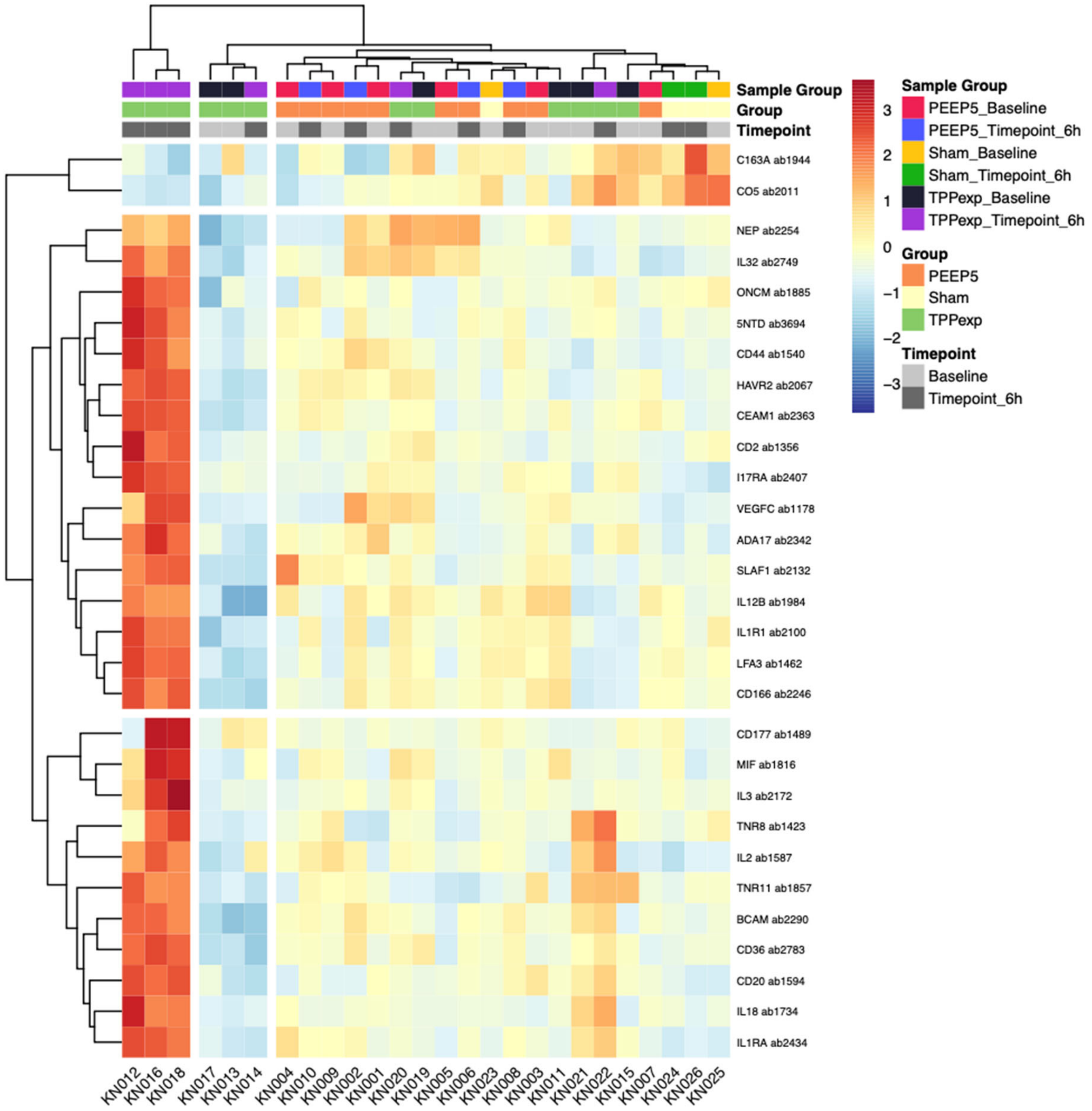

Figure S17: Heatmap displaying the relative expression of proteins identified as differential. Values were centered and scaled by proteins.

3.5 Functional annotation of differential proteins

3.5.1 Differential and noteworthy proteins in TPPexp Timepoint 6h vs Baseline all

The following tables display annotation information about:
- selected KEGG pathways [74] (Table S12)
- selected reactome pathways [75] (Table S13)
- selected WikiPathways [76] (Table S14)

related to differentially abundant proteins identified in this study within the comparison TPPexp Timepoint 6h vs Baseline all, including noteworthy proteins passing reduced logFC and p value thresholds according to section 3.2.8.

Pathway ID<br>Pathway Description	Protein Count	Proteins • no regulation ○ noteworthy proteins passing reduced logFC and p value thresholds as described in section TPPexp Timepoint 6h vs Baseline all<br>• higher protein abundance in TPPexp timepoint 6h samples • higher protein abundance in baseline all samples
hsa04060<br>Cytokine-cytokine receptor interaction	**33** proteins	BMP6 ○ CCL5 ○ CCR7 ○ TNR5 ○ CSF1R ○ CXL11 ○ IL8 ○ TNFL6 ○ IL12B ○ IL17 ○ IL17C ○ I17RA • IL18 • IL1A ○ IL1B ○ IL1R1 • IL1RA • IL2 • IL22 ○ IL23A ○ IL27A ○ IL2RA ○ IL3 • IL32 • LIF ○ ONCM • TNFA ○ TNR11 • TR13B ○ TNR8 • TNR9 ○ TNF10 ○ TNF14 ○
hsa04640<br>Hematopoietic cell lineage	**17** proteins	CD2 • CD36 • CD44 • CD5 ○ CD8A ○ CSF1R ○ GP1BA ○ IL1A ○ IL1B ○ IL1R1 • IL2RA ○ IL3 • ITA2B (CD41a) / ITA2B ○ ITA5 ○ NEP ○ CD20 • TNFA ○
hsa04151<br>PI3K-Akt signaling pathway	**10** proteins	CSF1R ○ TNFL6 ○ IGF1R ○ IL2 • IL2RA ○ IL3 • ITA2B (CD41a) / ITA2B ○ ITA5 ○ ONCM • VEGFC •
hsa04514<br>Cell adhesion molecules	**9** proteins	CD166 • CD2 • TNR5 ○ LFA3 • CD8A ○ CD99 ○ CADH2 ○ ICAM3 ○ ITB2 ○
hsa04620<br>Toll-like receptor signaling pathway	**8** proteins	CCL5 ○ TNR5 ○ CXL11 ○ IL8 ○ IL12B ○ IL1B ○ TLR3 ○ TNFA ○
hsa04630<br>JAK-STAT signaling pathway	**8** proteins	IL12B ○ IL2 • IL22 ○ IL23A ○ IL2RA ○ IL3 • LIF ○ ONCM •
hsa04010<br>MAPK signaling pathway	**8** proteins	CSF1R ○ TNFL6 ○ IGF1R ○ IL1A ○ IL1B ○ IL1R1 • TNFA ○ VEGFC •
hsa04659<br>Th17 cell differentiation	**7** proteins	IL17 ○ IL1B ○ IL1R1 • IL2 • IL22 ○ IL23A ○ IL2RA ○
hsa04064<br>NF-kappa B signaling pathway	**7** proteins	TNR5 ○ IL8 ○ IL1B ○ IL1R1 • TNFA ○ TNR11 • TNF14 ○
hsa04657<br>IL-17 signaling pathway	**6** proteins	IL8 ○ IL17 ○ IL17C ○ I17RA • IL1B ○ TNFA ○
hsa04217<br>Necroptosis	**6** proteins	TNFL6 ○ IL1A ○ IL1B ○ TLR3 ○ TNFA ○ TNF10 ○
hsa04512<br>ECM-receptor interaction	**5** proteins	CD36 • CD44 • GP1BA ○ ITA2B (CD41a) / ITA2B ○ ITA5 ○
hsa04625<br>C-type lectin receptor signaling pathway	**5** proteins	IL12B ○ IL1B ○ IL2 • IL23A ○ TNFA ○
hsa04668<br>TNF signaling pathway	**5** proteins	CCL5 ○ IL1B ○ LIF ○ TNFA ○ VEGFC •
hsa05418<br>Fluid shear stress and atherosclerosis	**5** proteins	IL1A ○ IL1B ○ IL1R1 • ITA2B (CD41a) / ITA2B ○ TNFA ○
hsa04621<br>NOD-like receptor signaling pathway	**5** proteins	CCL5 ○ IL8 ○ IL18 • IL1B ○ TNFA ○
hsa04015<br>Rap1 signaling pathway	**5** proteins	CSF1R ○ IGF1R ○ ITA2B (CD41a) / ITA2B ○ ITB2 ○ VEGFC •
hsa04650<br>Natural killer cell mediated cytotoxicity	**4** proteins	TNFL6 ○ ITB2 ○ TNFA ○ TNF10 ○

Continued on next page

Pathway ID<br>Pathway Description	Protein Count	Proteins • no regulation ∘ noteworthy proteins passing reduced logFC and p value thresholds as described in section TPPexp Timepoint 6h vs Baseline all<br>• higher protein abundance in TPPexp timepoint 6h samples • higher protein abundance in baseline all samples
hsa04210<br>Apoptosis	4 proteins	TNFL6 ∘ IL3 • TNFA ∘ TNF10 ∘
hsa04145<br>Phagosome	4 proteins	CD36 • ITA5 ∘ ITB2 ∘ TBB3 ∘
hsa04062<br>Chemokine signaling pathway	4 proteins	CCL5 ∘ CCR7 ∘ CXL11 ∘ IL8 ∘
hsa04510<br>Focal adhesion	4 proteins	IGF1R ∘ ITA2B (CD41a) / ITA2B ∘ ITA5 ∘ VEGFC •
hsa04014<br>Ras signaling pathway	4 proteins	CSF1R ∘ TNFL6 ∘ IGF1R ∘ VEGFC •
hsa05310<br>Asthma	3 proteins	TNR5 ∘ IL3 • TNFA ∘
hsa04623<br>Cytosolic DNA-sensing pathway	3 proteins	CCL5 ∘ IL18 • IL1B ∘
hsa04622<br>RIG-I-like receptor signaling pathway	3 proteins	IL8 ∘ IL12B ∘ TNFA ∘
hsa04658<br>Th1 and Th2 cell differentiation	3 proteins	IL12B ∘ IL2 • IL2RA ∘
hsa04660<br>T cell receptor signaling pathway	3 proteins	CD8A ∘ IL2 • TNFA ∘

Tabel S12: Selected KEGG pathways [74] related to proteins with differential abundance in TPPexp Timepoint 6h vs Baseline all.

Pathway ID<br>Pathway Description	Protein Count	Proteins ● no regulation ○ noteworthy proteins passing reduced logFC and p value thresholds as described in section TPPexp Timepoint 6h vs Baseline all<br>● higher protein abundance in TPPexp timepoint 6h samples ● higher protein abundance in baseline all samples
HSA-1280215<br>Cytokine Signaling in Immune system	**35** proteins	ADA17 ● CCL5 ○ CD36 ● TNR5 ○ CD44 ● CSF1R ○ IL8 ○ TNFL6 ○ HAVR2 ● IL12B ○ IL17 ○ IL17C ○ I17RA ● IL18 ● IL1A ○ IL1B ○ IL1R1 ● IL1RA ● IL2 ● IL22 ○ IL23A ○ IL27A ○ IL2RA ○ IL3 ● IL32 ● ITB2 ○ LIF ○ MIF ● ONCM ● TNFA ○ TNR11 ● TR13B ○ TNR8 ● TNR9 ○ TNF14 ○
HSA-449147<br>Signaling by Interleukins	**27** proteins	CCL5 ○ CD36 ● CSF1R ○ IL8 ○ TNFL6 ○ HAVR2 ● IL12B ○ IL17 ○ IL17C ○ I17RA ● IL18 ● IL1A ○ IL1B ○ IL1R1 ● IL1RA ● IL2 ● IL22 ○ IL23A ○ IL27A ○ IL2RA ○ IL3 ● IL32 ● ITB2 ○ LIF ○ MIF ● ONCM ● TNFA ○
HSA-6785807<br>Interleukin-4 and Interleukin-13 signaling	**13** proteins	CD36 ● IL8 ○ TNFL6 ○ IL12B ○ IL17 ○ IL18 ● IL1A ○ IL1B ○ IL23A ○ ITB2 ○ LIF ○ ONCM ● TNFA ○
HSA-202733<br>Cell surface interactions at the vascular wall	**11** proteins	CD177 ● CD2 ● CD44 ● LFA3 ● CD99 ○ CEAM1 ● CEAM5 ○ ITA5 ○ ITB2 ○ MIF ● TREM1 ○
HSA-6783783<br>Interleukin-10 signaling	**10** proteins	CCL5 ○ IL8 ○ IL12B ○ IL18 ● IL1A ○ IL1B ○ IL1R1 ● IL1RA ● LIF ○ TNFA ○
HSA-6798695<br>Neutrophil degranulation	**10** proteins	CD177 ● CD36 ● CD44 ● CD53 ○ LFA3 ● CD63 ○ CEAM1 ● ITB2 ○ MIF ● NEP ○
HSA-1474244<br>Extracellular matrix organization	**8** proteins	ADA17 ● CD44 ● CEAM1 ● ICAM3 ○ ITA2B (CD41a) / ITA2B ○ ITA5 ○ ITB2 ○ TGFB2 ○
HSA-5668541<br>TNFR2 non-canonical NF-kB pathway	**7** proteins	TNR5 ○ TNFA ○ TNR11 ● TR13B ○ TNR8 ● TNR9 ○ TNF14 ○
HSA-198933<br>Immunoregulatory interactions between a Lymphoid and a non-Lymphoid cell	**7** proteins	TNR5 ○ CD81 ○ CD8A ○ CD99 ○ ICAM3 ○ ITB2 ○ TREM1 ○
HSA-76002<br>Platelet activation, signaling and aggregation	**6** proteins	CD36 ● CD63 ○ GP1BA ○ ITA2B (CD41a) / ITA2B ○ TGFB2 ○ VEGFC ●
HSA-5218859<br>Regulated Necrosis	**5** proteins	TNFL6 ○ IL18 ● IL1A ○ IL1B ○ TNF10 ○
HSA-216083<br>Integrin cell surface interactions	**5** proteins	CD44 ● ICAM3 ○ ITA2B (CD41a) / ITA2B ○ ITA5 ○ ITB2 ○
HSA-114608<br>Platelet degranulation	**5** proteins	CD36 ● CD63 ○ ITA2B (CD41a) / ITA2B ○ TGFB2 ○ VEGFC ●
HSA-446652<br>Interleukin-1 family signaling	**5** proteins	IL18 ● IL1A ○ IL1B ○ IL1R1 ● IL1RA ●
HSA-5669034<br>TNFs bind their physiological receptors	**4** proteins	TR13B ○ TNR8 ● TNR9 ○ TNF14 ○
HSA-451927<br>Interleukin-2 family signaling	**4** proteins	HAVR2 ● IL2 ● IL2RA ○ IL3 ●
HSA-447115<br>Interleukin-12 family signaling	**4** proteins	IL12B ○ IL23A ○ IL27A ○ MIF ●
HSA-380108<br>Chemokine receptors bind chemokines	**4** proteins	CCL5 ○ CCR7 ○ CXL11 ○ IL8 ○

Continued on next page

Pathway ID<br>Pathway Description	Protein Count	Proteins • no regulation ○ noteworthy proteins passing reduced logFC and p value thresholds as described in section TPPexp Timepoint 6h vs Baseline all<br>• higher protein abundance in TPPexp timepoint 6h samples • higher protein abundance in baseline all samples
HSA-448706<br>Interleukin-1 processing	**3** proteins	IL18 • IL1A ○ IL1B ○
HSA-5676594<br>TNF receptor superfamily (TNFSF) members mediating non-canonical NF-kB pathway	**3** proteins	TNR5 ○ TNR11 • TNF14 ○
HSA-5620971<br>Pyroptosis	**3** proteins	IL18 • IL1A ○ IL1B ○
HSA-912526<br>Interleukin receptor SHC signaling	**3** proteins	IL2 • IL2RA ○ IL3 •
HSA-1566977<br>Fibronectin matrix formation	**2** proteins	CEAM1 • ITA5 ○

Tabel S13: Selected reactome pathways [75] related to proteins with differential abundance in TPPexp Timepoint 6h vs Baseline all.

Pathway ID<br>Pathway Description	Protein Count	Proteins • no regulation ◦ noteworthy proteins passing reduced logFC and p value thresholds as described in section TPPexp Timepoint 6h vs Baseline all<br>• higher protein abundance in TPPexp timepoint 6h samples • higher protein abundance in baseline all samples
WP5095<br>Overview of proinflammatory and profibrotic mediators	**18** proteins	CCL5 ◦ CXL11 ◦ IL8 ◦ IL12B ◦ IL17 ◦ IL17C ◦ IL18 • IL1A ◦ IL1B ◦ IL1RA • IL2 • IL22 ◦ IL23A ◦ IL27A ◦ IL3 • LIF ◦ ONCM • TNFA ◦
WP4754<br>IL-18 signaling pathway	**11** proteins	CCL5 ◦ CD36 • CD81 ◦ IL8 ◦ TNFL6 ◦ IL12B ◦ IL18 • IL1B ◦ IL2RA ◦ ITA2B (CD41a) / ITA2B ◦ TNFA ◦
WP4172<br>PI3K-Akt signaling pathway	**10** proteins	CSF1R ◦ TNFL6 ◦ IGF1R ◦ IL2 • IL2RA ◦ IL3 • ITA2B (CD41a) / ITA2B ◦ ITA5 ◦ ONCM • VEGFC •
WP3932<br>Focal adhesion: PI3K-Akt-mTOR-signaling pathway	**9** proteins	CSF1R ◦ IGF1R ◦ IL2 • IL2RA ◦ ITA2B (CD41a) / ITA2B ◦ ITA5 ◦ ITB2 ◦ ONCM • VEGFC •
WP75<br>Toll-like receptor signaling pathway	**8** proteins	CCL5 ◦ TNR5 ◦ CXL11 ◦ IL8 ◦ IL12B ◦ IL1B ◦ TLR3 ◦ TNFA ◦
WP3893<br>Development and heterogeneity of the ILC family	**7** proteins	IL12B ◦ IL17 ◦ IL18 • IL1B ◦ IL22 ◦ IL23A ◦ TNFA ◦
WP5130<br>Th17 cell differentiation pathway	**7** proteins	IL17 ◦ IL1B ◦ IL2 • IL22 ◦ IL23A ◦ IL27A ◦ IL2RA ◦
WP4541<br>Hippo-Merlin signaling dysregulation	**7** proteins	CD44 • CADH2 ◦ CSF1R ◦ IGF1R ◦ ITA2B (CD41a) / ITA2B ◦ ITA5 ◦ ITB2 ◦
WP530<br>Cytokines and inflammatory response	**6** proteins	IL12B ◦ IL1A ◦ IL1B ◦ IL2 • IL3 • TNFA ◦
WP4496<br>Signal transduction through IL1R	**6** proteins	IL1A ◦ IL1B ◦ IL1R1 • IL1RA • TGFB2 ◦ TNFA ◦
WP382<br>MAPK signaling pathway	**6** proteins	TNFL6 ◦ IL1A ◦ IL1B ◦ IL1R1 • TGFB2 ◦ TNFA ◦
WP4493<br>Cells and molecules involved in local acute inflammatory response	**5** proteins	CO5 ◦ IL8 ◦ IL1A ◦ ITB2 ◦ TNFA ◦
WP4478<br>LTF danger signal response pathway	**5** proteins	IL8 ◦ IL1A ◦ IL1B ◦ TNFA ◦ TREM1 ◦
WP5088<br>Prostaglandin signaling	**5** proteins	IL8 ◦ IL17 ◦ IL1A ◦ IL1B ◦ TNFA ◦
WP3617<br>Photodynamic therapy-induced NF-kB survival signaling	**5** proteins	IL8 ◦ IL1A ◦ IL1B ◦ IL2 • TNFA ◦
WP3624<br>Lung fibrosis	**5** proteins	CCL5 ◦ IL8 ◦ IL12B ◦ IL1B ◦ TNFA ◦
WP69<br>T-cell receptor signaling pathway	**5** proteins	CD8A ◦ IL17 ◦ IL1A ◦ IL1B ◦ TNR9 ◦

Continued on next page

Pathway ID<br>Pathway Description	Protein Count	Proteins • no regulation ◦ noteworthy proteins passing reduced logFC and p value thresholds as described in section TPPexp Timepoint 6h vs Baseline all<br>• higher protein abundance in TPPexp timepoint 6h samples • higher protein abundance in baseline all samples
WP5322<br>CKAP4 signaling pathway map	**5** proteins	CD44 • ITA5 ◦ MIF • TGFB2 ◦ TNFA ◦
WP2877<br>Vitamin D receptor pathway	**5** proteins	BMP6 ◦ TNR5 ◦ CEAM1 • TGFB2 ◦ TREM1 ◦
WP2882<br>Nuclear receptors meta-pathway	**5** proteins	IL12B ◦ IL1B ◦ IL2 • TGFB2 ◦ TNFA ◦
WP4462<br>Platelet-mediated interactions with vascular and circulating cells	**4** proteins	CCL5 ◦ TNR5 ◦ IL1B ◦ TGFB2 ◦
WP4494<br>Selective expression of chemokine receptors during T-cell polarization	**4** proteins	CCR7 ◦ IL12B ◦ IL2 • TGFB2 ◦
WP4136<br>Fibrin complement receptor 3 signaling pathway	**4** proteins	IL12B ◦ ITB2 ◦ TLR3 ◦ TNFA ◦
WP2873<br>Aryl hydrocarbon receptor pathway	**4** proteins	IL12B ◦ IL1B ◦ IL2 • TNFA ◦
WP2849<br>Hematopoietic stem cell differentiation	**4** proteins	IL1A ◦ IL1B ◦ IL3 • ITA2B (CD41a) / ITA2B ◦
WP254<br>Apoptosis	**4** proteins	TNFL6 ◦ IGF1R ◦ TNFA ◦ TNF10 ◦
WP2806<br>Complement system	**4** proteins	CO5 ◦ TNR5 ◦ ITA2B (CD41a) / ITA2B ◦ ITB2 ◦
WP5090<br>Complement system in neuronal development and plasticity	**4** proteins	CO5 ◦ TNFL6 ◦ ITB2 ◦ TGFB2 ◦
WP236<br>Adipogenesis	**4** proteins	LIF ◦ MIF • ONCM • TNFA ◦
WP306<br>Focal adhesion	**4** proteins	IGF1R ◦ ITA2B (CD41a) / ITA2B ◦ ITA5 ◦ VEGFC •
WP453<br>Inflammatory response pathway	**3** proteins	TNR5 ◦ IL2 • IL2RA ◦
WP2112<br>IL-17 signaling pathway	**3** proteins	IL17 ◦ IL17C ◦ I17RA •
WP4481<br>Resistin as a regulator of inflammation	**3** proteins	IL8 ◦ IL12B ◦ TNFA ◦
WP727<br>Monoamine transport	**3** proteins	IL1B ◦ IL1R1 • TNFA ◦
WP5097<br>CCL18 signaling pathway	**3** proteins	CD44 • CADH2 ◦ VEGFC •

Continued on next page

Pathway ID<br>Pathway Description	Protein Count	Proteins ● no regulation ○ noteworthy proteins passing reduced logFC and p value thresholds as described in section TPPexp Timepoint 6h vs Baseline all<br>● higher protein abundance in TPPexp timepoint 6h samples ● higher protein abundance in baseline all samples
WP314<br>Fas ligand pathway and stress induction of heat shock proteins	**3** proteins	TNFL6 ○ IL1A ○ TNFA ○
WP2848<br>Pluripotent stem cell differentiation pathway	**3** proteins	CSF1R ○ IL3 ● 5NTD ●
WP5300<br>TROP2 regulatory signaling	**3** proteins	ADA17 ● IGF1R ○ ITA5 ○
WP1533<br>Vitamin B12 metabolism	**3** proteins	CCL5 ○ IL1B ○ TNFA ○
WP195<br>IL-1 signaling pathway	**3** proteins	IL1A ○ IL1B ○ IL1R1 ●
WP176<br>Folate metabolism	**3** proteins	IL1B ○ IL2 ● TNFA ○
WP4655<br>Cytosolic DNA-sensing pathway	**3** proteins	CCL5 ○ IL18 ● IL1B ○
WP4341<br>Non-genomic actions of 1,25 dihydroxyvitamin D3	**3** proteins	TNR5 ○ IL8 ○ TNFA ○
WP1772<br>Apoptosis modulation and signaling	**3** proteins	TNFL6 ○ IL1R1 ● TNF10 ○
WP4963<br>p53 transcriptional gene network	**3** proteins	TNFL6 ○ LIF ○ TNFA ○
WP2355<br>Corticotropin-releasing hormone signaling pathway	**3** proteins	IL8 ○ IL18 ● IL2 ●
WP4540<br>Hippo signaling regulation pathways	**3** proteins	CADH2 ○ CSF1R ○ IGF1R ○
WP185<br>Integrin-mediated cell adhesion	**3** proteins	ITA2B (CD41a) / ITA2B ○ ITA5 ○ ITB2 ○

Tabel S14: Selected WikiPathways [76] related to proteins with differential abundance in TPPexp Timepoint 6h vs Baseline all.

3.6 Protein interactions

3.6.1 Protein interactions in TPPexp Timepoint 6h vs Baseline all

Selected protein-protein interaction analysis using the STRING database ([22], http://string-db.org) revealed several direct as well as indirect interactions of the proteins featuring noteworthy differences in protein abundance, according to reduced logFC and p value thresholds as defined in section 3.2.8, within the comparison TPPexp Timepoint 6h vs Baseline all (Figure S18).

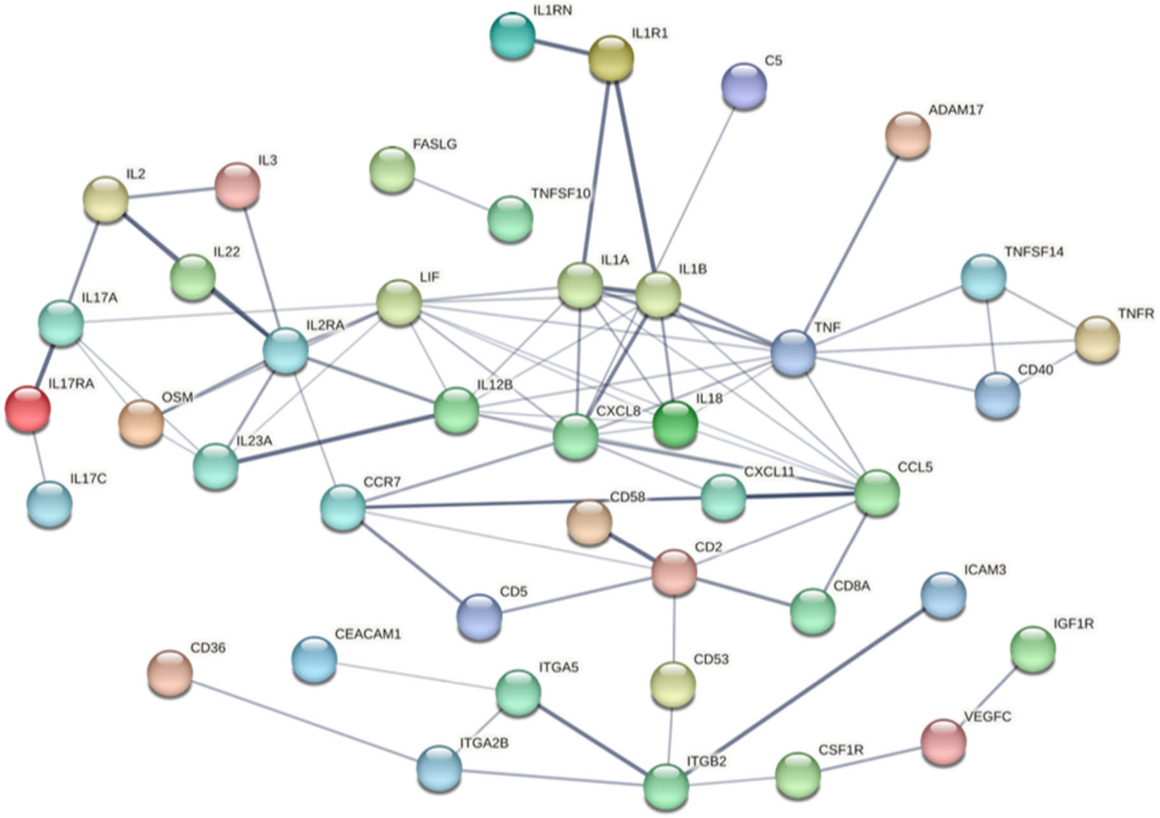

Figure S18: Selected protein interaction analysis of the significantly differential as well as noteworthy proteins with a confidence score above 0.4 using STRING. Protein names are displayed in HGNC nomenclature.

4 References

[22] D. Szklarczyk, A. L. Gable, K. C. Nastou, D. Lyon, R. Kirsch, S. Pyysalo, N. T. Doncheva, M. Legeay, T. Fang, P. Bork, L. J. Jensen, and C. von Mering. The STRING database in 2021: customizable protein–protein networks, and functional characterization of user-uploaded gene/measurement sets. Nucleic Acids Research, 49:D605–D612. doi: 10.1093/nar/gkaa1074. URL: https://academic.oup.com/nar/article/49/D1/D605/6006194.

[21] The UniProt Consortium. UniProt: the universal protein knowledgebase in 2021. Nucleic Acids Research, 49:D480–D489. doi: 10.1093/nar/gkaa1100. URL: https://academic.oup.com/nar/article/49/D1/D480/6006196.

[73] C. Schröder, A. Jacob, S. Tonack, T. P. Radon, M. Sill, M. Zucknick, S. Rüffer, E. Costello, J. P. Neoptolemos, T. Crnogorac-Jurcevic, A. Bauer, K. Fellenberg, and J. D. Hoheisel. Dual-color proteomic profiling of complex samples with a microarray of 810 cancer-related antibodies. Molecular & Cellular Proteomics, 9(6):1271–1280. doi: 10.1074/mcp.M900419-MCP200. URL: https://www.mcponline.org/article/S1535-9476(20)34404-2/abstract.

[74] M. Kanehisa. KEGG: Kyoto encyclopedia of genes and genomes. Nucleic Acids Research, 28(1):27–30. doi: 10.1093/nar/28.1.27. URL: https://academic.oup.com/nar/article-lookup/doi/10.1093/nar/28.1.27.

[75] M. Gillespie, B. Jassal, R. Stephan, M. Milacic, K. Rothfels, A. Senff-Ribeiro, J. Griss, C. Sevilla, L. Matthews, C. Gong, C. Deng, T. Varusai, E. Ragueneau, Y. Haider, B. May, V. Shamovsky, J. Weiser, T. Brunson, N. Sanati, L. Beckman, X. Shao, A. Fabregat, K. Sidiropoulos, J. Murillo, G. Viteri, J. Cook, S. Shorser, G. Bader, E. Demir, C. Sander, R. Haw, G. Wu, L. Stein, H. Hermjakob, and P. D'Eustachio. The reactome pathway knowledgebase 2022. Nucleic Acids Research, 50:D687–D692. doi: 10.1093/nar/gkab1028. URL: https://academic.oup.com/nar/article/50/D1/D687/6426058.

[76] M. Martens, A. Ammar, A. Riutta, A. Waagmeester, D. Slenter, K. Hanspers, R. A. Miller, D. Digles, E. Lopes, F. Ehrhart, L. J. Dupuis, L. A. Winckers, S. Coort, E. L. Willighagen, C. T. Evelo, A. R. Pico, and M. Kutmon. WikiPathways: connecting communities. Nucleic Acids Research, 49:D613–D621. doi: 10.1093/nar/gkaa1024. URL: https://academic.oup.com/nar/article/49/D1/D613/5992285.

[79] M. Sill, C. Schröder, J. D. Hoheisel, A. Benner, and M. Zucknick. Assessment and optimisation of normalisation methods for dual-colour antibody microarrays. BMC bioinformatics, 11:556. doi: 10.1186/1471-2105-11-556.

[80] M. E. Ritchie, B. Phipson, D. Wu, Y. Hu, C. W. Law, W. Shi, and G. K. Smyth. limma powers differential expression analyses for RNA-sequencing and microarray studies. Nucleic Acids Research, 43 (7):e47–e47. doi: 10.1093/nar/gkv007. URL: http://academic.oup.com/nar/article/43/7/e47/2414268/limma-powers-differential-expression-analyses-for.
